# TRMT6/61A-dependent base methylation of tRNA-derived fragments regulates gene-silencing activity and the unfolded protein response in bladder cancer

**DOI:** 10.1038/s41467-022-29790-8

**Published:** 2022-04-20

**Authors:** Zhangli Su, Ida Monshaugen, Briana Wilson, Fengbin Wang, Arne Klungland, Rune Ougland, Anindya Dutta

**Affiliations:** 1 Department of Genetics, University of Alabama, Birmingham, AL 35233 USA; 2 Department of Biochemistry and Molecular Genetics, School of Medicine, University of Virginia, Charlottesville, VA 22901 USA; 3 Department of Microbiology, Oslo University Hospital Rikshospitalet, 0372 Oslo, Norway; 4 Department of Molecular Medicine, Institute of Basic Medical Sciences, University of Oslo, 0317 Oslo, Norway; 5 Department of Surgery, Baerum Hospital Vestre Viken Hospital Trust, 1346 Gjettum, Norway; 6 Department of Biosciences, Faculty of Mathematics and Natural Sciences, University of Oslo, P.O. 10 Box 1066 Blindern, 0316 Oslo, Norway

**Keywords:** Small RNAs, RNA sequencing, RNA, Bladder cancer, RNA modification

## Abstract

RNA modifications are important regulatory elements of RNA functions. However, most genome-wide mapping of RNA modifications has focused on messenger RNAs and transfer RNAs, but such datasets have been lacking for small RNAs. Here we mapped N^1^-methyladenosine (m^1^A) in the cellular small RNA space. Benchmarked with synthetic m^1^A RNAs, our workflow identified specific groups of m^1^A-containing small RNAs, which are otherwise disproportionally under-represented. In particular, 22-nucleotides long 3′ tRNA-fragments are highly enriched for TRMT6/61A-dependent m^1^A located within the seed region. TRMT6/61A-dependent m^1^A negatively affects gene silencing by tRF-3s. In urothelial carcinoma of the bladder, where TRMT6/61A is over-expressed, higher m^1^A modification on tRFs is detected, correlated with a dysregulation of tRF targetome. Lastly, TRMT6/61A regulates tRF-3 targets involved in unfolded protein response. Together, our results reveal a mechanism of regulating gene expression via base modification of small RNA.

## Abstract (web summary)

RNA modifications are important regulators of RNA biology. Here we report N^1^-methyladenosine (m^1^A) enrichment on 22-nucleotide tRNA fragments and its effect on gene-silencing. Higher level of m^1^A in bladder cancer is accompanied by gene dysregulation in unfolded protein response.

## Introduction

RNA modifications are important regulators of RNA biology. In particular, recent advances in epitranscriptome mapping uncovers messenger RNA (mRNA) modifications including but not limited to N^6^-methyladenosine (m^6^A), inosine, pseudouridine, 5-methylcytidine (m^5^C), N^1^-methyladenosine (m^1^A), N^4^-acetylcytidine (ac^4^C) and 7-methylguanosine (m^7^G)^1–6^. These modifications regulate base pairing and/or protein-binding and thus play vital roles in regulating mRNA functions such as protein translation, mRNA stability, splicing, phase separation, virus infection and immune response. Transfer RNAs (tRNAs) also harbor a wide array of modifications, which often poses a challenge to sequencing the full-length tRNAs. Recently developed modification-friendly sequencing workflows have enabled better quantification of tRNA modification levels and tRNA abundance^7–14^, lending more evidence that RNA modifications are not static and could be associated with diseases. Analogous to modifications on DNAs and histones, RNA modifications were found to be specific and subjected to dynamic regulation and may be reversible. One great example is the use of pseudouridine modification to improve mRNA stability and translational efficiency for COVID-19 vaccine development^15–17^. The field of RNA modification is poised to expand greatly due to the introduction of new techniques for mapping modifications, especially when coupled with high-throughput sequencing (HTS).

Besides modifications on mRNAs and tRNAs, modifications on small non-coding RNAs of around 15–30 nucleotides length could have important functions. By far most small RNA research has been focused on microRNAs, as they are well-known regulators to fine tune gene expression via base pairing with target RNAs in Argonaute RNA-induced silencing complex (RISC)^18^. microRNAs can be regulated by 3′ end modifications and A-to-I editing^19^, with more recent but limited evidence of base modifications such as 7-methylguanosine and 8-oxoguanosine^20,21^. As another emerging group of small RNAs in this size range, tRNA-derived fragments (tRFs) were first identified via HTS methods about 12 years ago and represent abundant small RNAs with diverse biological functions^22,23^. For example, specific tRFs could regulate target gene expression via Argonaute-dependent mechanism^24–27^. In one such case, the host plant Argonaute was hijacked by the rhizobial tRFs to promote symbiosis^28^. In support of this notion, microRNA-sized tRFs were detected in complexes with Argonaute and GW182/TNRC6 proteins along with complementary mRNAs^24,25,29^. tRFs have also been implicated in mediating transposable element activity^30^, transgenerational inheritance^31,32^, ribosome biogenesis^33^, cell proliferation and differentiation^34,35^, and more. Nevertheless, to what extent tRFs inherit modifications from the parental tRNAs and how RNA modifications regulate tRF functions is still largely unknown.

N^1^-methyladenosine (m^1^A) is one of the modifications that has received more attention in mRNAs and tRNAs but have not been comprehensively profiled in the small RNAs. m^1^A disrupts regular Watson-Crick base pairing, increases local positive charge, and thus is highly likely to play regulatory functions. Indeed, m^1^A is enriched on specific regions and motifs on mRNAs and is found to decrease protein translation from a mitochondria mRNA^36–38^. Most recently, through directed evolution approach, HIV-1 reverse transcriptase was engineered to facilitate the mapping of hundreds of m^1^A mRNA sites^39^. Furthermore, m^1^A on both mRNAs and tRNAs are dynamically regulated in response to various stresses or conditions^14,36,37,40,41^, suggesting this modification may be regulated. Indeed, ALKBH1, ALKBH3, and FTO have been found to have m^1^A demethylation activity^40,42,43^.

Here we focus on profiling m^1^A base modification of the mammalian small RNAs by combining antibody enrichment and reverse-transcriptase-induced mismatch signature. We found specific groups of small RNAs that harbor high degree of m^1^A modification, which are otherwise disproportionally under-represented in regular small RNA cloning protocol. In particular, the 22-nucleotide long 3′ fragments from tRNAs (tRF-3b) are enriched in m^1^A modification at their fourth position, a modification that is dependent on the TRMT6/TRMT61A methyltransferase complex. Interestingly, m^1^A modification within the seed region of tRFs negatively affects gene-silencing activity of the tRNA fragments, and regulates global gene expression via small RNA activity. In urothelial carcinoma of the bladder, higher TRMT6/61A expression is accompanied by higher m^1^A modification on tRF-3b, accompanied by dysregulation of tRF targeted mRNAs. TRMT6/61A-dependent m^1^A regulates the unfolded protein response via tRF-3 targets. Our results reveal a mechanism of regulating gene expression via changes in tRF base modification.

## Results

### Systematic mapping of m^1^A sites in small RNA space

**Fig. 1:**
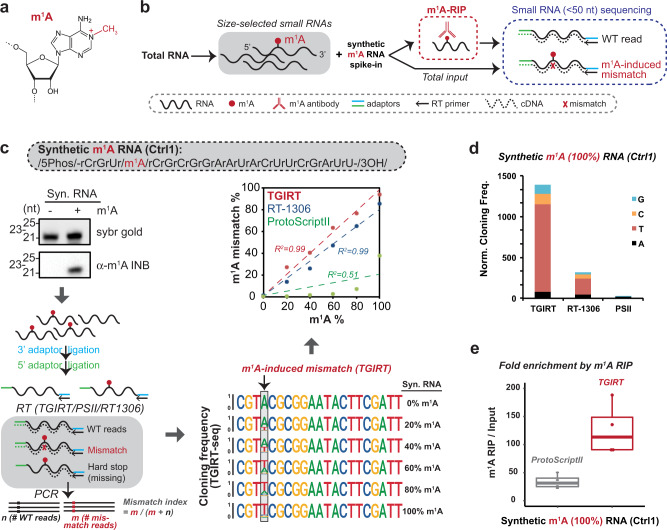
Systematic mapping of m^1^A sites in small RNA space. **a** Chemical structure of m^1^A (N^1^-methyladenosine). **b** Overall m^1^A mapping strategy for small RNAs by combining m^1^A RIP and m^1^A-induced mismatch analysis. Synthetic m^1^A RNA is included as a positive control. **c** TGIRT identified as the optimal reverse transcriptase for m^1^A-induced mismatch analysis. Briefly, synthetic m^1^A-containing RNA at different m^1^A stoichiometry was sequenced by three different reverse transcriptases (RTs): TGIRT (thermostable group II intron reverse transcriptase), PSII (ProtoScriptII, retrovirus RT) and RT-1306 (engineered HIV RT). For each RT, mismatch rate was calculated across all the reads that map to the synthetic RNA sequence (allowing 1 mismatch) as represented by the sequence logo. The mismatch rate at the known m^1^A site is then plotted against known m^1^A stoichiometry (*R*^2^ derived from linear fitting forced to cross intercept at zero). **d** ProtoScriptII leads to under-representation of m^1^A-containing RNAs. Cloning frequency is normalized to spike-ins. **e** m^1^A RIP successfully enriches synthetic m^1^A-containing RNA compared to input, when spiked in with total short RNAs. TGIRT captures enrichment better than ProtoScriptII. Data are based on four independent RIP experiments (two HEK293T and two U251). Boxplot center represents median, bounds represent 25 and 75%, and whiskers show the minimum or maximum no further than 1.5 * interquartile range from the bound. See also Supplementary Fig. 1 and Supplementary Table 1. Source data are provided as a Source Data file.

m^1^A is known to interrupt regular Watson-Crick A:U base pairing (Fig. 1a) and cause reverse transcriptase (RT) stalling during the preparation of libraries for HTS^36–39^. This feature can be utilized to map m^1^A sites by mismatch (misincorporation) at m^1^A positions. To systematically map potential m^1^A sites in small RNAs (<50 nucleotides long), we combine two independent approaches (Fig. 1b): m^1^A antibody enrichment followed by small RNA-sequencing (m^1^A-RIP-seq) and m^1^A-induced mismatch signature by sequencing. To first identify the m^1^A-compatible RT for standard small RNA-sequencing, three candidate RTs (ProtoScriptII—a commonly used M-MuLV RT, TGIRT—template-switching group II reverse transcriptase, and engineered HIV RT-1306) were tested with a synthetic small RNA with single m^1^A site (Fig. 1c). TGIRT and RT-1306 were selected for testing based on their previous success in sequencing m^1^A-containing mRNAs and tRNAs (Li et al. 2017; Safra et al. 2017; Zhou et al. 2019; Behrens et al. 2021). The experimental workflow (Fig. 1c, left bottom) was as follows: the small RNAs were first ligated with 3′ end adaptors and then 5′ end adaptors, followed by primer-initiated RT reaction to produce cDNA, which was then PCR amplified by primers covering both adaptor sequences. Our strategy ensures only the read-through products will be cloned and used for downstream mismatch analysis for m^1^A modification estimation. By including 5′ adaptor ligation before the RT step, we eliminate the possibility of any RT stalling product to be cloned as a truncated product due to the lack of complementarity to the 5′ primer for PCR amplification. Such an approach is preferred for the small RNAs since they often differ by only a few bases, especially for tRFs. For example, 18-nt tRF-3 and 22-nt tRF-3 have been shown to regulate LTR functions via different mechanisms^30^. Using this workflow, mismatch index (calculated by A to T/G/C mutation) at the synthetic m^1^A site shows great sensitivity and dynamic range over different m^1^A stoichiometry for both TGIRT and RT-1306 (Fig. 1c and Supplementary Fig. 1a, Supplementary Table 1). However, ProtoScriptII, the reverse transcriptase commonly used for short RNA sequencing, produces lower than 5% misincorporation when m^1^A is present in <=60% of the synthetic short RNA (Fig. 1c and Supplementary Fig. 1b), and leads to a lower cloning frequency compared to TGIRT and RT-1306 as can be seen when 100% of the RNAs have m^1^A (Fig. 1d). This result is most likely due to stalling of the ProtoScripII RT at the m^1^A site and suggests that m^1^A-containing small RNAs are under-represented in most small RNA-sequence libraries that commonly uses this RT. As a control to measure background mutation rate, mismatch was measured using unmodified synthetic short RNA, which shows <2% misincorporation for all three RTs, especially notable is the ~0.2% misincorporation for TGIRT (Supplementary Fig. 1c and Supplementary Table 1). Lastly, when combined with m^1^A antibody enrichment (m^1^A-RIP), both TGIRT and ProtoScriptII are able to detect enrichment of synthetic m^1^A RNAs spiked into cellular small RNAs (Fig. 1e and Supplementary Fig. 1d, Supplementary Table 1). Based on the above analysis, we selected TGIRT to further identify endogenous m^1^A-containing small RNAs.

### Specific tRNA-derived fragments are highly enriched for m^1^A

**Fig. 2:**
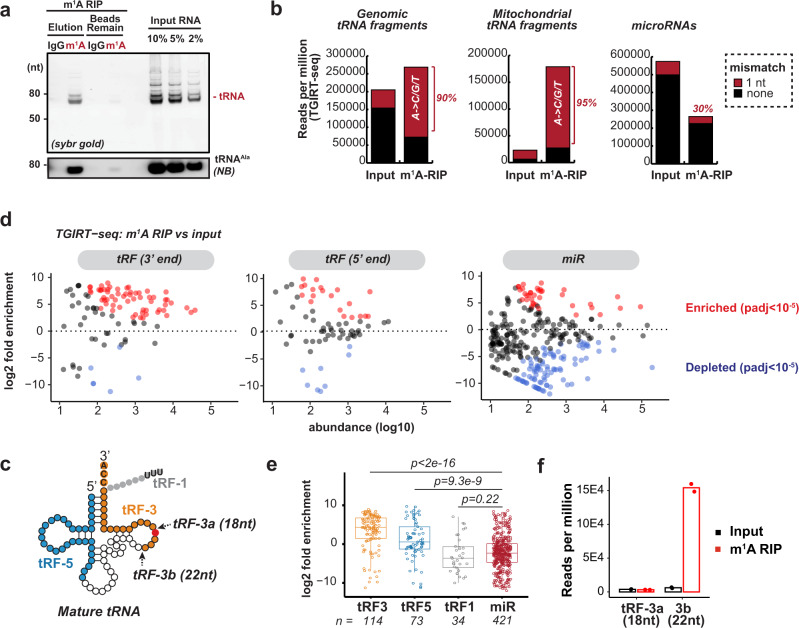
Specific tRNA-derived fragments are highly enriched for m^1^A. **a** m^1^A RNA immunoprecipitation (RIP) successfully enriches for tRNAs compared to IgG control RIP, by both Sybr gold staining and Northern blot against tRNA^Ala^. **b** m^1^A RIP enriches for reads that contain A->C/G/T mismatch in tRNA fragments. Bar graph represents RPM (reads per million) values for each group of small RNAs allowing no mismatch (black) and additional mapped reads when allowing 1-nt mismatch (red). Labeled percentage shows the percentage of A->C/G/T mismatch among all mismatches. **c** Major types of tRFs include tRF-3s (3′ end of mature tRNAs), tRF-5s (5′ end of mature tRNAs) and tRF-1s (trailer of precursor tRNAs). tRF-3s have two major isoforms, tRF-3b (22-nt) and tRF-3a (18-nt). **d**, **e** tRF-3s are significantly enriched by m^1^A RIP. **d** Log2 fold enrichment (m^1^A RIP/input) was plotted against small RNA abundance for different RNA groups. Only significantly (p.adj < 10^−5^ by DESeq2 Wald test with multiple hypothesis adjustment) enriched or depleted RNAs were colored. **e** tRF-3s (*n* = 114) and tRF-5s (*n* = 73), but not tRF-1s (*n* = 34) are significantly more enriched by m^1^A RIP than miRs (*n* = 421) (*p* value by two-sided Wilcoxon test compared to miRs). Boxplot center represents median, bounds represent 25 and 75%, and whiskers show the minimum or maximum no further than 1.5 * interquartile range from the bound. **f** Bar graph shows 22-nt tRF-3b but not 18-nt tRF-3a are significantly enriched by m^1^A RIP compared to input. Data in this figure are based on TGIRT-seq of two independent RIP experiments in HEK293T. See also Supplementary Fig. 2. Source data are provided as a Source Data file.

To identify endogenous m^1^A-containing small RNAs, m^1^A RIP was applied to purified short RNAs from cells. m^1^A-modified tRNAs from HEK293T cells were successfully enriched by m^1^A RIP and eluted (Fig. 2a). Subsequent TGIRT-seq of both input and m^1^A RIP RNAs (15–50 base long) revealed that tRNA-derived fragments (both genomic and mitochondria-encoded; tRFs) are relatively enriched among short RNAs (Supplementary Fig. 2a). The enrichment by m^1^A RIP is more prominent for the tRF reads that have one base mismatch, presumably at the site of the m^1^A modification (Fig. 2b). This mismatch-associated enrichment by m^1^A RIP was not observed for microRNAs or other small RNAs (Fig. 2b and Supplementary Fig. 2b). Majority of these mismatch-containing tRF reads have A to C/G/T mismatch (90% for cytoplasmic tRFs and 95% for mitochondria tRFs) after m^1^A RIP (Fig. 2b), suggesting specific enrichment of modified (mismatch-prone) adenosine, most likely m^1^A. Further analysis by ProtoScriptII and two different m^1^A-specific antibodies (one from MBL and one from Abcam, both previously characterized for their m^1^A-binding specificity^44^) also confirms that the tRFs are most enriched by m^1^A RIP (Supplementary Fig. 2c and Supplementary Table 2), suggesting that they bear m^1^A modifications.

**Fig. 3:**
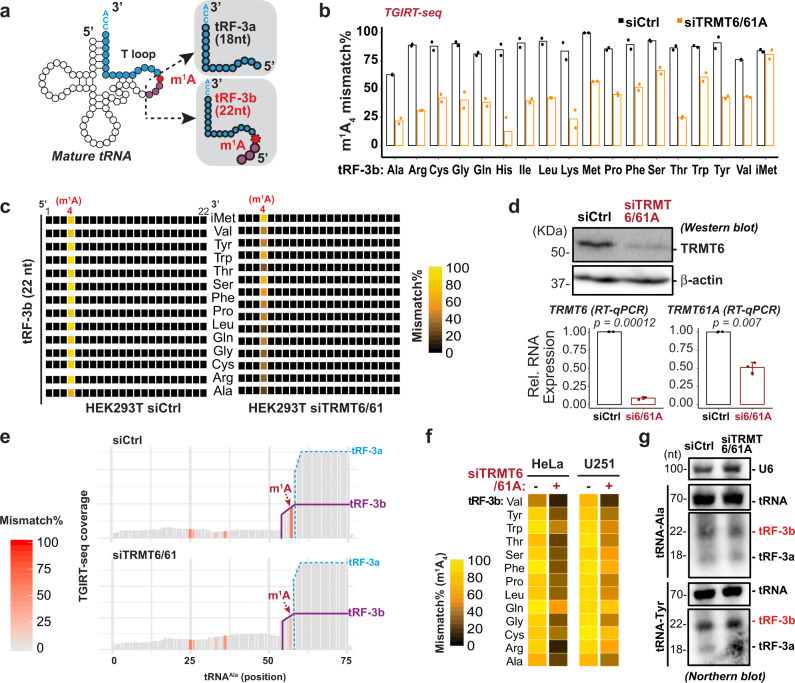
m^1^A on 22-nucleotides 3′ tRNA fragments is dependent on TRMT6/61A. **a** Scheme of 3′ tRNA fragments (tRF-3s) that are derived from 3′ end of mature tRNAs. tRF-3b indicates 22-nt tRF-3s, and tRF-3a indicates 18-nt tRF-3s. **b**–**e** m^1^A mismatch on 22-nt tRF-3bs are globally regulated by TRMT6/61A. **b**, **c** Mismatch rate is calculated for the A4 position on tRF-3bs, based on TGIRT-seq of two independent knock-down experiments in HEK293T. **d** Knock-down efficiency is confirmed by western blot and RT-qPCR. RT-qPCR data was represented as mean ± SD (*p* value based on two-tailed paired student’s t test from three independent knock-down experiments). **e** Coverage plot of all tRF reads mapped on the parental tRNA^Ala^ shows decrease of m^1^A of tRF-3b but not tRF-3 abundance (TGIRT-seq in HEK293T, one replicate shown as example). **f** m^1^A mismatch on A4 position of tRF-3b is also decreased by siTRMT6/61A in HeLa and U251, based on TGIRT-seq. **g** Northern blot confirms detection of both tRF-3a and tRF-3b isoforms, which are not altered by siTRMT6/61A in HEK293T. Full-length tRNA^Ala^, tRNA^Tyr^ and U6 are probed as a loading control. See also Supplementary Fig. 3. Source data are provided as a Source Data file.

Based on the start and end positions, tRNA fragments can be separated into three major groups (Fig. 2c)^22,30,34^: (1) tRF-3s that map to the extreme 3′ CCA end of mature tRNAs, (2) tRF-5s that map to the extreme 5′ end of mature tRNAs, and (3) tRF-1s that contain the trailer sequence (often ends with poly-U) from the precursor tRNAs. In particular, 3′ tRFs have been shown to have two major species: tRF-3a (18 nt) and tRF-3b (22 nt), which are recapitulated by TGIRT-seq (Supplementary Fig. 2d). tRFs from 3′ end of mature tRNAs are preferentially enriched by m^1^A antibody, whereas the ones from 5′ end are mildly enriched and tRF-1s not enriched compared to microRNAs (Fig. 2d, e). Furthermore, 22-nt tRF-3b are specifically enriched by m^1^A antibody in contrast to the 18-nt tRF-3a (Fig. 2f and Supplementary Fig. 2e), suggesting only the 22-nt but not the 18-nt tRF-3s harbor m^1^A. Notably, the enrichment of tRF-3b (Fig. 2f) is to the similar extent as that of the spike-in control m^1^A RNAs (Fig. 1e and Supplementary Fig. 1d). The TGIRT misincorporation signature from the input samples also yielded the location and stoichiometry of m^1^A on specific tRF-3s. The highest mismatch rate was observed at the fourth position (A_4_) of all 22-nt tRF-3b (Fig. 3b, c). This high misincorporation rate is highly specific: not observed for other positions on tRF-3b (Fig. 3c), or on the 18-nt tRF-3a. Interestingly, some microRNAs were significantly enriched by m^1^A RIP (Fig. 2d and Supplementary Table 2), but failed to show site-specific mismatch pattern, thus we did not report them as bona fide m^1^A-containing small RNAs in this report (further discussed in Discussion). All of these indicate tRF-3b are m^1^A-containing small RNAs.

Mitochondria tRFs are also enriched by m^1^A RIP, particularly 5′ fragments from most mitochondria tRNAs (Supplementary Fig. 2f), presumably due to the abundant m^1^A at A_9_ position of mitochondrial tRNA^9,36^. The three 5′ halves that are not enriched by m^1^A RIP are from tRNA^Cys^, tRNA^Met^, and tRNA^Tyr^ (Supplementary Fig. 2f); tRNA^Cys^ and tRNA^Tyr^ have m^1^G_9_ while tRNA^Met^ has U_9_, so none of them has m^1^A_9_^13^. The misincorporation signature by TGIRT confirms that mitochondria tRFs such as 5′ half from tRNA^Lys^ have high mismatch rate at the m^1^A_9_ site (Supplementary Fig. 2g). Since most of the m^1^A sites identified are at high stoichiometry, we skipped m^1^A RIP and only used TGIRT mismatch signature to quantify m^1^A at specific sites for the following analysis.

### m^1^A on 22-nucleotides 3′ tRNA fragments is dependent on TRMT6/61A

The A_4_ position on 22-nt tRF-3b corresponds to the A_58_ position on mature tRNAs (Fig. 3a). m^1^A_58_ on cytoplasmic tRNAs is catalyzed by a heterodimer enzyme complex TRMT6/TRMT61A (or GCD10/GCD14 in yeast). To test whether the m^1^A on tRF-3b was a modification that was introduced by TRMT6/61A (likely on the parental tRNA), the misincorporation signature was assessed after TRMT6/61A knockdown (Fig. 3b–e). m^1^A-dependent misincorporation on tRF-3s is decreased globally except tRF-3s from tRNA^iMet^ (Fig. 3b, c) when TRMT6/61A is knocked down (Fig. 3d). The lower tRF-3b A_4_ misincorporation rate by siTRMT6/61A or siTRMT61A alone is also observed in HeLa and U251 cells (Fig. 3f and Supplementary Fig. 3a, b). Meanwhile, TRMT6/61A does not affect other m^1^A sites, such as the m^1^A_9_ on tRF-5s or 5′ halves from mitochondria tRNA^Lys^ and tRNA^Glu^ (Supplementary Fig. 3c). Although the cloning frequency of tRF-3b with ProtoScriptII is increased significantly after TRMT6/61A knock-down (Supplementary Fig. 3d–g), Northern blots do not detect any change in the steady-state levels of tRF-3b and tRNAs (Fig. 3g and Supplementary Fig. 8). This corroborates that m^1^A-bearing small RNAs are under-represented by this commonly used ProtoScriptII RT.

### m^1^A attenuates gene-silencing by tRF-3s

**Fig. 4:**
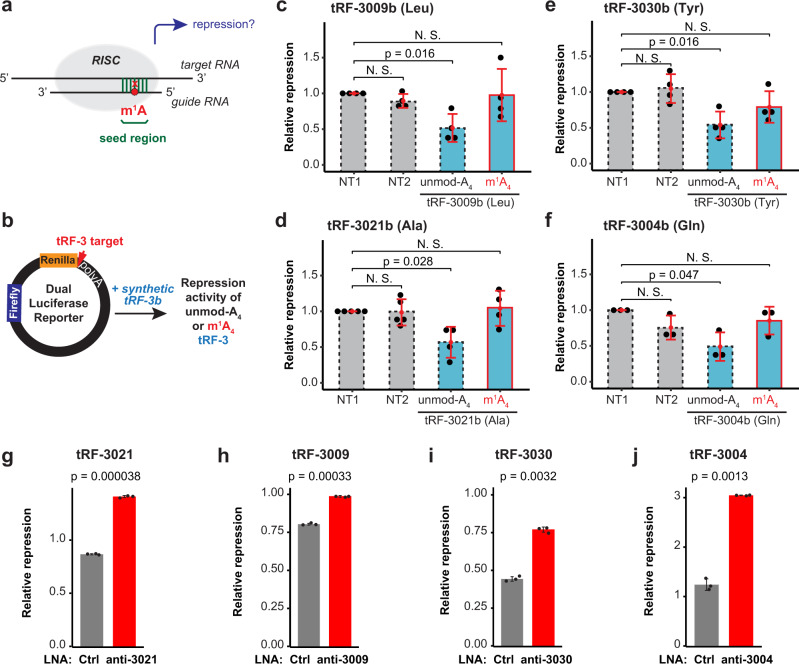
m^1^A attenuates gene silencing by tRF-3s. **a** m^1^A is located within the seed region of tRF-3b, which represents the guide RNA in RISC (RNA-induced silencing complex). m^1^A may interfere with base pairing between tRF-3b and the target RNA, thus alleviate target repression. **b** Scheme of the dual luciferase reporter assay to measure tRF-3 gene-silencing activity. tRF-3b complementary target sequence is inserted in the 3′ UTR of *Renilla* luciferase (Rluc) gene. *Firefly* luciferase (Fluc) signal is used for normalization. Synthetic tRF-3b with unmodified A_4_ or m^1^A-modified A_4_ is co-transfected with the reporter plasmid to measure relative effect of tRF-3b on the reporter activity. **c**–**f** Relative tRF-3 gene-silencing activity is measured by the dual luciferase reporter assay in HEK293T. Relative activity is calculated from Rluc signal divided by Fluc signal, and normalized to the empty site reporter; NT1 (non-targeting control1) is set as 1 in each replicate. **g**–**j** Relative tRF-3 gene-silencing activity is de-repressed after tRF-3 knock-down by LNAs (lock nucleic acids). Relative activity is calculated from Rluc signal divided by Fluc signal and normalized to the empty site reporter. Non-targeting LNA was used as control (Ctrl). All data are represented as mean ± SD; *p* value based on two-tailed paired student’s t test from three (four for **d**, **e**) independent experiments. Source data are provided as a Source Data file.

The presence of m^1^A at a specific location on tRF-3s poses an intriguing possibility that it might regulate tRF-3 function, especially if it involves base pairing or protein binding. tRF-3s have been found in diverse biological pathways, in particular gene-silencing pathways that rely on base pairing between the guide small RNAs (tRF-3s) and the target RNAs (Fig. 4a). These Ago-dependent gene-silencing mechanisms may be influenced by m^1^A at position 4 of tRF-3s, which falls into the seed region essential for base pairing with target RNAs^25,45^.

To directly test the effect of m^1^A on tRF-3b gene-silencing activity, we generated dual luciferase reporters that have tRF-3 target sites in 3′ UTR of Renilla luciferase gene, and can be normalized to Firefly luciferase signal (Fig. 4b). Only A_4_-unmodified tRF-3b triggers gene-silencing, whereas the m^1^A_4_-modified tRF-3b abolishes such gene-silencing (Fig. 4c–f). This effect was observed for four different tRF-3b sequences coming from different amino acid groups (tRF-3009b from tRNA^Leu^, tRF-3021b from tRNA^Ala^, tRF-3030b from tRNA^Tyr^ and tRF-3004b from tRNA^Gln^).

To ensure that repression by tRF was not an artefact from over-expression, we also knocked down endogenous tRF-3 by LNA (locked nucleic acid). tRF-3021 reporter activity was de-repressed specifically by anti-tRF-3021 (Fig. 4g), but not anti-tRF-3009, in a concentration-dependent manner (Supplementary Fig. 4a). Similarly, tRF-3009, tRF-3030 and tRF-3004 reporter activity was de-repressed by the corresponding LNAs (Fig. 4h–j).

Overall this suggests that m^1^A attenuates gene-silencing by tRF-3b due to problems with the seed annealing to the target mRNA.

### Argonaute association of tRF-3b is not decreased by TRMT6/61A-dependent m^1^A

**Fig. 5:**
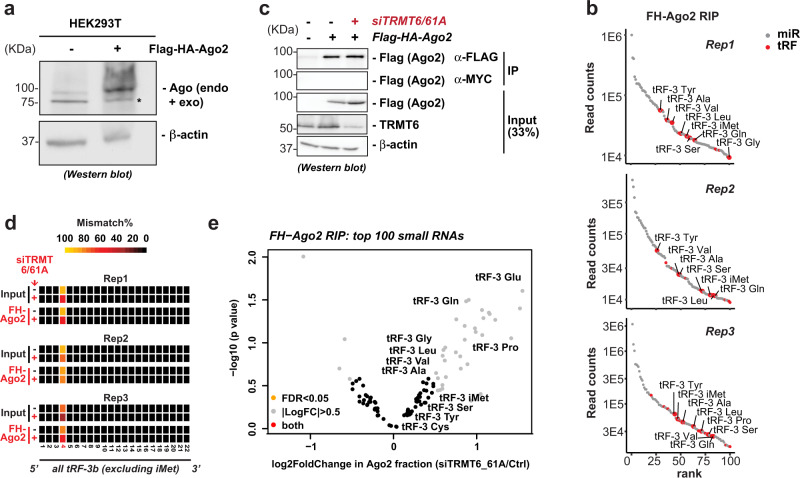
Argonaute association of tRF-3b is not decreased by TRMT6/61A-dependent m^1^A. **a** Construction of stable FH-Ago2 cells, detected by anti-pan-Ago western blot (*indicates non-specific band detected by the antibody). The pan-Ago band detects both endogenous Ago proteins and tagged FH-Ago2 protein. **b** Top 100 ranking small RNAs (gray dots: microRNAs, red dots: tRFs) detected in Ago2 RIP (RNA immunoprecipitation) are plotted (*Y*-axis: read counts on log10 scale). **c** Ago2 RIP was performed in HEK293T siCtrl and siTRMT6/61A. **d** TGIRT-seq of the Ago2-bound and input fractions identified lower m^1^A mismatch on tRF-3b. The color-shaded mismatch rate was calculated for all tRF-3b excluding the one from tRNA^iMet^. **e** Volcano plot of the Ago2-bound small RNA changes upon siTRMT6/61A (differential analysis and *p* value by DESeq2 Wald test). Data in this figure are based on TGIRT-seq of three independent Ago2 RIP experiments in HEK293T stable Flag-HA-Ago2 cells. See also Supplementary Fig. 4 and Supplementary Table 3. Source data are provided as a Source Data file.

Because m^1^A on tRF-3b prevents silencing by tRF-3b (Fig. 4), we tested whether the modification decreased the association of endogenous tRF-3s with Ago2. To do this we created a cell line stably expressing Flag-HA-tagged Ago2 (Fig. 5a). TGIRT-seq identified tRF-3s among the top 100 Ago2-associated small RNAs (Fig. 5b and Supplementary Table 3). Upon knockdown of TRMT6/61A (Fig. 5c), the percent of mismatch in the reads, that corresponds to the m^1^A modification, was (a) decreased in the Ago2-associated fraction but (b) not enriched or depleted in the Ago2-associated fraction relative to the input (Fig. 5d and Supplementary Table 4). Thus TRMT6/61A regulates m^1^A on tRF-3s in both pools equally, and the m^1^A modification does not preclude tRF-3b from entering into a complex with Argonaute. There is a small (1–3 fold) increase of several tRF-3s in the Ago2-associated fraction upon knockdown of TRMT6/61A, but the increase does not cross the FDR threshold of <0.05 (Fig. 5e and Supplementary Fig. 4b). The total tRF-3 levels (input) are mostly unaltered by siTRMT6/61A (Supplementary Fig. 4c, Supplementary Table 3). The lack of any change in the tRF-3 levels in the Ago-associated fraction relative to the input fraction suggests the TRMT6/61-dependent m^1^A modification on the tRF-3 seed region does not dramatically affect direct Ago binding. This is consistent with the known Ago2 binding mode with the short guide RNA in the human Ago2-siRNA co-crystal structure^46^, from which we modeled m^1^A into the 4^th^ position of guide RNA: the modeled structure shows Ago2 protein interacts with the RNA backbone and does not interact with the base (Supplementary Fig. 4d). Interestingly, although the percent of total tRF-3b mismatch (m^1^A modification) in both input and Ago-bound fractions is regulated by TRMT6/61A (Fig. 5d), this is not observed for tRF-3b from tRNA^iMet^ (Supplementary Table 3). Taken together, m^1^A modified tRF-3s do not decrease their Ago association and so the attenuation of gene-silencing is most likely because of decreased base-pairing with target mRNA (Fig. 4).

### m^1^A-dependent changes in global gene silencing by tRF-3b

**Fig. 6:**
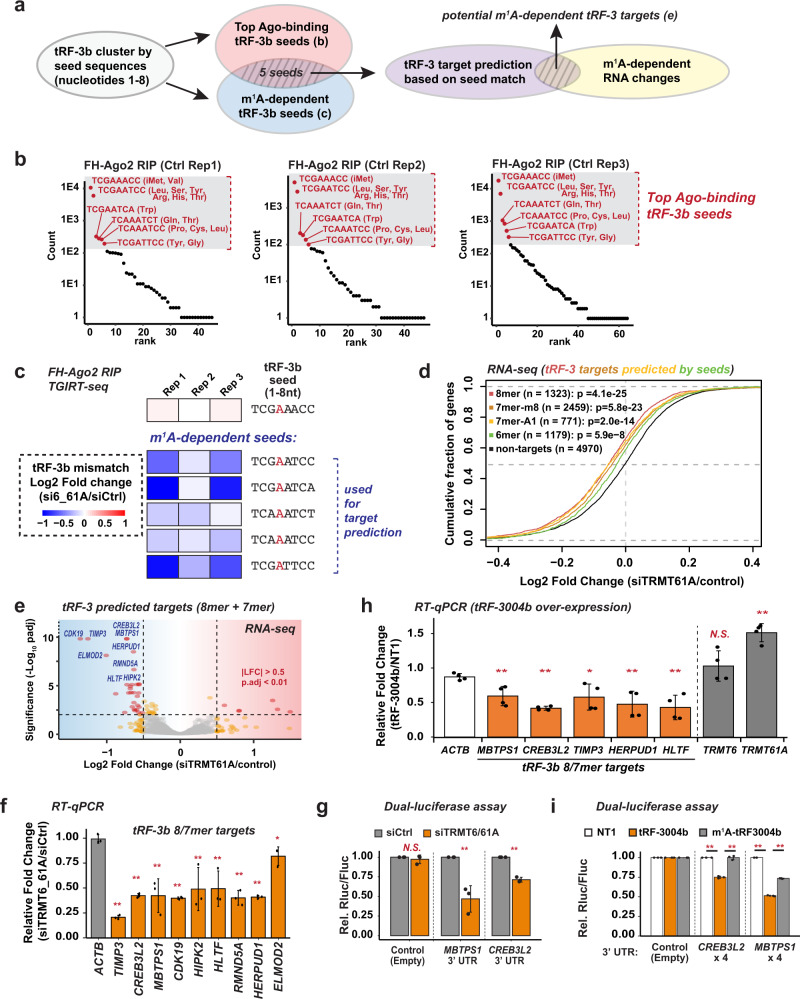
m^1^A-dependent changes in global gene silencing by tRF-3b. **a** Workflow to identify potential tRF-3 target RNAs that could be regulated by m^1^A status. **b** Ago-binding tRF-3b seeds sequences are identified from Ago2-RIP. **c** m^1^A-dependent tRF-3b seed sequences are identified from Log2 Fold Change of the m^1^A mismatch rate for the Ago2-bound fractions upon TRMT6/61A knockdown, as indicated by the color. **d** tRF-3 targets are globally repressed compared to the non-targets upon siTRMT61A in HEK293T. Distribution of expression changes (*X*-axis: Log2 fold change from RNA-seq) is visualized by CDF (Cumulative Distribution Function) plot. *P* value by one-sided Kolmogorov–Smirnov test to compare overall distribution between each target type versus non-targets. Significantly repressed tRF-3 targets (7/8-mers, DESeq2 adjusted *p* value <0.01, Log2FoldChange <−0.5) from (**e**) RNA-seq are selected for further validation by (**f**) RT-qPCR. **g** Dual-luciferase assay with tRF-3 target site was performed to measure the effect after siTRMT6/61A. *MBTPS1* and *CREB3L2* 3′ UTR sequences were inserted after *Renilla* luciferase gene. **h** tRF-3004b mimic over-expression represses tRF-3 targets by RT-qPCR. **i** Dual-luciferase assay was performed to measure the effect after tRF-3004b mimic over-expression. tRF-3004b target sites from endogenous *MBTPS1* and *CREB3L2* 3′ UTR were cloned as 4X tandem repeats. Data are based on **b**, **c** TGIRT-seq of three independent knock-down and Ago2 RIP experiments in Flag-HA-Ago2 HEK293T; **d**, **e** RNA-seq of two independent knock-down experiments; **f** *n* = 3, (**g**) *n* = 3, (**h**) *n* = 4, (**i**) *n* = 3 biologically independent experiments in HEK293T (mean ± SD). RT-qPCR: relative expression is normalized to siCtrl (**f**) or NT1 (non-targeting RNA control1 (**h**), and compared with *ACTB* expression. Dual luciferase assay: *Renilla* luciferase read is normalized to *Firefly* luciferase read and further normalized to siCtrl (**g**) or NT1 (**i**). The significance was based on student’s t test (two-tailed unpaired, *p < 0.05, **p < 0.01, N.S. = *p* > 0.05). Exact *p* values from left to right: **f** 9.6e−6, 9.1e−4, 0.0050, 2.2e−5, 0.018, 0.0095, 3e−4, 2.6e−5, and 0.042; **g** 0.49, 0.03, 0.0042; **h** 0.0097, 3.5e−6, 0.026, 0.0068, 0.003, 0.22, and 0.0001545; **i** 0.0011, 0.00063, 5.7e−5, and 4.1e−6. See also Supplementary Tables 4–5 and Supplementary Fig. 5. Source data are provided as a Source Data file.

Base pairing at positions 2–4 of guide RNAs is critical for Ago-mediated gene silencing^45^. m^1^A on tRF-3b locates specifically at the 4th position (Figs. 2 and 3). Since m^1^A interrupts canonical A:U base pairing, we hypothesize the weakened base pairing by m^1^A in the tRF-3 seed region with target RNA explains the lowered gene-silencing activity observed for m^1^A-containing tRF-3s (Fig. 4). To test this hypothesis, we first identified potential tRF-3 target RNAs that could be regulated by m^1^A status by the strategy summarized in Fig. 6a. Since seed sequences (5′ nucleotides 2–8) among tRF-3bs are highly similar even though they are derived from different parental tRNAs (example shown for one seed in Supplementary Fig. 5a), we rationalize that tRF-3bs with similar seeds will act together to silence the same genes, similar to microRNA families. We determined which seeds from the top Ago2-associated tRF-3b (Fig. 6b and Supplementary Table 4) were also the ones that showed the most decrease in m^1^A modification after knockdown of TRMT6/61A to determine the seeds most likely to suffer biologically significant interference with target prediction (Fig. 6c and Supplementary Table 4). We predict the targets for these seeds based on complementarity in the 3′ UTR. RNA-seq revealed that targets of these seeds are significantly repressed compared to the non-targets when TRMT6/61A is knocked down and the tRF-3b are hypomodified by m^1^A at the fourth position (Fig. 6d and Supplementary Table 5).

The targets predicted to have 8-mer and 7-mer matches were more significantly repressed than those that match with 6-mers in the seed (Fig. 6d, e). The repression of the most significantly repressed 8/7-mer tRF-3 targets (TIMP3, CREB3L2, MBTPS1, CDK19, HIPK2, HERPUD1, RMND5A, ELMOD2, and HLTF) were individually validated by qPCR after siTRMT6/61A (Fig. 6f and Supplementary Table 5). Notably, these tRF-3b seed sequences do not overlap with any expressed (read count >10) miRbase-annotated human microRNA seeds (Supplementary Fig. 5a), eliminating the possibility that the repression after TRMT6/61A knockdown is through the regulation of microRNAs. In particular, 78% of the predicted targets harbor more than one target sites (Supplementary Fig. 5b and Supplementary Table 5).

The TRMT6/61A-regulated tRF-3b target repression is also observed when each individual tRF-3b seed is separately used to predict targets (Supplementary Fig. 5c). To confirm that such gene repression is mediated by tRF-3 targeting the 3′ UTR, endogenous 3′ UTR sequence of *MBTPS1* with a single, evolutionarily conserved, tRF-3 target 8-mer site (Supplementary Fig. 5d) was cloned into a dual-luciferase reporter. MBTPS1 3′ UTR reporter is indeed repressed by siTRMT6/61A (Fig. 6g). Similar results were obtained with another 8-mer target gene, *CREB3L2* (Fig. 6g and Supplementary Fig. 5e). Both 8-mer sites were predicted by seed sequence “TCAAATCT”, represented by tRF-3b from tRNA^Gln^ (Fig. 6b and “seed2” in Supplementary Table 5). Consistent with the prediction, the siTRMT-dependent repression can be mimicked by over-expressing unmodified tRF-3004b from tRNA^Gln^ without decrease in TRMT6/61A expression (Fig. 6h), whereas m^1^A-modified tRF-3004b displayed significantly weaker ability to repress 3′ UTR reporters (Fig. 6i and Supplementary Fig. 5f). Overall, the results suggest that tRF-3s can regulate global gene expression by seed pairing with target 3′ UTRs and that this is interfered by m^1^A modification in tRF-3 seed region.

### Alteration of tRF-3 m^1^A levels and tRF-3 targets in bladder cancer

**Fig. 7:**
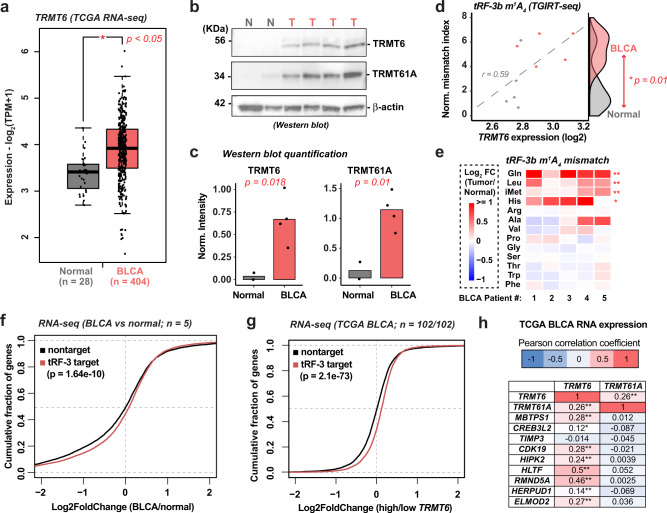
Alteration of tRF-3 m^1^A levels and tRF-3 targets in bladder cancer. **a** *TRMT6* expression in TCGA BLCA (*n* = 404) compared to the normal (*n* = 28, TCGA and GTEX). Expression (*Y*-axis) is derived from RNA-seq on Log2 scale. Data visualized by GEPIA2 (*p* value by one-way ANOVA). Boxplot center represents median, bounds represent 25 and 75%, and whiskers show the minimum or maximum no further than 1.5 * interquartile range from the bound. **b**, **c** TRMT6 and TRMT61A protein expression by Western blots in BLCA tumor versus normal samples, with β-actin as loading control. Relative protein expression is normalized to β-actin; *p* values based on two-tailed unpaired student’s t test. **d**, **e** m^1^A mismatch measured by TGIRT-seq in BLCA tumor samples and the paired normal (*n* = 5). **d** Overall tRF-3b normalized mismatch index (*y*-axis, index defined by mismatch% over non-mismatch%) is plot against *TRMT6* expression (*x*-axis, log2 scale from RNA-seq). Significantly higher tRF-3b mismatch index is observed in tumors (*p* value by paired student’s t test). **e** tRF-3b A4-mismatch% is compared between tumor and paired normal for each patient (red color in heatmap suggests higher mismatch% in tumor, *p* value by unpaired two-sided Wilcoxon test). **f**, **g** Global de-repression of tRF-3b targets observed in BLCA tumor compared to the paired normal (**f**, *n* = 5) or in high-*TRMT6* expressed (upper quartile) BLCA tumor (*n* = 102) compared to the low-*TRMT6* expressed (bottom quartile) tumors (n = 102) in TCGA (**g**). CDF (Cumulative Distribution Function) p value is calculated by one-sided Kolmogorov–Smirnov test to compare overall distribution between tRF-3 targets versus non-targets. **h** Correlation between *TRMT6/61A* expression and tRF-3 targets in TCGA BLCA patients (*n* = 404). Pearson correlation was based on mRNA expression levels in TPM (transcripts per million) by GEPIA2 (visualized on log2 scale). Correlation coefficient summarized by color (red = positive correlation, blue = negative correlation), *p* value from paired correlation test, *p < 0.05, **p < 0.01. Exact *p* values from up to down: **e** 0.0075, 0.0075, 0.0075, 0.025, 0.12, 0.66, 0.66, 0.66, 0.0075, 0.65, 0.66, 0.12, and 0.66; **h** TRMT6 – 9.1e−8, 7.8e−9, 0.019, 0.78, 8,4e−9, 1.2e−6, 0, 0, 0.005, 4.5e−8; TRMT61A – 9.1e−8, 0.81, 0.08, 0.37, 0.68, 0.94, 0.29, 0.96, 0.17, and 0.47. See also Supplementary Figs. 6, 7 and Supplementary Table 6. Source data are provided as a Source Data file.

It has been reported that some tRNA-modifying enzymes, including TRMT6/61A, are significantly upregulated in several cancer types^47^. Analysis of public cancer database TCGA reveals that *TRMT6* mRNA is indeed upregulated in multiple cancer types compared to respective normal controls (Supplementary Fig. 6a: red labels), but this was not as wide-spread for *TRMT61A* (Supplementary Fig. 6b). The *TRMT*6 upregulation is significant in urothelial carcinomas of the bladder (BLCA) (Fig. 7a). To examine whether TRMT6/61A-mediated tRF-3 modification observed in cell line models (Figs. 1–6) is also seen in tumor samples, we obtained matched tumor and normal samples from clinically diagnosed BLCA patients (Supplementary Table 6), in which we confirmed by RNA-seq and RT-qPCR that *TRMT6* RNA is over-expressed in tumor samples (Supplementary Fig. 7a, b). Here again, unlike *TRMT6*, *TRMT61A* RNA is relatively unchanged compared to the normal samples (Supplementary Fig. 7a–c). Most importantly, dramatic upregulation of both TRMT6 and TRMT61A protein levels in tumor samples was detected by western blotting (Fig. 7b, c). Small RNA TGIRT-seq in these tumor samples successfully identified m^1^A-mediated mismatch at the 4th position of tRF-3b, which is otherwise missed by using other RT such as ProtoScriptII that stalls at m^1^A (Supplementary Fig. 7d).

There was a positive correlation between *TRMT6* expression level and the overall m^1^A mismatch level of tRF-3b, and a significant increase of the m^1^A mismatch in the BLCA tumor samples that is consistent with the higher TRMT6/61A expression (Fig. 7d, e). Consistent with the higher levels of the interfering m^1^A modification, tRF-3b target RNAs predicted by seed-mer matches are upregulated compared to non-targets in BLCA tumor samples compared to normal in our experimental data (Fig. 7f: combined seeds and Supplementary Fig. 7e: individual seeds). In addition, in TCGA BLCA data, the tRF-3b targets are induced in the tumors with high level of TRMT6 (Fig. 7g). Interestingly, 8 out of the 9 tRF-3 targets repressed by siTRMT6/61A (Fig. 6e) showed a significant positive correlation in expression with *TRMT6* in TCGA bladder patient samples (Fig. 7h and Supplementary Fig. 7f). Taken together, these results suggest TRMT6/61A regulates m^1^A levels on tRF-3s in tumors, and that the elevation of TRMT6/61A in BLCA is associated with the expected induction of tRF-3 targets in tumors.

### tRF-3b modification by TRMT6/61A is important for maintaining the unfolded protein response

**Fig. 8:**
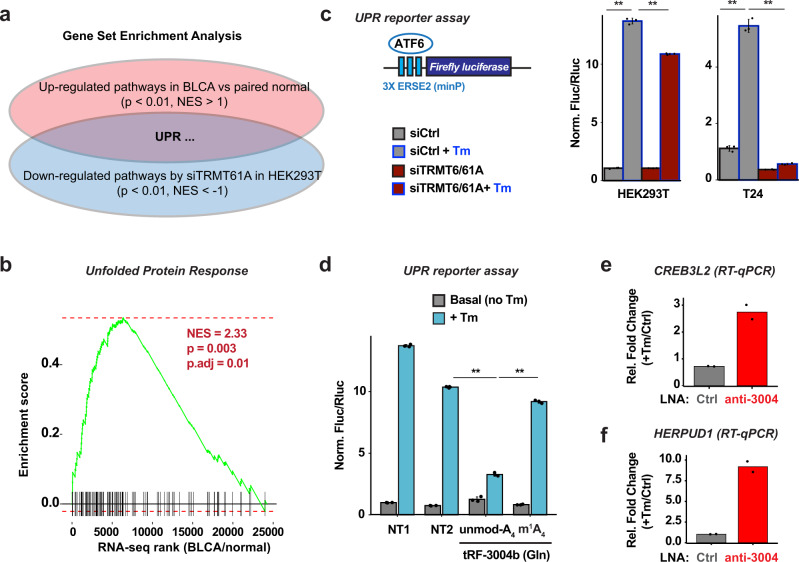
tRF-3b modification by TRMT6/61A is important for maintaining the unfolded protein response. **a** GSEA from RNA-seq in BLCA and HEK293T siTRMT61A data reveals Unfolded Protein Response (UPR) as a candidate pathway regulated by TRMT6/61A. **b** Gene Set Enrichment Analysis shows UPR as positively enriched pathway in BLCA tumor compared to the paired normal samples (*n* = 5, *x*-axis: ranking based on differential analysis from upregulated genes to downregulated genes). **c** TRMT6/61A regulates UPR response by UPR reporter assay in HEK293T and the BLCA tumor cell line T24. Three tandem repeats of ATF6 response element ERSE2 is inserted in the Firefly luciferase gene promoter. UPR response is measured by Firefly luciferase signals divided by Renilla luciferase signals (on co-transfected plasmid) and normalized to siCtrl basal level. Tunicamycin (Tm) is added to trigger UPR response. **d** m^1^A status on tRF-3 regulates UPR reporter output. tRF-3004b synthetic mimic and UPR reporter plasmid (same as above) are co-transfected into HEK293T. NT1 and NT2 are non-targeting control RNAs. **e**, **f** tRF-3004 targets have increased levels after tRF-3004 knock-down and tunicamycin treatment in HEK293T cells. Data are based on **c**, **d** *n* = 3 independent experiments, **e**, **f** *n* = 2 independent experiments. **c**, **d** Data represent mean ± SD, *p* value based on student’s t test (two-tailed paired sample, *p < 0.05, **p < 0.01). Exact *p* values from left to right: **c** 0.00019, 0.00409, 0.00088, and 0.00062; **d** 0.00013, 0.00066. See also Supplementary Fig. 7. Source data are provided as a Source Data file.

To explore the potential biological functions of m^1^A-dependent tRF-3 targets, we focused on biological pathways that are both significantly downregulated by siTRMT6/61A and significantly upregulated in high-*TRMT6* tumor samples by Gene Set Enrichment Analysis of our RNA-seq data. Enrichment analysis against a total of 1532 Reactome pathways identified seven overlapping pathways in both cell line and patient data. One such pathway is the unfolded protein response or UPR (Fig. 8a, b and Supplementary Fig. 7g). UPR also shows up as an enriched pathway when comparing TCGA BLCA patients with high *TRMT6* expression versus ones with low *TRMT6* expression (Supplementary Fig. 7h).

m^1^A-dependent tRF-3 targets, particularly MBTPS1 (also known as site-1 protease or S1P) and CREB3L2, have previously been implicated in UPR and protein secretion^48,49^, suggesting TRMT6/61A might help to sustain UPR in growing cells by de-repressing these tRF-3 targets. MBTPS1 (S1P) is well known for its role to cleave and activate different protein substrates on the Golgi, including the transcription factors ATF6 and CREB3L2^50–52^. To directly test whether TRMT6/61A regulates UPR in response to ER stress, we utilized a luciferase reporter driven by minimal promoter with ER stress response element 2 (ERSE2) that can be activated by ATF6 transcription factor^53^. The reporter showed increased luciferase activity dependent on both the ER response element and the ER stress (tunicamycin, Tm) as expected. Such UPR induced stress response is significantly dampened by TRMT6/61A knockdown in both HEK293T and T24 bladder cancer cell lines (Fig. 8c). Transfection of unmodified tRF-3004b, but not m^1^A-modified tRF-3004b, can reduce UPR reporter activity relative to NT1 and NT2 non-targeting RNAs (Fig. 8d), suggesting the m^1^A status on tRF-3b can directly regulate unfolded protein response. To ensure that endogenous tRF-3004b represses the target genes involved in UPR, we knocked down endogenous tRF-3004b by LNA, and this significantly increased levels of target cellular genes CREB3L2, HERPUD1, MBTPS1, and HLTF after ER stress (Fig. 8e, f and Supplementary Fig. 7i). Altogether, this suggests TRMT6/61A-mediated m^1^A modification on tRF-3s is important for maintaining UPR homeostasis.

## Discussion

By systematic profiling and benchmarking with synthetic m^1^A RNAs (Fig. 1), we found in this study that m^1^A modification exists mostly on tRFs among the human small RNAs (Fig. 2). The m^1^A modification is highly specific and prevalent on both nuclear-encoded tRFs and mitochondria-encoded tRFs. m^1^A on tRF-3s from nuclear-encoded tRNAs is mediated by TRMT6/61A complex, and resides uniquely in the seed region of tRF-3s (Fig. 3). TRMT6/61A-dependent m^1^A negatively regulates gene-silencing activity of tRF-3s (Fig. 4) and mediates global gene expression via tRF-3 seed pairing (Fig. 6) without changes in Ago association (Fig. 5). Lastly, we found TRMT6/61A expression is upregulated in urothelial carcinoma of the bladder relative to the normal bladder lining, and this is accompanied by higher m^1^A levels on tRF-3s and upregulation of tRF-3 targets that are enriched in the unfolded protein response (Figs. 7 and 8). Consistent with this correlation, experimental down-regulation of TRMT6/61A and therefore decreased tRF-3 m^1^A levels leads to a reduced unfolded protein response (Fig. 8). Collectively these results describe a TRMT6/61A, tRF-3 mediated mechanism of gene regulation that is altered during malignant transformation.

To map m^1^A sites comprehensively, we combined antibody enrichment and TGIRT-seq mismatch analysis; such combination has been successfully applied to map low-stoichiometry m^1^A sites in mRNAs^36,39^. Antibody enrichment has been a gold standard for modification mapping due to easy implementation, but suffers from potential off-target effects from the antibody. For example, it was recently found that m^1^A antibody from MBL cross-reacts with m^7^G at the mRNA cap^44^. Therefore it is important to corroborate enrichment by different antibodies, and also consider other measurement such as mismatch analysis. We have taken both ways to improve the rigor: enrichment by two m^1^A antibodies was considered (Supplementary Fig. 2c) and TGIRT-induced mismatch (misincorporation) signature is also evaluated at base-resolution (Fig. 1c). Taken together, this shows a list of m^1^A sites in small RNAs, and highlights the enrichment of tRNA fragments among the m^1^A-modified short RNAs (Fig. 2 and Supplementary Table 2). Consistent with this, their mismatch pattern is responsive to TRMT6/61A knockdown, a known m^1^A methyltransferase. We avoided the template-switching RT reaction by TGIRT^54,55^ and instead used ligated adaptor to initiate RT in order to avoid cloning of RT-truncated product that will lead to ambiguous mapping for small RNAs. Recently another group found that a similar approach enabled better quantification of tRNAs by avoiding the 3′ end bias of TGIRT template-switching activity^12^. We did not incorporate demethylase treatment in our workflow, as initial optimization found demethylase treatment leads to more variability and RNA degradation, similar to what has been noted by others^39^. The side-by-side comparison of antibody enrichment with TGIRT-induced mismatch is important for several reasons: (a) Not all short RNAs showing mismatch in the TGIRT-seq were enriched by m^1^A RIP. This will help identify additional modifications in the short RNAs that cause mismatch and not just m^1^A. (b) Some other short RNAs, including several microRNAs and RNY4 Y-RNA fragment, were also consistently enriched by both m^1^A antibodies (Supplementary Table 2). However, since we could not find additional evidence (TGIRT mismatch signature or responsiveness to TRMT6/61A knock down), additional experiments will be needed to evaluate whether these are bona fide m^1^A-containing RNAs or due to cross-reactivity with the antibody. (c) While m^1^A RIP confirms TGIRT mismatch results, m^1^A RIP did not help us find newer short RNAs with modifications at low stoichiometry. Although high confidence m^1^A sites were focused on tRFs in this study, other m^1^A-containing small RNAs are very likely to emerge by more sensitive measurement or under specific biological conditions, especially various stress conditions that regulate m^1^A on mRNAs or tRNAs^14,36,37,40,41^.

It is important to note that m^1^A-containing RNAs are normally under-represented, as commonly used M-MuLV reverse transcriptases (e.g., ProtoScriptII and SuperScript) are prone to stall at the m^1^A sites due to interrupted base pairing. Applying TGIRT for small RNA-seq allows a more accurate representation of m^1^A-containing small RNAs (Supplementary Fig. 3d and 7d). In particular, ProtoScriptII under-clones m^1^A-containing tRF-3b and misleadingly shows higher abundance for tRF-3a than tRF-3b (Supplementary Fig. 8). However, other than tRF-3s from tRNA^Ala^ and tRNA^Tyr^ that have higher levels of tRF-3a, the other tRF-3s (from tRNA^Ser^, tRNA^Val^, tRNA^Leu^, tRNA^Gln^ and tRNA^Gly^) have more tRF-3b than tRF-3a by TGIRT-seq or ProtoScriptII after siTRMT6/61A. Although the synthetic m^1^A RNA tested in this study shows a linear relationship between m^1^A stoichiometry and TGIRT mismatch rate (Fig. 1c), this linear correlation could be affected by the sequence context. In the future, we hope to build a calibration curve for each specific sequence in order to deduce the absolute m^1^A stoichiometry on specific short RNAs under different biological conditions. Meanwhile, m^1^A mismatch rate can still be used as a semi-quantitative index for m^1^A stoichiometry, especially when comparing the same sequence between different conditions. RT-1306 (an engineered HIV-1 RT) also shows a good linear relationship between mismatch rate and m^1^A% for synthetic m^1^A-containing RNA (Fig. 1c), and is another promising RT that can be used for m^1^A-mapping in the future. In addition to m^1^A, other internal and terminal modifications also hinder the efficient cloning of various small RNAs by the conventional small RNA-seq method, representing a unique opportunity for better method development to inform future research. Very recently, PANDORA-seq is developed to tackle these modifications by combinatorial enzymatic treatment on small RNAs, exposing an underappreciated amount of non-canonical small RNAs^56^.

Emerging evidence shows that tRNA modifications can affect tRF biogenesis, as shown for 5-methylcytosine, pseudouridine, 1-methylguanosine, queuosine and 5′ methylphosphorylation^57–61^. Here we find that when m^1^A level is decreased by TRMT6/61A knockdown, overall levels of tRF-3s are not significantly altered (Fig. 3g, Supplementary Figs. 3d, e and 4c), but rather the m^1^A level on tRF-3s is decreased. Interestingly, the decrease of m^1^A on tRF-3s was observed on most tRF-3s except the one from tRNA^iMet^ (Figs. 3c and  5d). tRNA^iMet^ is a well-known substrate for *TRMT6/61A* homolog in yeast (*GDC10/GDC14*) and is uniquely destabilized and degraded in loss of TRMT6/61A^62^. It has been noted in human cells, tRNA^iMet^ remains highly m^1^A modified, even after the loss of TRMT6/61A, probably due to the degradation of the hypomodified tRNAs^63^. Furthermore, it has been noted that tRNA^Glu^ and tRNA^Asp^ have lower m^1^A modification at A58 position compared to other tRNAs^12,38,63^, and we observed very low levels of tRF-3s from tRNA^Glu^ and tRNA^Asp^, suggesting tRF-3 biogenesis may have some link to the T-loop modification. While biogenesis factors like endoribonuclease or exoribonuclease involved in generating tRF-3s remain to be identified, this report suggests tRF-3 modification levels could be altered under physiological conditions. It is known that TRMT6 and TRMT61A work together as heterodimers, with TRMT61A containing the catalytic activity and TRMT6 facilitating tRNA binding^64^. TRMT6/61A has been shown to methylate specific mRNAs and protein translation is attenuated for m^1^A-modified mRNAs^36^. To further examine whether the observed target RNA level changes (Fig. 6) could be confounded by an unknown mechanism due to the m^1^A status of the target RNAs, we compare the annotated m^1^A sites in HEK293T from two independent sources^36,39^ with our RNA-seq results (Supplementary Fig. 5g–h), pointing against this possibility. Our results identify a mechanism by which TRMT6/61A regulates gene expression via tRNA fragments.

It has been shown by NMR that m^1^A disrupts base pairing and leads to less stable RNA duplex^65^. The m^1^A modification at the fourth position is sufficient to disrupt tRF-3b gene-silencing activity (Figs. 4 and  6), which is consistent with the fact that seed pairing is essential for RISC-mediated gene silencing^18,45^. In particular, sub-seed base pairing between positions 2 and 4 of the guide RNA and the target RNA is critical to initiate and extend the seed pairing, and Ago2 binding to target RNA is more affected by mismatches in positions 2–4 than positions 5–6^66^. It will be interesting to investigate whether Ago1/3/4 are also sensitive to m^1^A in the seed region, and whether m^1^A plays similar regulatory functions of other m^1^A-containing small RNAs, for example on the mitochondria tRFs where m^1^A is located on the 9^th^ position (Supplementary Fig. 3c). Likewise, how m^1^A modification may regulate other tRF functions through base pairing is worth future study, such as ribosome biogenesis regulation by tRF-3b base pairing with a ribosome protein mRNA^33^.

The changes in gene expression seen specifically with mRNA targets that can pair with the tRF-3 seed are due to changes in the modifying enzyme, and do not require overexpression of the tRNA. This adds further support to the hypotheses that short RNAs like tRF-3s can enter into functional complexes with AGO at endogenous cellular concentrations and silence target genes by base-pairing with the seed using mechanisms similar to microRNAs. To the best of our knowledge, this is the first example of microRNA-like gene silencing being regulated by the TRMT6/61 based m^1^A modification, and our report provides a mechanism by which the elevation of TRMT6/61A seen in cancers can impact gene expression. The fact that tRF-3s share very similar seed sequences and their target genes often have more than one seed-match sites suggest tRF-3s from different parental tRNAs may very likely act together via this mechanism.

Lastly, fast proliferating cancer cells maintain protein homeostasis via activating pro-survival UPR to alleviate ER stress^67^. As a result, UPR is tightly linked to many aspects of cancer hallmarks and has emerged as promising therapeutic target. It has been previously noted that UPR-related genes are globally upregulated in several cancer types including bladder cancer^68^. Here we found that TRMT6/61A promotes UPR most likely by modifying tRF-3s and de-repressing tRF-3 targets MBTPS1 and CREB3L2 in the ATF6 branch of UPR pathways. Intriguingly, MBTPS1 and CREB3L2 can be targeted by tRF-3004b from tRNA^Gln^, a tRNA that has been found to regulate UPR in yeast^69^. Of course, we do not rule out the possibility that TRMT6/61A could have an additional effect on UPR by modifying tRNA and mRNAs. How we can exploit the mechanism described here to sensitize tumor cells to pro-apoptosis UPR will be another interesting future topic of research.

## Methods

### Clinical samples from bladder cancer patients

All procedures and analyses were done in accordance with the study protocol approved by Vestre Viken Hospital Trust and The Regional Ethics Committee South-Eastern Norway Regional Health Authority (reference #2017/2170). Bladder tissue specimens were obtained from patients undergoing transurethral resection of bladder tumors (TURBT) at Vestre Viken Hospital Trust following written informed consent without participant compensation. Only patients with non-muscle invasive papillary urothelial carcinomas were included. Anonymized collective patient information of samples used is listed in Supplementary Table 6. From each patient, two samples were collected endoscopically prior to tumor resection by cold cup biopsy from tumor-free bladder lining followed by biopsy from the visualized tumor. The samples were immediately placed in RNAlater stabilization solution (Ambion #AM7020) according to the manufacturer’s instructions and kept on −20 °C until further processing.

### Cell culture

HEK293T (female), HeLa (female), and U251 (male) were maintained in HyClone Dulbecco’s High Glucose Modified Eagles medium with L-glutamine (GE #SH30081.01) plus 10% fetal bovine serum (Gibco #10437028) and 1% penicillin/streptomycin (Gibco #15140122). HEK293T was obtained from ATCC (ATCC #CRL-3216) and similarly for HeLa (ATCC #CCL-2). U251 was a kind gift from Roger Abounader. T24 bladder cancer cell line (female) was obtained from ATCC (ATCC #HTB-4) and maintained in MyCoy’s 5A medium with L-glutamine (Corning #10-050-CV) plus 10% FBS and 1% penicillin/streptomycin. Mycoplasma contamination was routinely checked by PCR kit (SouthernBiotech #13100-01). Cells were grown in humidified incubators with 5% CO_2_ at 37 °C.

To construct stable HEK293T cells expressing Flag-HA-Ago2, HEK293T cells were first co-transfected with pLJM1-Flag-HA-Ago2-WT, pMD2.G and psPAX2 to package lentivirus. 48 h after transfection, supernatant containing virus was harvested and used to transduce HEK293T cells. Puromycin selection was performed for 5 days. pLJM1-Flag-HA-Ago2-WT (Addgene plasmid #91978) was a gift from Joshua Mendell^70^. pMD2.G (Addgene plasmid #12259) and psPAX2 (Addgene plasmid #12260) were gifts from Didier Trono. The expression of tagged Ago2 was confirmed by western blotting against pan-Ago (Millipore #MABE56, clone 2A8) (Fig. 5a) and against FLAG (Sigma #F-1804) (Fig. 5c).

### RNA purification

*For cell lines:* Total RNAs was extracted from cell lines by TRIzol Reagent (Invitrogen #15596018) and Direct-zol RNA Miniprep Plus Kit with on-column DNase treatment (ZYMO #R2071).

*For patient samples:* Total RNA was extracted from patient samples using RNAzol RT (Molecular Research Center, Inc. #RN190) according to the manufacturer’s instructions. RNA quality was assessed using RNA ScreenTape on TapeStation (Agilent Technologies #5067-5576) or Agilent RNA 6000 Pico Kit on Bioanalyzer (Agilent Technologies #5067-1513).

### Synthetic m^1^A RNA oligonucleotides

Two m^1^A-containing RNA oligos were used to benchmark the m^1^A RIP or mismatch analysis by small RNA-seq (oligo sequences see Supplementary Table 7). Two oligo sequences (Control 1 and 2) were designed to avoid mapping on human genome. m^1^A-modified and unmodified RNA oligo pairs were synthesized with 5′ monophosphorylation and 3′ hydroxylation, followed by HPLC purification by IDT. To confirm the presence of m^1^A, 5 pmole of non-m^1^A and m^1^A-containing oligos were probed by anti-m^1^A immuno-Northern blot (Fig. 1c). To create a gradient of m^1^A% for testing different RTs (Fig. 1c), m^1^A- and unmodified-control1 oligos were mixed at different ratios. For each m^1^A ratio (0, 20, 40, 60, 80, and 100%), 2 pmole of total oligos were mixed with 1 μl QIAseq miRNA Library QC Spike-ins (Qiagen #331535) before small RNA-seq (refer to small RNA-seq library preparation). Four pairs of m^1^A-modified and unmodified RNA oligos with tRF-3b sequences were used for dual luciferase assay (oligo sequences in Supplementary Table 7). Four tRF-3b human sequences were from tRFdb database^71^.

### m^1^A RNA-immunoprecipitation (RIP)

m^1^A RIP was performed similarly as described previously for mRNA m^1^A RIP^36,38^, with adjustment to use small RNAs as input. To purify small RNAs <200 nt, PureLink miR isolation kit (Invitrogen #K157001) was used with 10^7^ starting cells or 50–100 μg total RNAs. Briefly, 1–5 μg purified small RNAs were immunoprecipitated with 5 μg anti-m^1^A antibody (MBL #D3453, or Abcam #ab208196) or corresponding IgG controls (mouse normal IgG – Millipore #12-371, or rabbit normal IgG – Millipore #12-370) conjugated on Protein G Plus Agarose (Pierce #22851) in the presence of SUPERase•In RNase Inhibitor (Invitrogen #AM2694) at 4 °C for 3 h. Post binding, resin was washed with 1X IPP buffer (10 mM Tris-HCl, pH 7.4, 150 mM NaCl, 0.1% NP-40) twice, low salt buffer (10 mM Tris-HCl, pH 7.4, 75 mM NaCl, 0.1% NP-40) once, high salt buffer (10 mM Tris-HCl, pH 7.4, 200 mM NaCl, 0.1% NP-40) once and TEN buffer (10 mM Tris-HCl, pH 7.4, 1 mM EDTA, 0.05% NP-40) twice. To elute bound RNAs, 3 mg/ml free 1-methyladenosine (Cayman Chemical #16937) in 1X IPP buffer was used to compete and displace m^1^A-containing RNAs at room temperature under rotation for 1 h with a total number of two elutions. The eluted RNAs were further purified by acidic phenol-chloroform method and dissolved in water. The efficiency of m^1^A RIP was monitored by relative fold enrichment (RIP over Input) of spiked in synthetic m^1^A oligos detected by small RNA-seq (Fig. 1e, Supplementary Fig. 1d, and Supplementary Table 1) normalized to total reads mapped to QIAseq QC Spike-ins. Synthetic m^1^A oligos and QIAseq QC Spike-ins were mixed with cellular RNAs before small RNA purification and RIP. Additionally, m^1^A RIP efficiency was examined for known m^1^A-containing tRNAs by SybrGold staining or Northern blot (Fig. 2a).

To identify m^1^A antibody enriched small RNAs in HEK293T cells, m^1^A RIP was performed by two m^1^A antibodies and sequenced with input and IgG control RNAs. A list of small RNAs that are significantly enriched by m^1^A RIP but not by control IgG RIP is shown in Supplementary Table 2 (DESeq2 adjusted *p* value < 0.1, at least 10 read counts to be considered for differential analysis). 77 of small RNAs (clustered on parental gene levels) are significantly enriched by both m^1^A antibodies.

### Small RNA-seq library preparation

Small RNA-seq library preparation was performed similarly as previously described^72,73^ using NEBNext Small RNA Library Prep Set for Illumina (NEB #7330) with the following modifications. Briefly, 0.1–1 μg total RNAs were ligated with 3′ adaptor and 5′ adaptor provided with NEBNext kit. In the case of purified small RNAs, RIP RNAs or synthetic m^1^A-containing RNAs, diluted adaptor amount (5% of the recommended amount by the kit) was used instead. Adaptor-ligated RNAs were converted to cDNAs by different reverse transcriptases as follows.

*(1) For TGIRT:* 1 μL TGIRT-III enzyme (InGex #TGIRT50) was used per cDNA synthesis reaction at 60 °C for 15 min. The reaction is then incubated with 250 mM (final concentration) NaOH at 95 °C for 3 min and 65 °C for 15 min to degrade RNAs. After reaction cools down, same amount of HCl was used to neutralize the reaction and the whole reaction is further purified by Dynabeads MyOne Silane (Thermo Fisher #37002D) or QIAquick Nucleotide Removal Kit (Qiagen #28304).

*(2) For ProtoScriptII (in NEBNext Kit):* 1 μL enzyme was used per reaction at 50 °C for 1 h and heat inactivated at 70 °C for 15 min; no purification was performed before PCR amplification.

*(3) For RT-1306 (a kind gift from Bryan Dickinson):* 1 μL of 10 μM recombinantly expressed and purified enzyme^39^ was used per reaction. Other conditions are the same as ProtoScriptII.

All cDNA synthesis was carried out in buffer (50 mM Tris, pH 8.3, 75 mM KCl, 3 mM MgCl2, 1 mM dNTP, 10 mM DTT) plus 1 μL RNase Inhibitor and followed by 12–16 cycles of PCR amplification with indexed primers (NEB #E6609). PCR cycle number is optimized per batch of reactions by testing different cycle numbers with small amount of cDNA and checked on gel. The resulting amplified libraries were individually purified by ZYMO DNA Clean & Concentrator Kit (ZYMO #D4033) and size selected on 8% TBE polyacrylamide Novex gel (Thermo Fisher #EC6215) to enrich for insert of 15–50 nucleotides long (longer than primer dimer and shorter than full-length tRNA). Final libraries were pooled and sequenced on Illumina NextSeq500, according to validated standard operating procedures established by the University of Virginia Genome And Technology Core, RRID: SCR_018883.

### Small RNA-seq mapping and mismatch analysis

Small RNA-seq data were analyzed similarly as previously described^72,73^. Briefly, cutadapt v1.15^74^ was used to quality trim with cut-off 20 (with nextseq trim option), trim 3′ adaptor sequence, and discard trimmed read length shorter than 15 nt. Due to higher frequency of cloning 5′ adaptor for TGIRT-seq libraries, reads containing 5′ adaptor sequence were discarded with cutadapt; this is to avoid mis-annotation of 5′ adaptor sequence to hsa-miR-3168. In general, each library has 2–10 million mapped reads. To quantify small RNAs, unitas v1.7.3^75^ with SeqMap v1.0.13^76^ was used to map the reads first to human sequence of miRBase Release 22^77^, then to other small RNA sequences including genomic tRNA database^78^, Ensembl Release 97 and SILVA rRNA database Release 132^79^. Unless otherwise specified (for example when testing 0 mismatch allowed in Fig. 2c), unitas setting (-species_miR_only –species homo_sapiens) was used to allow 2 non-templated 3′ nucleotides and 1 internal mismatch for miRNA mapping, and 1 mismatch and 0 insertion/deletion for tRNA fragments and other small RNA mapping (equivalent to –tail 2 –intmod 1 –mismatch 1 –insdel 0). microRNA reads were grouped by mature microRNA names, tRF raeds were grouped by parental tRNAs and tRF types (cut-off for tRNA halves is 30-nucleotide long); in the case of multi-mapping, a read was counted as fraction distributed equally to avoid duplicate counts.

*For mapping to synthetic spike-in RNAs*: 1 mismatch and 0 insertion/deletion were allowed. Mismatch percentage at the known m^1^A site was calculated based on read count with mismatch (C/G/T) at the given site divided by total read count for the given sequence (refer to Supplementary Table 1). To compare relative cloning frequency for 100% m^1^A Control1 RNA by different RTs (Fig. 1d), read count mapped to Control1 sequence was normalized to total read count mapped to QIAseq QC Spike-ins. Background mismatch% for different RTs was calculated similarly based on unmodified Control1 RNA (Supplementary Fig. 1c and Supplementary Table 1). Sequence logo was generated by ggseqlogo^80^ based on cloning frequency (Fig. 1c and Supplementary Fig. 1a, b).

*For calculating mismatch index on tRFs:* after mapping as above, reads mapped to a specific tRF or tRNA are counted and summed for mismatch at each position. Mismatch index (on a scale of 0–100%) is calculated for each position: mismatch index = reads that have mismatch at that position/total reads. Only tRF/tRNA with more than 50 reads is considered for mismatch index calculation.

### Knock down of TRMT6/61 A

To knock down TRMT6/61A in cell lines, 10 nM final total concentration of siRNA (Dharmacon ON-TARGETplus, TRMT6: #L-017324-02-0005, TRMT61A: #L-015870-01-0005) was transfected twice with Lipofectamine RNAiMax (Thermo Fisher #13778150) and collected 96 h post first transfection (48 h post second transfection). Non-targeting control siRNA (Dharmacon #D-001810-10-20) was used as negative control. Knock-down efficiency was confirmed by both RT-qPCR (Fig. 3d and Supplementary Fig. 3b, primer sequence in Supplementary Table 7) and western blot (Fig. 3d and Supplementary Fig. 3a) by TRMT6 mouse monoclonal antibody (SCBT #sc-271752, used at 1:1000 dilution). β-actin was probed by mouse monoclonal antibody (SCBT #sc-47778, used at 1:2000 dilution) as loading control. Western blots were performed with whole cell lysates on 12% SDS-PAGE and transferred to 0.45 μm nitrocellulose membrane (Whatman Protran BA85) by Trans-Blot Semi-Dry Transfer (Bio-Rad). Blocking and antibody incubation were in 3% milk in PBS with 0.05% Tween-20.

### Northern blot and m^1^A immuno-Northern blot

Northern blot was performed similarly as before^72,73^. Briefly, 1–5 μg purified total RNA or 5 pmole synthetic RNA oligos were resolved on 15% Novex TBE-Urea gel (Invitrogen #EC6885BOX) and transferred to Amersham Hybond-N + nylon membrane (Cytiva #RPN203B) by Trans-Blot SD semi-dry transfer apparatus (Bio-Rad). The membrane was cross-linked with 254 nm wavelength by Stratalinker (Strategene).

*For Northern blot:* After UV crosslinking, the membrane was blocked by ExpressHyb Hybridization Solution (Takara Bio #636833) and probed with biotinylated DNA probe (probe sequences see Supplementary Table 7) following the manufacturer’s instructions. Notably, the upper (U6 and full-length tRNA) and lower (tRF) parts of the membrane was cut after transfer, probed, and developed separately to avoid saturation of tRNA signals. Hybridized membrane was detected with Chemiluminescent Nucleic Acid Detection Module Kit (Thermo Fisher #89880).

*For immuno-Northern blot:* After UV crosslinking, the membrane was blocked with 3% milk in PBST and detected by m^1^A primary antibody (MBL #D3453, used at 1:2000 dilution) followed by anti-mouse HRP-linked secondary antibody (Cell Signaling #7076, used at 1:5000 dilution). Chemiluminescence detection was performed with Immobilon HRP substrate (Millipore #WBKLS0500). Oligo size was compared to microRNA marker (NEB #N2102S) on the gel.

### Dual luciferase reporter assay

Dual luciferase reporter was constructed in psiCHECK-2 vector (Promega #C8021) to insert target sequence into the 3′ UTR of Renilla luciferase gene. tRF-3021b^Ala^, tRF-3030b^Tyr^ and tRF-3004b^Gln^ reporters were constructed with single tRF-3b target sequence (reverse complement to tRF-3b sequence); tRF-3009b^Leu^ was previously constructed similarly^25^. Endogenous 3′ UTR sequences of *MBTPS1* (NM_003791.4, 3719-4296) and *CREB3L2* (NM_194071.4, 4531-6616) were PCR amplified by MyTaq HS Red Mix (Bioline #BIO-25047) from HEK293T genomic DNA extracted by QuickExtract DNA Extraction Solution (Epicenter #QE09050) and cloned into psiCHECK-2. 3′ UTR reporter. HEK293T was co-transfected 2 ng of luciferase plasmid and 100 nM tRF-3b mimic by Lipofectamine 2000 (Invitrogen #11668019) in 24-well plates; 24–48 h post transfection, cells were lysed and luciferase activity was measured by Dual-Luciferase Reporter 1000 Assay System (Promega #E1980). For each condition, Renilla luciferase value is normalized to Firefly luciferase value to account for well-to-well variation in cell density or transfection efficiency. In addition, empty psiCHECK-2 was included as a control for 3′ UTR reporter and used for normalization.

### Lock nucleic acid (LNA) to knock-down endogenous tRFs

LNA/DNA (synthesized by Qiagen or IDT) was used to knock-down endogenous tRF-3s (LNA sequences see Supplementary Table 7). For dual-luciferase reporter assay, 30 nM LNA was co-transfected with 2 ng of reporter plasmid by Lipofectamine 2000 (Invitrogen #11668019) in 24-well plates for 24–48 h. For qPCR experiment, 30 nM LNA was transfected with Lipofectamine RNAiMax (Thermo Fisher #13778150) for 24 h, and then treated with 5 μg/ml tunicamycin (Sigma #T7765) for 4 h before RNA collection.

### Real-time quantitative PCR (RT-qPCR)

*For cell lines:* RT-qPCR was performed to confirm knock-down efficiency and validate tRF target gene expression. Briefly, cDNA was generated from 1 to 2 μg DNase-treated total cellular RNA (refer to RNA isolation) by GoScript Reverse Transcriptase (Promega #A5004) with random primers (Invitrogen #48190011). RT-qPCR reactions were monitored on StepOnePlus Real-Time PCR System (Applied Biosystems) with PowerUp SYBR Green Master Mix (Applied Biosystems #A25741) and gene-specific primer sets to detect TRMT6/61A or tRF-3 targets (ACTB was used as internal control for normalization).

*For patient samples:* To examine mRNA expression level, 0.25–1 μg of total RNA (refer to RNA isolation) was used to synthesize complementary DNA (cDNA) using SuperScript IV VILO Master Mix with ezDNase Enzyme kit (Invitrogen #11766050). RT-qPCR was performed using PowerUp SYBR Green Master Mix and specific primers against targets. Relative mRNA levels were normalized to ACTB (chosen based on RNA-seq). All sequences of qPCR primers are listed in Supplementary Table 7.

### Ago2 RNA-immunoprecipitation (RIP)

Ago2 RIP was performed in stable FH-Ago2 HEK293T cells. Briefly, 10^7^ cells were collected by scraping and washed with DPBS, before lysis in RIP buffer (50 mM Tris-HCl, pH 7.4, 150 mM KCl, 0.5% NP-40 substitute, 0.5 mM DTT, 100X proteinase inhibitor, RNase inhibitor). Cleared lysates were incubated at 4 °C for 1 h with M2 FLAG affinity gel (Sigma #A2220) or anti-MYC mouse antibody 9E10 conjugated on beads as a negative control. After washes, bound RNAs are eluted by Trizol extraction and ethanol precipitation. IP efficiency was confirmed by western blot (Fig. 5c). Both input and Ago2-bound fractions were subjected to TGIRT-seq (details see small RNA-seq library preparation and analysis). After small RNA-seq, differential analysis to identify small RNAs (input and Ago2-bound fractions separately) altered by siTRMT6/61A was performed by DESeq2^81^ on count matrix of tRFs and miRs (at least 10 read counts to be considered for differential analysis).

### Structural modeling of human Ago2 with m^1^A-containing guide RNA

Co-crystal structure of human Ago2 and single-stranded guide RNA was obtained from PDB ID: 5js1^46^. The corresponding nucleic acid base at 4th position of guide RNA was substituted manually using Coot v0.8.9.1^82^ “replacing residue” function. The replaced m^1^A (code 1MA) backbone was fitted into the map. The final modeled structure was visualized using MacPyMOL v1.7.0.3.

### RNA-seq library preparation and analysis

Total RNAs (0.1–1 μg) were poly-A selected by NEBNext poly(A) mRNA Magnetic Isolation Module (NEB #E7490) and followed by NEBNext UltraII Directional RNA Kit (NEB #7765). The resulting libraries were pooled and sequenced as paired-end reads on Illumina HiSeq2000 (Novogene) or NextSeq (UVA GATC core facility, RRID: SCR_018883) with >20 million reads per sample. Standard RNA-seq analysis workflow was used^83^: briefly, kallisto v0.46.1^84^ was used with paired-end and stranded mode to quantify reads to human GRCh38 Refseq Transcripts (from NCBI Human Genome Resources). Differential gene expression analysis was carried out by DESeq2^81^ after tximport to convert estimated counts from kallisto to gene-level analysis. DESeq2 results were visualized by EnhancedVolcano^85^ with lowest adjusted *p* value set to 10^−10^. Gene Set Enrichment Analysis (GSEA) was performed on DESeq2-ranked genes by fgsea^86^ with 1000 permutations on pathway annotation from Molecular Signatures Databases^87^. Adjusted *p* value is corrected for multiple hypothesis testing on all Reactome pathways.

### Prediction of tRF targets based on seed sequences

To predict tRF-3b targets, Ago2-bound tRF-3b sequences were first clustered by their seed sequences (1–8 nt) and 6 seeds were identified to cover >90% of Ago2-bound tRF-3b sequences (Fig. 6b). Among the 6 Ago2-bound tRF-3 seeds, 5 are responsive to siTRMT6/61A (Fig. 6c) and further used for target prediction. TargetScan^88^ Release 7.0 custom perl program was used to predict tRF targets based on seed sequence matches in 3′ UTR (provided as 84-way multiple sequence alignment). Four types of target sites were predicted according to common definition for microRNA targets^88^: 8mer-A1 sites (2–8 nt base pairing, with 5′ A); 7mer-m8 sites (2–8 nt base pairing), 7mer-A1 sites (2–7 nt base pairing, with 5′ A); 6mer sites (2–7 nt base pairing). In this order, target genes are counted only by the highest rank of target type (for example, if a gene is predicted to be both 8mer-A1 and 7mer-m8 sites, it will only be counted once as 8mer-A1 site). For CDF plots, only genes with 3′ UTR sequences and expression level higher than 100 normalized read count (by DESeq2) were considered (conclusions remained the same regardless of different expression cut-off tested). CDF plots were centered by the non-targets median log2 Fold Change value. *P* value is calculated by Kolmogorov–Smirnov test to compare overall distribution between each type of tRF-3 targets and the non-targets.

### Protein extraction and western blotting of BLCA patient samples

For protein extraction, ~5 mg of tissue was placed in Metal Bead Lysing Matrix S tubes (MP Biomeicals #12747227) to which 5 mL of RIPA Lysis and Extraction Buffer (Thermo Fisher #8990) and 50 μL Protease Inhibitor Cocktail (Merck Life Sciences #O8340-5ML) was added. The tubes were processed using a FastPrep-24™ Classic Instrument (MP Biomedicals #116004500) and left to rotate for 2 h at 4 °C. Samples were transferred to Eppendorf tubes and sonicated at 50% amplification and 0.8 cycles (Sonics & Materials INC, model VC601). Following centrifugation at 16,000 rcf for 20 min at 4 °C, supernatant was recovered, and protein concentration was estimated by Bradford Assay (BioRad #500-0006). For Western blot analysis, 40 μg lysate was mixed with 4X Bolt™ LDS Sample Buffer (Invitrogen #B0007), heated for 10 min at 70 °C, and separated on Bolt 12% Bis-Tris Plus gel (Invitrogen #NW00120BOX) for 1.5 h at 150 V. Proteins were transferred onto a PVDF membrane (Biorad #1704156) with a Trans-blot turbo system (Biorad). Following blocking of the membranes for 1 h in 5% milk in PBS with 0.05% Tween-20 (PBST), the primary antibodies and corresponding dilutions were used in 5% milk in 0.05% PBST: anti-β-actin (Abcam #ab8224, mouse monoclonal, used at 1:1000), anti-TRMT6 (Abcam #ab235321, rabbit polyclonal, used at 1:1000), and anti-TRMT61A (Biorbyt #orb411814, rabbit polyclonal, used at 1:500). Anti-mouse (BioNordica #PI-2000, used at 1:10,000) and anti-rabbit (GE Healthcare Life Sciences #NA934-100UL, used at 1:10,000) HRP-linked secondary antibodies were used for detection of the respective targets. Blots were developed using the SuperSignal West Dura Extended Duration Substrate (Thermo Fisher #34095) for β-actin and SuperSignal West Femto Maximum Sensitivity Substrate (Thermo Fisher #34095) for TRMT6 and TRMT61A on Biorad ChemiDoc XRS + System. Band signal intensities were obtained with ImageLab Software (v5.2.1) and used to determine relative target protein levels normalized to β-actin which were visualized with GraphPad Prism (v9.1.0).

### Analysis of The Cancer Genome Atlas (TCGA) patient data

GEPIA2^89^ was used to compare TRMT6/61A mRNA expression between tumor (TCGA) and normal (TCGA plus GTEX) samples across cancers. TRMT6 mRNA expression across BLCA patients from TCGA pan-cancer study^90^ was downloaded using cBioPortal^91,92^. Patients were stratified by the highest and lowest quartile of TRMT6 expression, and patient IDs extracted. High and low TRMT6 expressing patient IDs were then used as input for differential expression analysis using TCGAbiolinks v2.20.1^93^ with DESeq2^81^. DESeq2 output was used for CDF plot and GSEA analysis. Correlational analysis between TRMT6/61A expression and tRF-3 targets was performed by GEPIA2^89^.

### Unfolded protein response reporter assay

UPR reporter assay in HEK293T or T24 cells was performed with ATF6-dependent promoter reporter (pGL4.29-ERSE2-luc2P-Hygro, Addgene plasmid #101790; http://n2t.net/addgene:101790; RRID:Addgene_101790), a gift from Seiichi Oyadomari. pRL-TK Renilla Luciferase Control plasmid was co-transfected as an internal control. Cells were transfected with reporter plasmids for 24 h before the readout; to trigger UPR, cells were treated with 5 μg/ml tunicamycin (Sigma #T7765) for 4 h before the readout. Luciferase readout is performed similarly as above dual-luciferase reporter assays. UPR response is measured by Firefly luciferase signals divided by Renilla luciferase signals (on co-transfected plasmid) and normalized to basal level.

### Quantification and statistical analysis

Number of independent biological replicates and statistical details are described in the corresponding figure legends. Student’s t-test, Wilcoxon test, and Kolmogorov–Smirnov test were performed by R v4.0.5 (“t.test”, “wilcox.test” and “ks.test”). Differential analysis for small RNA-seq and RNA-seq was performed by DESeq2 (v1.30.1) Wald test with Benjamini–Hochberg adjustment for multiple hypothesis testing.

### Reporting summary

Further information on research design is available in the Nature Research Reporting Summary linked to this article.

## Supplementary information

Supplementary Information

Reporting Summary

## Data Availability

The raw sequencing data for small RNA-seq and RNA-seq data from the HEK293T, HeLa, and U251 established cell lines that were generated in this study has been deposited in the Gene Expression Omnibus (GEO) database under accession code GSE171040. The raw sequencing data for small RNA-seq and RNA-seq in BLCA patients are not publicly available due to European and national regulations regarding patient privacy but will be available upon request. Requests should be directed to Rune Ougland (runoug@vestreviken.no) who will forward the request to the Data Protection Officer and the Ethical Committee for legal- and ethical evaluation. The data will be available for 10 years after publication and if the requesting institution has implemented the European GDPR, or is able to sign the Standard Contractual Clauses for international transfers, the process will take <6 weeks. Otherwise, inter-institutional negotiation is necessary which may prolong the wait time. The co-crystal structure of human Ago2 and single-stranded guide RNA used in this study is available in the PDB database under accession code 5js1. TCGA BLCA analysis was based on GEPIA2 Expression Analysis. Source data are provided with this paper.

## Source data

Source Data

## References

[1] Harcourt EM, Kietrys AM, Kool ET (2017). Chemical and structural effects of base modifications in messenger RNA. Nature.

[2] Helm M, Motorin Y (2017). Detecting RNA modifications in the epitranscriptome: Predict and validate. Nat. Rev. Genet..

[3] Roundtree IA, Evans ME, Pan T, He C (2017). Dynamic RNA modifications in gene expression regulation. Cell.

[4] Barbieri I, Kouzarides T (2020). Role of RNA modifications in cancer. Nat. Rev. Cancer.

[5] Song J, Yi C (2019). Reading chemical modifications in the transcriptome. J. Mol. Biol..

[6] Wiener D, Schwartz S (2021). The epitranscriptome beyond m(6)A. Nat. Rev. Genet..

[7] Kimura S, Dedon PC, Waldor MK (2020). Comparative tRNA sequencing and RNA mass spectrometry for surveying tRNA modifications. Nat. Chem. Biol..

[8] Pinkard O, McFarland S, Sweet T, Coller J (2020). Quantitative tRNA-sequencing uncovers metazoan tissue-specific tRNA regulation. Nat. Commun..

[9] Clark WC, Evans ME, Dominissini D, Zheng G, Pan T (2016). tRNA base methylation identification and quantification via high-throughput sequencing. RNA.

[10] Zheng G (2015). Efficient and quantitative high-throughput tRNA sequencing. Nat. Methods.

[11] Cozen AE (2015). ARM-seq: AlkB-facilitated RNA methylation sequencing reveals a complex landscape of modified tRNA fragments. Nat. Methods.

[12] Behrens A, Rodschinka G, Nedialkova DD (2021). High-resolution quantitative profiling of tRNA abundance and modification status in eukaryotes by mim-tRNAseq. Mol. Cell.

[13] Suzuki T (2020). Complete chemical structures of human mitochondrial tRNAs. Nat. Commun..

[14] Schwartz MH (2018). Microbiome characterization by high-throughput transfer RNA sequencing and modification analysis. Nat. Commun..

[15] Kariko K (2008). Incorporation of pseudouridine into mRNA yields superior nonimmunogenic vector with increased translational capacity and biological stability. Mol. Ther..

[16] Corbett KS (2020). SARS-CoV-2 mRNA vaccine design enabled by prototype pathogen preparedness. Nature.

[17] Mulligan MJ (2020). Phase I/II study of COVID-19 RNA vaccine BNT162b1 in adults. Nature.

[18] Bartel DP (2018). Metazoan microRNAs. Cell.

[19] Kim YK, Heo I, Kim VN (2010). Modifications of small RNAs and their associated proteins. Cell.

[20] Pandolfini L (2019). METTL1 promotes let-7 microRNA processing via m7G methylation. Mol. Cell.

[21] Seok H (2020). Position-specific oxidation of miR-1 encodes cardiac hypertrophy. Nature.

[22] Su Z, Wilson B, Kumar P, Dutta A (2020). Noncanonical Roles of tRNAs: tRNA fragments and beyond. Annu Rev. Genet..

[23] Magee R, Rigoutsos I (2020). On the expanding roles of tRNA fragments in modulating cell behavior. Nucleic Acids Res..

[24] Kumar P, Anaya J, Mudunuri SB, Dutta A (2014). Meta-analysis of tRNA derived RNA fragments reveals that they are evolutionarily conserved and associate with AGO proteins to recognize specific RNA targets. BMC Biol..

[25] Kuscu C (2018). tRNA fragments (tRFs) guide Ago to regulate gene expression post-transcriptionally in a Dicer-independent manner. RNA.

[26] Maute RL (2013). tRNA-derived microRNA modulates proliferation and the DNA damage response and is down-regulated in B cell lymphoma. Proc. Natl Acad. Sci. USA.

[27] Pekarsky Y (2016). Dysregulation of a family of short noncoding RNAs, tsRNAs, in human cancer. Proc. Natl Acad. Sci. USA.

[28] Ren B, Wang X, Duan J, Ma J (2019). Rhizobial tRNA-derived small RNAs are signal molecules regulating plant nodulation. Science.

[29] Guan L, Karaiskos S, Grigoriev A (2020). Inferring targeting modes of Argonaute-loaded tRNA fragments. RNA Biol..

[30] Schorn AJ, Gutbrod MJ, LeBlanc C, Martienssen R (2017). LTR-retrotransposon control by tRNA-derived small RNAs. Cell.

[31] Chen Q (2016). Sperm tsRNAs contribute to intergenerational inheritance of an acquired metabolic disorder. Science.

[32] Sharma U (2016). Biogenesis and function of tRNA fragments during sperm maturation and fertilization in mammals. Science.

[33] Kim HK (2017). A transfer-RNA-derived small RNA regulates ribosome biogenesis. Nature.

[34] Lee YS, Shibata Y, Malhotra A, Dutta A (2009). A novel class of small RNAs: tRNA-derived RNA fragments (tRFs). Genes Dev..

[35] Krishna S (2019). Dynamic expression of tRNA-derived small RNAs define cellular states. EMBO Rep..

[36] Li X (2017). Base-resolution mapping reveals distinct m(1)A methylome in nuclear- and mitochondrial-encoded transcripts. Mol. Cell.

[37] Dominissini D (2016). The dynamic N(1)-methyladenosine methylome in eukaryotic messenger RNA. Nature.

[38] Safra M (2017). The m1A landscape on cytosolic and mitochondrial mRNA at single-base resolution. Nature.

[39] Zhou H (2019). Evolution of a reverse transcriptase to map N(1)-methyladenosine in human messenger RNA. Nat. Methods.

[40] Liu F (2016). ALKBH1-mediated tRNA demethylation regulates translation. Cell.

[41] Richter U (2018). RNA modification landscape of the human mitochondrial tRNA(Lys) regulates protein synthesis. Nat. Commun..

[42] Chen Z (2019). Transfer RNA demethylase ALKBH3 promotes cancer progression via induction of tRNA-derived small RNAs. Nucleic Acids Res..

[43] Wei J (2018). Differential m(6)A, m(6)Am, and m(1)A demethylation mediated by FTO in the cell nucleus and cytoplasm. Mol. Cell.

[44] Grozhik AV (2019). Antibody cross-reactivity accounts for widespread appearance of m(1)A in 5′UTRs. Nat. Commun..

[45] Sheu-Gruttadauria J, MacRae IJ (2017). Structural foundations of RNA silencing by argonaute. J. Mol. Biol..

[46] Schirle NT (2016). Structural analysis of human argonaute-2 bound to a modified siRNA guide. J. Am. Chem. Soc..

[47] Zhang Z (2018). Global analysis of tRNA and translation factor expression reveals a dynamic landscape of translational regulation in human cancers. Commun. Biol..

[48] Khetchoumian K (2019). Pituitary cell translation and secretory capacities are enhanced cell autonomously by the transcription factor Creb3l2. Nat. Commun..

[49] Kondo, Y. et al. Site-1 protease deficiency causes human skeletal dysplasia due to defective inter-organelle protein trafficking. *JCI Insight*10.1172/jci.insight.121596 (2018). 10.1172/jci.insight.121596 PMC6124414 30046013

[50] Kondo S (2012). Activation of OASIS family, ER stress transducers, is dependent on its stabilization. Cell Death Differ..

[51] Kondo S (2007). BBF2H7, a novel transmembrane bZIP transcription factor, is a new type of endoplasmic reticulum stress transducer. Mol. Cell Biol..

[52] Ye J (2000). ER stress induces cleavage of membrane-bound ATF6 by the same proteases that process SREBPs. Mol. Cell.

[53] Kitakaze, K. et al. Cell-based HTS identifies a chemical chaperone for preventing ER protein aggregation and proteotoxicity. *Elife*10.7554/eLife.43302 (2019). 10.7554/eLife.43302 PMC6922633 31843052

[54] Lentzsch AM, Yao J, Russell R, Lambowitz AM (2019). Template-switching mechanism of a group II intron-encoded reverse transcriptase and its implications for biological function and RNA-Seq. J. Biol. Chem..

[55] Xu H, Yao J, Wu DC, Lambowitz AM (2019). Improved TGIRT-seq methods for comprehensive transcriptome profiling with decreased adapter dimer formation and bias correction. Sci. Rep..

[56] Shi J (2021). PANDORA-seq expands the repertoire of regulatory small RNAs by overcoming RNA modifications. Nat. Cell Biol..

[57] Blanco S (2014). Aberrant methylation of tRNAs links cellular stress to neuro-developmental disorders. EMBO J..

[58] Guzzi N (2018). Pseudouridylation of tRNA-derived fragments steers translational control in stem cells. Cell.

[59] Cosentino C (2018). Pancreatic beta-cell tRNA hypomethylation and fragmentation link TRMT10A deficiency with diabetes. Nucleic Acids Res..

[60] Wang X (2018). Queuosine modification protects cognate tRNAs against ribonuclease cleavage. RNA.

[61] Reinsborough CW (2019). BCDIN3D regulates tRNAHis 3′ fragment processing. PLoS Genet..

[62] Kadaba S (2004). Nuclear surveillance and degradation of hypomodified initiator tRNAMet in S. cerevisiae. Genes Dev..

[63] Saikia M, Fu Y, Pavon-Eternod M, He C, Pan T (2010). Genome-wide analysis of N1-methyl-adenosine modification in human tRNAs. RNA.

[64] Finer-Moore J, Czudnochowski N, O’Connell JD, Wang AL, Stroud RM (2015). Crystal structure of the human tRNA m(1)A58 methyltransferase-tRNA(3)(Lys) complex: Refolding of substrate tRNA allows access to the methylation target. J. Mol. Biol..

[65] Zhou H (2016). m(1)A and m(1)G disrupt A-RNA structure through the intrinsic instability of Hoogsteen base pairs. Nat. Struct. Mol. Biol..

[66] Chandradoss SD, Schirle NT, Szczepaniak M, MacRae IJ, Joo C (2015). A dynamic search process underlies microRNA targeting. Cell.

[67] Urra H, Dufey E, Avril T, Chevet E, Hetz C (2016). Endoplasmic reticulum stress and the hallmarks of cancer. Trends Cancer.

[68] Robinson JL, Feizi A, Uhlen M, Nielsen J (2019). A systematic investigation of the malignant functions and diagnostic potential of the cancer secretome. Cell Rep..

[69] Bruch, A., Klassen, R. & Schaffrath, R. Unfolded protein response suppression in yeast by loss of tRNA modifications. *Genes*10.3390/genes9110516 (2018). 10.3390/genes9110516 PMC6275073 30360492

[70] Golden RJ (2017). An Argonaute phosphorylation cycle promotes microRNA-mediated silencing. Nature.

[71] Kumar P, Mudunuri SB, Anaya J, Dutta A (2015). tRFdb: A database for transfer RNA fragments. Nucleic Acids Res..

[72] Su Z (2020). tRNA-derived fragments and microRNAs in the maternal-fetal interface of a mouse maternal-immune-activation autism model. RNA Biol..

[73] Su Z, Kuscu C, Malik A, Shibata E, Dutta A (2019). Angiogenin generates specific stress-induced tRNA halves and is not involved in tRF-3-mediated gene silencing. J. Biol. Chem..

[74] Martin M (2011). Cutadapt removes adapter sequences from high-throughput sequencing reads. EMBnet J..

[75] Gebert D, Hewel C, Rosenkranz D (2017). unitas: the universal tool for annotation of small RNAs. BMC Genomics.

[76] Jiang H, Wong WH (2008). SeqMap: Mapping massive amount of oligonucleotides to the genome. Bioinformatics.

[77] Kozomara A, Birgaoanu M, Griffiths-Jones S (2019). miRBase: From microRNA sequences to function. Nucleic Acids Res..

[78] Chan PP, Lowe TM (2016). GtRNAdb 2.0: An expanded database of transfer RNA genes identified in complete and draft genomes. Nucleic Acids Res..

[79] Quast C (2013). The SILVA ribosomal RNA gene database project: Improved data processing and web-based tools. Nucleic Acids Res..

[80] Wagih O (2017). ggseqlogo: A versatile R package for drawing sequence logos. Bioinformatics.

[81] Love MI, Huber W, Anders S (2014). Moderated estimation of fold change and dispersion for RNA-seq data with DESeq2. Genome Biol..

[82] Emsley P, Lohkamp B, Scott WG, Cowtan K (2010). Features and development of Coot. Acta Crystallogr. D Biol. Crystallogr..

[83] Love MI, Anders S, Kim V, Huber W (2016). RNA-Seq workflow: Gene-level exploratory analysis and differential expression. F1000Research.

[84] Bray NL, Pimentel H, Melsted P, Pachter L (2016). Near-optimal probabilistic RNA-seq quantification. Nat. Biotechnol..

[85] Blighe, K., Rana, S. & Lewis, M. Enhanced Volcano: Publication-ready volcano plots with enhanced colouring and labeling. R package version 1.8.0 (2020).

[86] Korotkevich, G., Sukhov, V. & Sergushichev, A. Fast gene set enrichment analysis. Preprint at *bioRxiv*10.1101/060012 (2019).

[87] Liberzon A (2015). The Molecular Signatures Database (MSigDB) hallmark gene set collection. Cell Syst..

[88] Agarwal, V., Bell, G. W., Nam, J. W. & Bartel, D. P. Predicting effective microRNA target sites in mammalian mRNAs. *Elife*10.7554/eLife.05005 (2015). 10.7554/eLife.05005 PMC4532895 26267216

[89] Tang Z, Kang B, Li C, Chen T, Zhang Z (2019). GEPIA2: An enhanced web server for large-scale expression profiling and interactive analysis. Nucleic Acids Res..

[90] Ding L (2018). Perspective on oncogenic processes at the end of the beginning of cancer genomics. Cell.

[91] Cerami E (2012). The cBio cancer genomics portal: an open platform for exploring multidimensional cancer genomics data. Cancer Discov..

[92] Gao J (2013). Integrative analysis of complex cancer genomics and clinical profiles using the cBioPortal. Sci. Signal.

[93] Colaprico A (2016). TCGAbiolinks: An R/Bioconductor package for integrative analysis of TCGA data. Nucleic Acids Res..

