# Supplementary material for: TRMT6/61A-dependent base methylation of tRNA-derived fragments regulates gene-silencing activity and the unfolded protein response in bladder cancer
Source: Nat Commun. 2022 Apr 20;13:2165. doi: 10.1038/s41467-022-29790-8 (PMC9021294; doi:10.1038/s41467-022-29790-8)
Supplement: Supplementary file 1 — Supplementary Information [file 41467_2022_29790_MOESM1_ESM.pdf]

TRMT6/61A-dependent base methylation of tRNA-derived fragments regulates gene-silencing activity and the unfolded protein response in bladder cancer

Supplemental Information

Supplementary Figures

Supplementary Figure 1. Systematic mapping of m^1A sites in small RNA space (related to Figure 1).

Supplementary Figure 2. Specific tRNA-derived fragments are highly enriched for m^1A (related to Figure 2).

Supplementary Figure 3. m^1A on 22-nucleotides 3' tRNA fragments is dependent on TRMT6/61A (related to Figure 3).

Supplementary Figure 4. Argonaute association of tRF-3b is not decreased by TRMT6/61A-dependent m^1A (related to Figure 4 and 5).

Supplementary Figure 5. m^1A-dependent changes in global gene silencing by tRF-3b (related to Figure 6).

Supplementary Figure 6. Pan-cancer analysis of *TRMT6* and *TRMT61A* expression in TCGA (related to Figure 7).

Supplementary Figure 7. Alteration of tRF-3 m^1A levels and tRF-3 targets affects unfolded protein response in bladder cancer (related to Figure 7 and 8).

Supplementary Figure 8. Relative abundance of tRF-3a and -3b from different tRNAs.

Supplementary Tables

Supplementary Table 1. Mismatch and enrichment analysis of synthetic m^1A oligos – related to Fig. 1.

Supplementary Table 2. m^1A antibody enriched small RNAs in HEK293T (ProtoScriptII) - related to Fig. 2d.

Supplementary Table 3. Mismatch analysis for tRF-3bs in Input and Ago2-bound fractions by TGIRT-seq.

Supplementary Table 4. Seed clusters of Ago2-bound tRF-3bs and their mismatch analysis upon siTRMT6/61A – related to Fig. 5.

Supplementary Table 5. List of tRF-3b targets down-regulated by siTRMT61A – related to Fig. 6.

Supplementary Table 6. BLCA patients sequenced in this study – related to Fig. 7.

Supplementary Table 7. List of RNA/DNA oligonucleotides in this study.

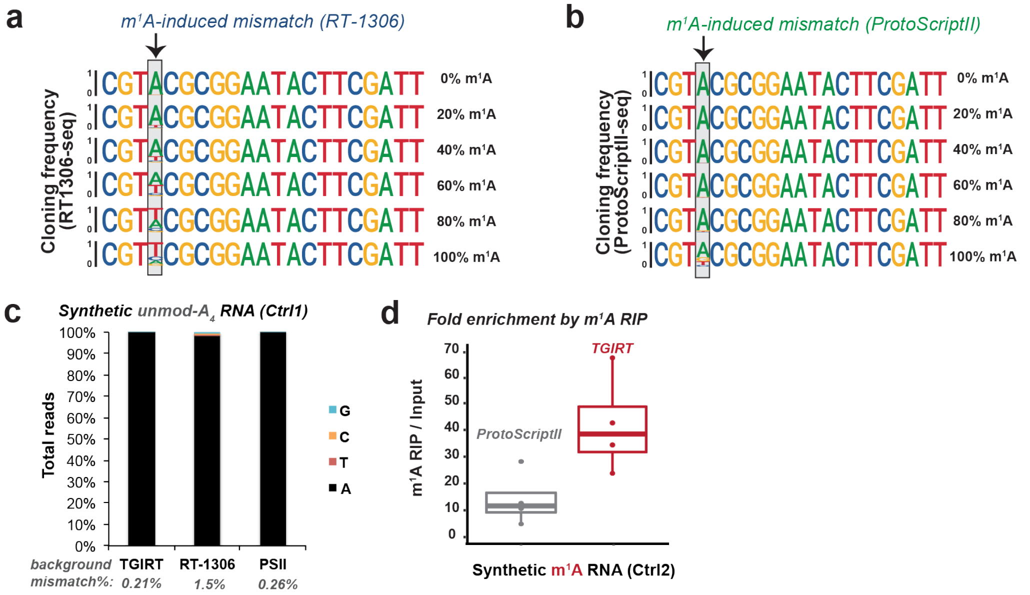

Supplementary Figure 1. Systematic mapping of m^{1}A sites in small RNA space (related to Figure 1).

(a-b) For each RT, mismatch rate was calculated across all reads that map to the synthetic RNA sequence (allowing 1 mismatch) as represented by the sequence logo. (c) Background mismatch rate is derived from A4 position of synthetic unmodified RNA sequenced by different reverse transcriptases. (d) Enrichment of synthetic m^{1}A-containing RNA by m^{1}A RIP (RNA immunoprecipitation) compared to input. Data are based on four independent RIP experiments (two HEK293T and two U251). Boxplot center represents median, bounds represent 25% and 75%, and whiskers show the minimum or maximum no further than 1.5 * interquartile range from the bound.

Source data are provided as a Source Data file.

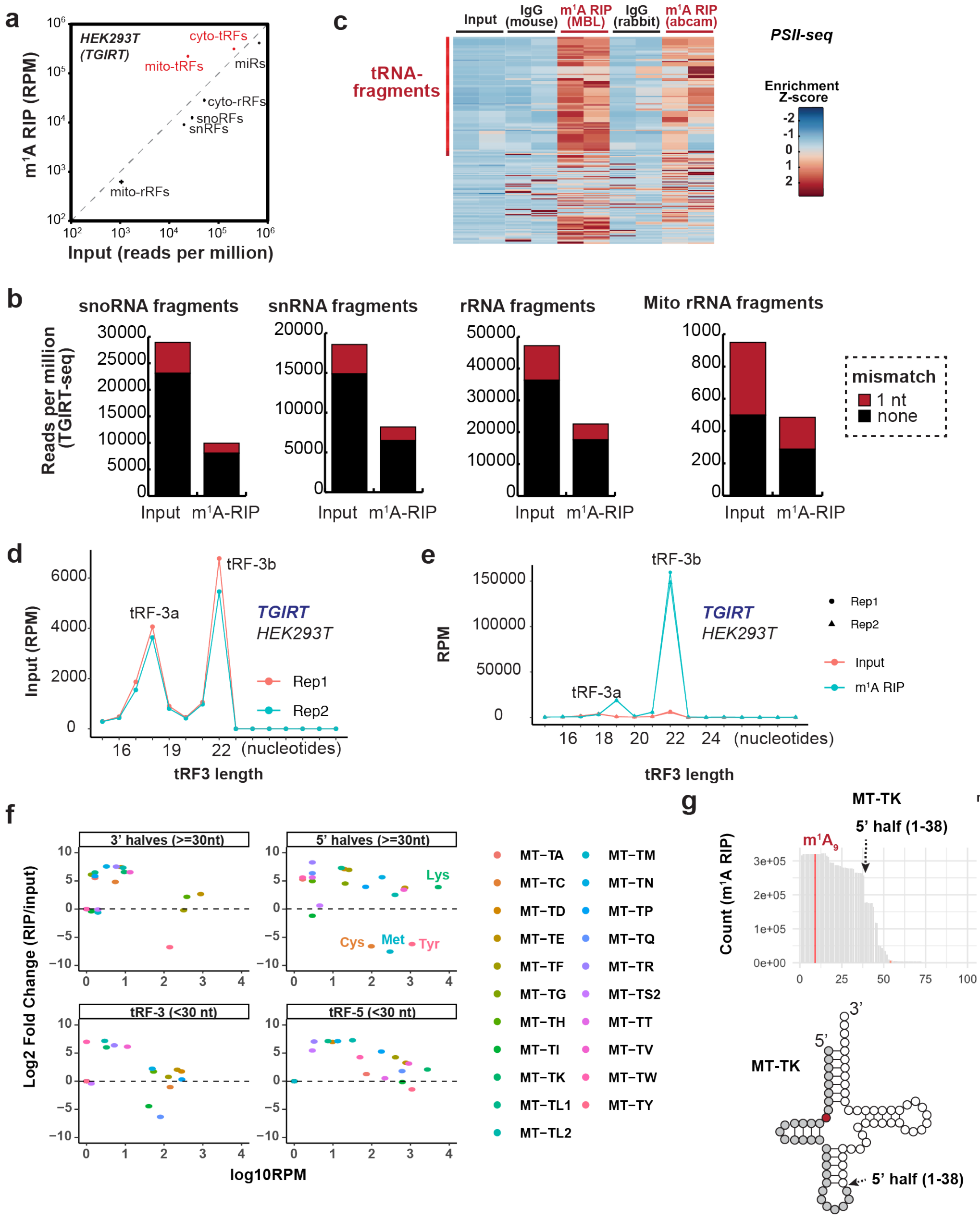

Supplementary Figure 2. Specific tRNA-derived fragments are highly enriched for m^{1}A (related to Figure 2).

(a) Scatter plot shows mean RPM (reads per million mapped reads) values on log scale for each group of small RNAs from m^{1}A RIP (RNA immunoprecipitation) compared to input small RNAs. (b) Bar graph represents RPM values for each group of small RNAs allowing no mismatch (black) and additional mapped reads when allowing 1-nt mismatch (red). (c) Heatmap of small RNAs that are significantly enriched (DESeq2 adjusted p value < 0.1) by m^{1}A RIP but not by control IgG RIP by either m^{1}A antibody. See also Supplementary Table 1. (d-e) Length distribution of tRF-3s in input (d) and m^{1}A RIP (e) by TGIRT-seq shows tRF-3b is significantly enriched by m^{1}A RIP. (f) MA plot shows 5' tRNA halves and tRF-5s are the most enriched among different mitochondrial tRF types. (g) Example coverage plot showing m^{1}A-specific mismatch locates at the 9th position of 5' half of Mito-tRNA^{Lys}. Mismatch rate is calculated for each position.

Data in this figure are based on TGIRT-seq (except c based on ProtoScriptII-seq) of two independent RIP experiments in HEK293T. Source Data are provided as a Source Data file.

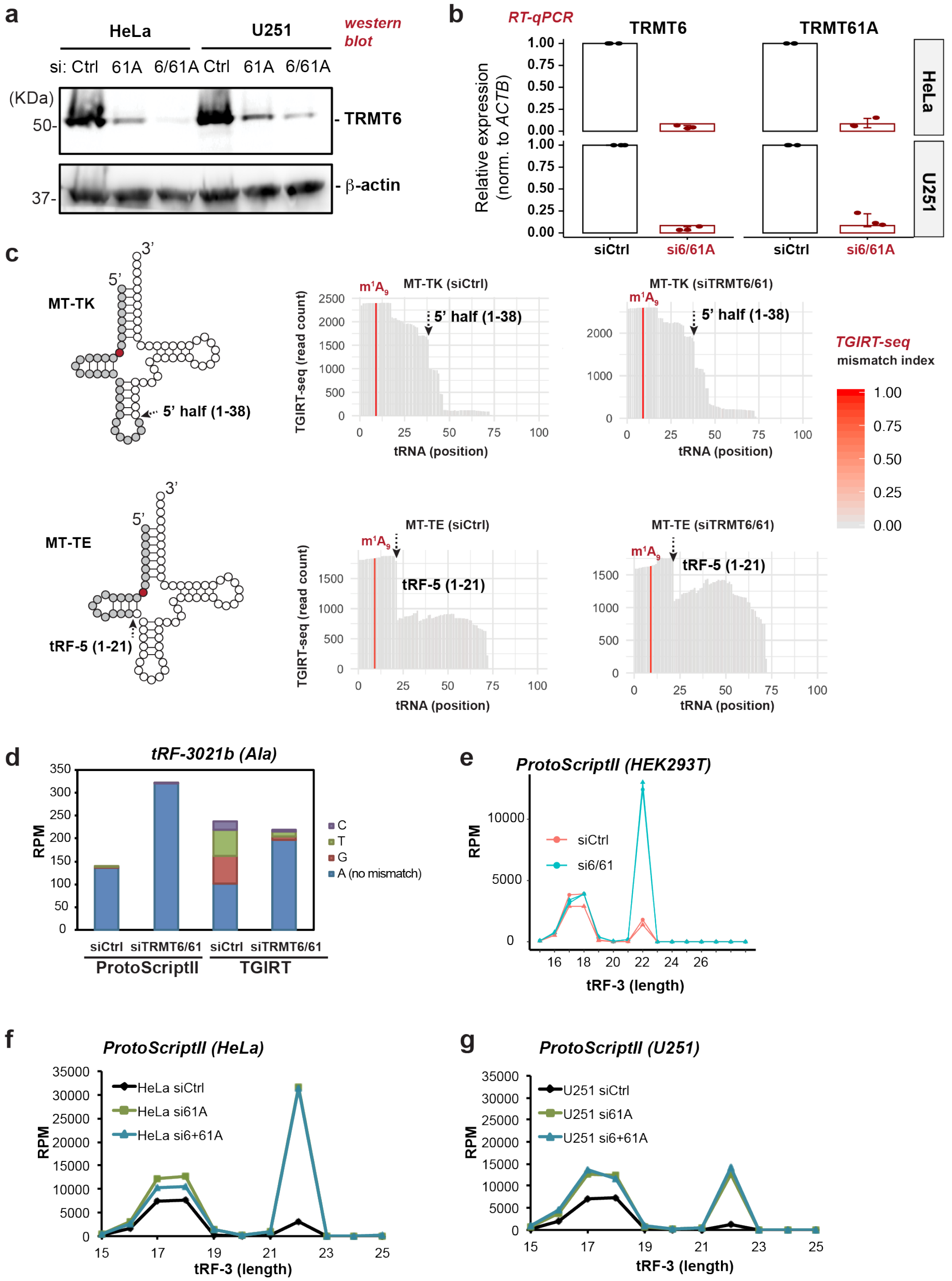

Supplementary Figure 3. m^{1}A on 22-nucleotides 3' tRNA fragments is dependent on TRMT6/61A (related to Figure 3).
(a-b) Knockdown of TRMT6/61A in HeLa and U251, confirmed by western blots (a) and RT-qPCR (b). RT-qPCR data was shown as mean ± SD (p value by two-tailed paired student's t test from three independent experiments). (c) m^{1}A on mitochondrial tRFs are not regulated by TRMT6/61A. Coverage plot showing m^{1}A mismatch at the 9th position of example tRFs. Mismatch rate is calculated for each position based on TGIRT-seq. (d-g) Small RNA cloning by ProtoScriptII leads to under-representation of m^{1}A-containing tRF-3b, which is alleviated by siTRMT6/61A.
Source data are provided as a Source Data file.

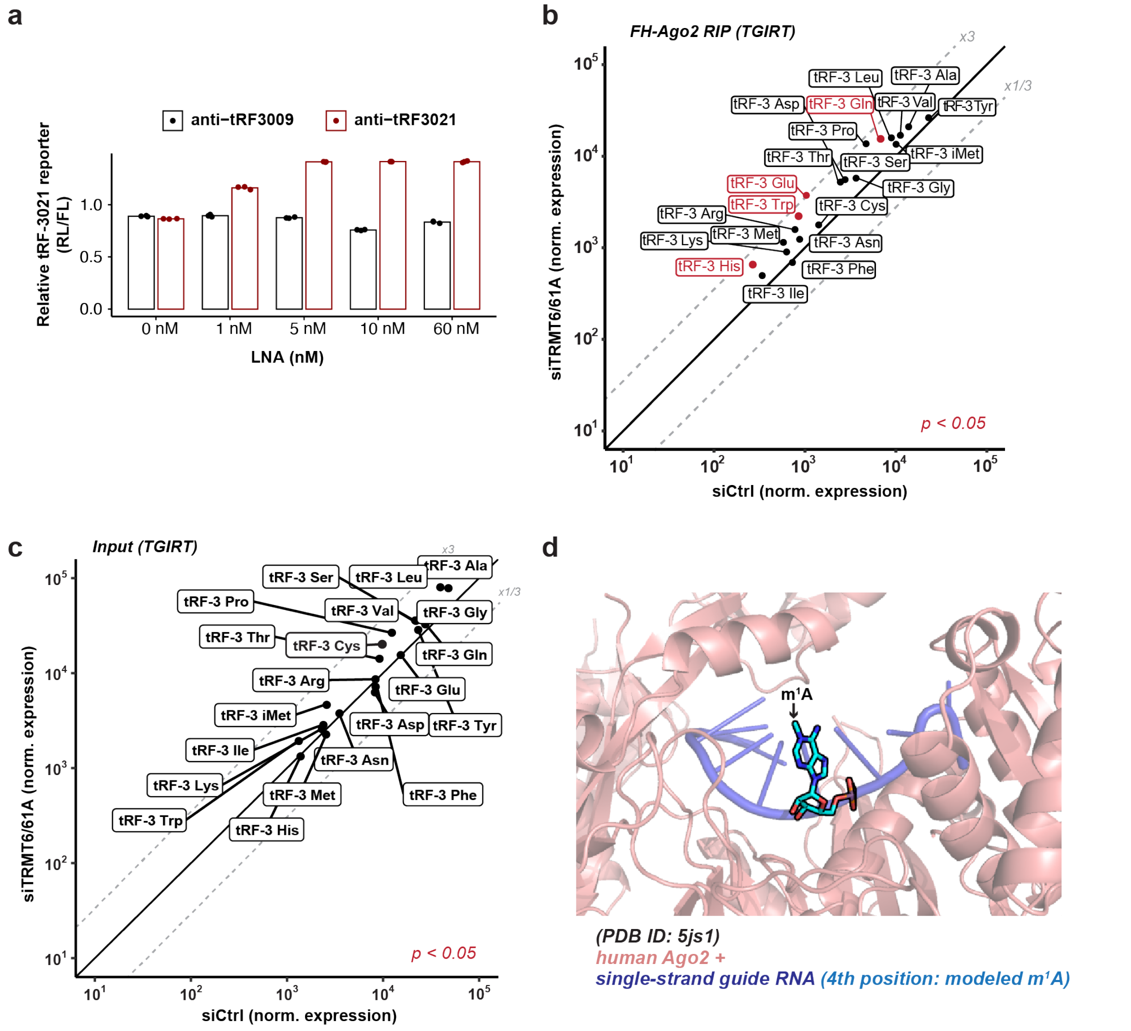

Supplementary Figure 4. Argonaute association of tRF-3b is not decreased by TRMT6/61A-dependent m^{1}A (related to Figure 4 and 5).

(a) De-repression of tRF-3021 reporter by LNA knock-down. LNA was co-transfected with dual-luciferase reporter plasmid in HEK293T. LNAs against tRF-3021 or tRF-3009 were titrated with control LNAs to maintain a constant total concentration of LNA. Relative activity is calculated from Rluc signal divided by Fluc signal, and normalized to the empty site reporter. Data are represented as mean ± SD (n = 3). (b-c) Differential analysis of input tRF-3s (b) and Ago2-bound tRF-3s (c) by TGIRT-seq (HEK293T, n = 3). Red dots show tRF-3s that are significantly different by TRMT6/61A knock-down ($p < 0.05$ by DESeq2 wald test). In both plots, no points passed DESeq2 adjusted $p < 0.1$. (d) Structural modeling of m^{1}A at the fourth position of single-stranded guide RNA in co-crystal structure of human Ago2 (PDB ID: 5js1). m^{1}A is modeled by mutating the original 4th position U on the guide RNA. The modeled structure suggests m^{1}A at 4th position of guide RNA does directly interacts with Ago2 protein.

Source data are provided as a Source Data file.

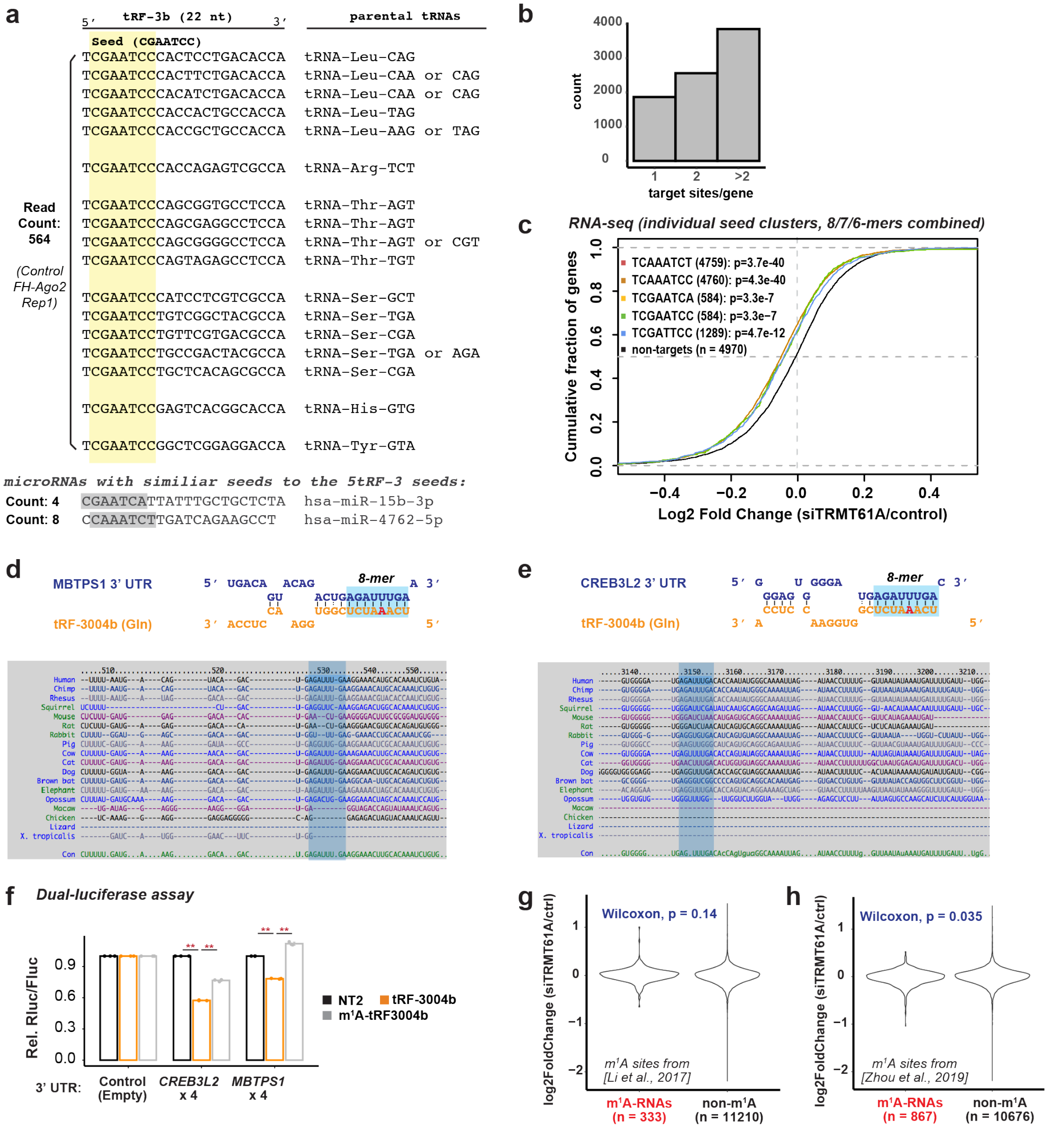

Supplementary Figure 5. m^1A-dependent changes in global gene silencing by tRF-3b (related to Figure 6).

(a) tRF-3b sequences can be clustered by their seed sequences, which are unique to tRFs (do not overlap with expressed microRNAs). Sequence alignments are shown for potential tRF-3b derived from different parental tRNA sequences, that share the same seed sequence (CGAATCC, 2-7 nt). Shown at the lower panel are microRNAs that have potential overlapping seed sequence with the 5 tRF-3b seeds (Fig. 6B), however these microRNAs are expressed at extremely low levels. (b) Most tRF-3 target genes have more than one tRF-3 target sites. (c) tRF-3 targets are globally repressed in RNA-seq compared to the non-targets upon TRMT61A knock-down in HEK293T. The trend holds true when considering individual seeds. Distribution of expression changes (Log2 scale on X axis from RNA-seq) is visualized by CDF (Cumulative Distribution Function) plot. P value is calculated by one-sided Kolmogorov-Smirnov test to compare overall distribution between each target type versus non-targets. Related to Fig. 6d. (d-e) Screenshots of TargetScan predicted tRF-3 sites in conserved 3' UTR region for *MBTPS1* (d) and *CREB3L2* (e). Both are predicted 8-mer matches by tRF-3004b from $tRNA^{Gln}$. (f) Dual-luciferase assay was performed to measure the effect after tRF-3004b mimic over-expression.

tRF-3004b target sites from endogenous *MBTPS1* and *CREB3L2* 3' UTR were cloned as 4X tandem repeats. Data are represented as mean ± SD from independent experiments (n = 3); the significance was based on two-tailed unpaired student's t test (*$p < 0.05$, **$p < 0.01$, N.S. = $p > 0.05$). Exact p values from left to right: 2.3e-5, 0.00023, 9.3e-5, 0.00043. (g-h) Gene expression changes by siTRMT61A are not explained by known m^1A-annotated RNAs (p value from two-sided Wilcoxon test between Log2 Fold Change of annotated m^1A-RNAs and that of other RNAs). Source data are provided as a Source Data file.

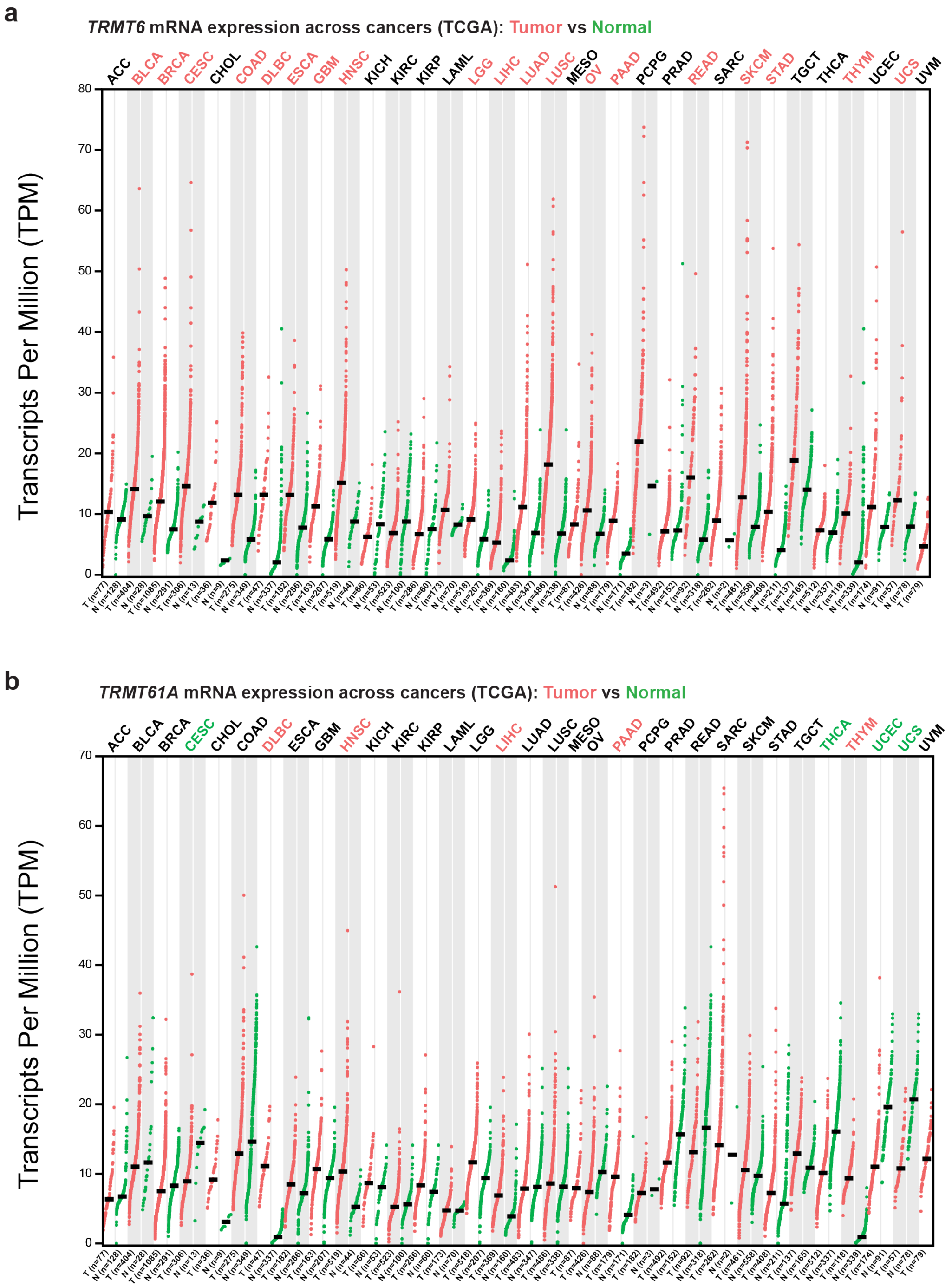

Supplementary Figure 6. Pan-cancer analysis of *TRMT6* and *TRMT61A* expression in TCGA (related to Figure 7).

Tumor (red dots) versus normal (green dots, including both TCGA normal and GTEX normal) were plotted from RNA-seq across different TCGA cancer types by GEPIA2 (a: *TRMT6*, b: *TRMT61A*). Cancer types with statistically significant difference in expression are colored in salmon – high in tumor, or green – low in tumor (cut-off: Log2FoldChange of median expression > 0.5, q < 0.05 by one-way ANOVA).

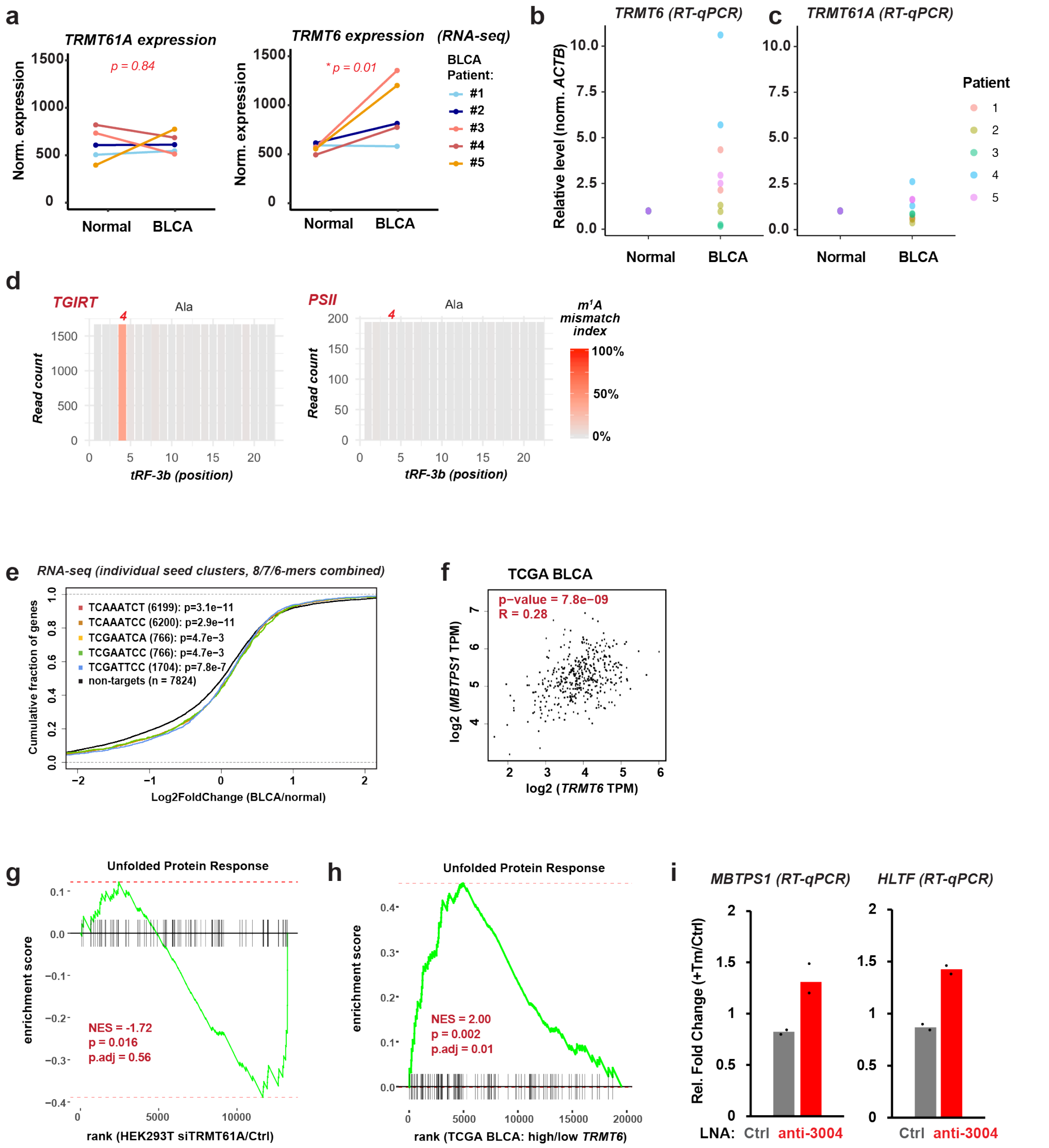

Supplementary Figure 7. Alteration of tRF-3 m^{1}A levels and tRF-3 targets affects unfolded protein response in bladder cancer (related to Figure 7).

(a) *TRMT6* RNA expression is significantly increased in BLCA tumor samples compared to the paired normal (n = 5) as measured by RNA-seq. For RNA-seq, normalized expression and p value is derived from Wald test by DESeq2. (b-c) *TRMT6* and *TRMT61A* RNA expression in BLCA tumor samples compared to the paired normal for five patients by RT-qPCR. *TRMT6* expression in each tumor is normalized to *ACTB* and further normalized to the paired normal control, each patient was measured in two independent experiments (shown as mean from three technical replicates in each experiment). (d) TGIRT-seq but not with ProtoScriptII (PSII) successfully detects m^{1}A by mismatch (indicated by red shade) on tRF-3b from BLCA patient samples. (e) Global de-repression of tRF-3b targets compared to non-targets observed in BLCA tumor samples compared to the paired normal (n = 5), when using individual seeds to predict targets. Distribution of expression changes (Log2 scale on X axis from RNA-seq) is visualized by CDF (Cumulative Distribution Function) plot. P value is calculated by one-sided Kolmogorov-

Smirnov test to compare overall distribution between each target type versus non-targets. (f) Positive correlation between *TRMT6* and *MBTPS1* expression in TCGA BLCA patients (n = 404). Pearson correlation was based on mRNA expression levels in TPM (transcripts per million) by GEPIA2 (visualized on log2 scale). Correlation coefficient and p value from paired correlation test. (g) UPR as negatively enriched pathway in HEK293T siTRMT61A compared to the siControl samples (x axis: ranking based on DESeq2 differential analysis from up-regulated genes to down-regulated genes). (h) UPR as positively enriched pathway in TCGA BLCA high-*TRMT6* (n = 102) compared to low-*TRMT6* (n = 102) samples. (i) tRF-3004 targets have increased levels after tRF-3004 knock-down and tunicamycin treatment in HEK293T cells. Data are normalized to *ACTB* and control LNA (lock nucleic acid) in basal condition, represented from two independent experiments.
Source data are provided as a Source Data file.

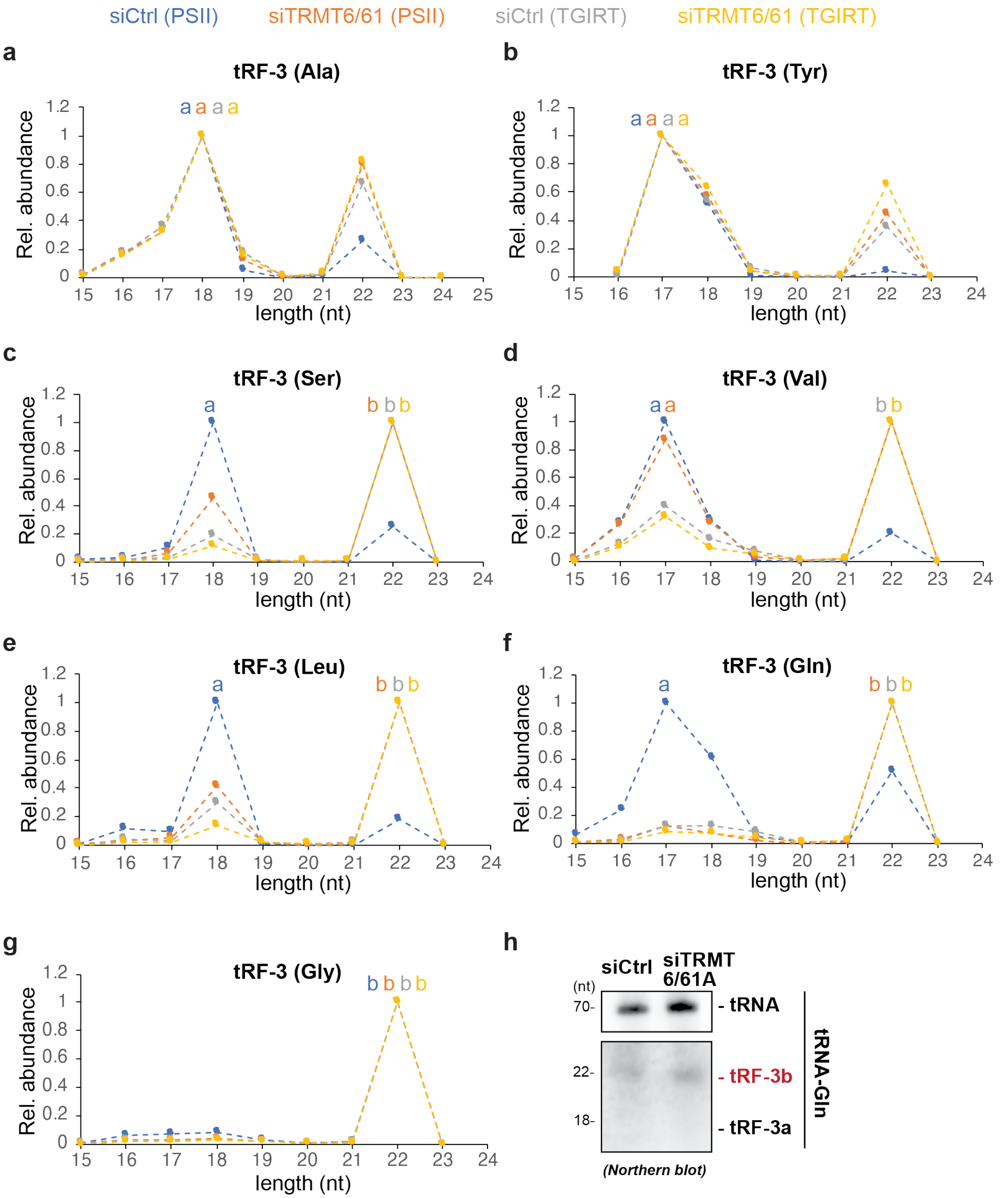

Supplementary Figure 8. Relative abundance of tRF-3a and -3b from different tRNAs.
(a-g) Length distribution of tRF-3s by sequencing. Four conditions are plotted (siCtrl by ProtoScriptII, siTRMT6/61A by ProtoScriptII, siCtrl by TGIRT and siTRMT6/61A by TGIRT). For each condition, relative abundance is normalized to the most abundant isoform (length). The most abundant isoform (length) is labeled for each condition. (h) Northern blot for $tRNA^{Gln}$ and its tRF-3b. tRF-3b level is unchanged by TRMT6/61A knock down.
Source data are provided as a Source Data file.

Supplementary Table 1. Mismatch and enrichment analysis of synthetic m1A oligos - related to Fig.1

	**Syn. Ctrl1 oligo:**	**0% m1A**	**20% m1A**	**40% m1A**	**60% m1A**	**80% m1A**	**100% m1A**
**TGIRT**	ReadCount (A4 - WT)	76967	63285	257147	11564	15467	5684
	ReadCount (T4)	37	18988	143665	16177	41430	74290
	ReadCount (C4)	22	2262	16356	1750	5199	8852
	ReadCount (G4)	104	2229	16578	1849	4424	8012
	**mismatch% at m1A site**	**0.21**	**27.06**	**40.71**	**63.10**	**76.75**	**94.13**
	ReadCount_spikeins	30	34	168	32	53	65
	Norm. Cloning Frequency (A4)	2565.57	1861.32	1530.64	361.38	291.83	87.45
	Norm. Cloning Frequency (T4)	1.23	558.47	855.15	505.53	781.70	1142.92
	Norm. Cloning Frequency (C4)	0.73	66.53	97.36	54.69	98.09	136.18
	Norm. Cloning Frequency (G4)	3.47	65.56	98.68	57.78	83.47	123.26
**RT-1306**	ReadCount (A4 - WT)	1538	2792	3861	1720	433	219
	ReadCount (T4)	8	302	951	1109	591	939
	ReadCount (C4)	5	84	261	275	146	254
	ReadCount (G4)	11	52	145	151	69	128
	**mismatch% at m1A site**	**1.54**	**13.56**	**26.01**	**47.16**	**65.05**	**85.78**
	ReadCount_spikeins	1	5	7	7	2	5
	Norm. Cloning Frequency (A4)	1538.00	558.40	551.57	245.71	216.50	43.80
	Norm. Cloning Frequency (T4)	8.00	60.40	135.86	158.43	295.50	187.80
	Norm. Cloning Frequency (C4)	5.00	16.80	37.29	39.29	73.00	50.80
	Norm. Cloning Frequency (G4)	11.00	10.40	20.71	21.57	34.50	25.60
**ProtoScriptII**	ReadCount (A4 - WT)	7733	2798	3089	1666	535	132
	ReadCount (T4)	8	9	15	18	14	31
	ReadCount (C4)	2	3	17	10	6	13
	ReadCount (G4)	10	7	13	21	22	36
	**mismatch% at m1A site**	**0.26**	**0.67**	**1.44**	**2.86**	**7.28**	**37.74**
	ReadCount_spikeins	78	16	26	61	33	45
	Norm. Cloning Frequency (A4)	99.14	174.88	118.81	27.31	16.21	2.93
	Norm. Cloning Frequency (T4)	0.10	0.56	0.58	0.30	0.42	0.69
	Norm. Cloning Frequency (C4)	0.03	0.19	0.65	0.16	0.18	0.29
	Norm. Cloning Frequency (G4)	0.13	0.44	0.50	0.34	0.67	0.80

	m1ACtrl1 (ReadCount Input)	m1ACtrl2 (ReadCount Input)	Spikein (ReadCount Input)	m1ACtrl1 (ReadCount m1ARIP)	m1ACtrl2 (ReadCount m1ARIP)	Spikein (ReadCount m1ARIP)	**m1ACtrl1 Relative_Fold_Enrichment (m1A RIP/Input)**	**m1ACtrl2 Relative_Fold_Enrichment (m1A RIP/Input)**
	**ProtoScriptII**							
HEK293T	65	5	8385	9102	396	23547	49.86	28.20
HEK293T	118	33	15014	3030	187	17461	22.08	4.87
U251	51	25	13755	1769	301	13095	36.43	12.65
U251	139	49	22498	2685	393	16951	25.64	10.64
	**TGIRT**							
HEK293T	865	158	2689	352	22	12	91.19	31.20
HEK293T	198	119	1304	658	60	32	135.42	20.55
U251	98	60	1036	178	37	10	188.17	63.89
U251	67	35	607	288	66	29	89.97	39.47

Supplementary Table 2. m1A antibody enriched small RNAs in HEK293T (ProtoScriptII)

Mapped Parental Gene (15-50nt sized RNA sequenced)	baseMean	MBL m1A antibody (mouse)					Abcam m1A antibody (rabbit)					Enriched by both antibodies
		LFC_m1A RIPvsinput	padj_m1A RIPvsinput	LFC_IgGvsinput	padj_IgGvsinput	p.adj<0.1 (LFC>0)	LFC_m1A RIPabcam vsinput	padj_m1A RIPabcam vsinput	LFC_IgGvsinput	padj_IgGvsinput	p.adj<0.1 (LFC>0)	
tRNA-Ala-TGC-1-1	1508.976	3.8824142	7.10E-12	0.814163	0.26059	m1A-enriched	3.172556	3.97E-08	1.028919	0.143434	m1A-enriched	Yes
tRNA-Ala-TGC-6-1	1496.388	3.9027486	1.42E-09	0.905133	0.264997	m1A-enriched	3.231322	1.01E-06	0.862638	0.296872	m1A-enriched	Yes
tRNA-Cys-GCA-10-1	462.4904	3.5825683	0.0003293	-0.639381	0.638974	m1A-enriched	3.160173	0.001995	-1.535849	0.226525	m1A-enriched	Yes
tRNA-Cys-GCA-13-1	457.9352	3.753743	0.0001429	-0.448031	0.768433	m1A-enriched	3.357797	0.000882	-1.344576	0.303717	m1A-enriched	Yes
tRNA-Cys-GCA-14-1	562.0059	2.9131889	3.84E-12	0.045702	0.960491	m1A-enriched	2.476738	6.13E-09	-0.382587	0.56108	m1A-enriched	Yes
tRNA-Cys-GCA-15-1	457.9201	3.7422864	0.0002459	-0.299784	0.851522	m1A-enriched	3.334923	0.00142	-1.354265	0.312672	m1A-enriched	Yes
tRNA-Cys-GCA-17-1	486.7467	4.5283019	1.05E-09	0.607965	0.560173	m1A-enriched	3.964615	1.54E-07	-5.744601	0.002605	m1A-enriched	Yes
tRNA-Cys-GCA-19-1	314.1786	3.2363367	6.20E-10	-0.794083	0.303456	m1A-enriched	2.869822	6.94E-08	-6.210127	0.000467	m1A-enriched	Yes
tRNA-Cys-GCA-20-1	404.1217	4.5922757	8.39E-20	0.329983	0.701529	m1A-enriched	4.196884	1.22E-16	-5.441521	0.00271	m1A-enriched	Yes
tRNA-Cys-GCA-22-1	401.2186	4.5964187	9.68E-20	0.242147	0.796173	m1A-enriched	4.185627	1.84E-16	-5.436129	0.002726	m1A-enriched	Yes
tRNA-Cys-GCA-8-1	564.3459	2.9219867	8.75E-12	0.044157	0.962966	m1A-enriched	2.47826	1.19E-08	-0.384155	0.563668	m1A-enriched	Yes
tRNA-Cys-GCA-9-1	464.6699	3.5807124	0.0005145	-0.48345	0.750818	m1A-enriched	3.16131	0.00277	-1.537846	0.23582	m1A-enriched	Yes
tRNA-Cys-GCA-9-2	464.6699	3.5807124	0.0005145	-0.48345	0.750818	m1A-enriched	3.16131	0.00277	-1.537846	0.23582	m1A-enriched	Yes
tRNA-Cys-GCA-9-3	464.6699	3.5807124	0.0005145	-0.48345	0.750818	m1A-enriched	3.16131	0.00277	-1.537846	0.23582	m1A-enriched	Yes
tRNA-Cys-GCA-9-4	464.6699	3.5807124	0.0005145	-0.48345	0.750818	m1A-enriched	3.16131	0.00277	-1.537846	0.23582	m1A-enriched	Yes
tRNA-Gly-CCC-1-1	3614.043	2.2399893	0.0008842	0.844166	0.274412	m1A-enriched	1.947085	0.004887	0.044219	0.978141	m1A-enriched	Yes
tRNA-Gly-CCC-1-2	3614.043	2.2399893	0.0008842	0.844166	0.274412	m1A-enriched	1.947085	0.004887	0.044219	0.978141	m1A-enriched	Yes
tRNA-Gly-GCC-1-1	3973.553	1.4332608	0.0710983	-0.610142	0.46605	m1A-enriched	1.388291	0.074773	-0.363396	0.688363	m1A-enriched	Yes
tRNA-Gly-GCC-1-2	3973.553	1.4332608	0.0710983	-0.610142	0.46605	m1A-enriched	1.388291	0.074773	-0.363396	0.688363	m1A-enriched	Yes
tRNA-Gly-GCC-1-3	3973.553	1.4332608	0.0710983	-0.610142	0.46605	m1A-enriched	1.388291	0.074773	-0.363396	0.688363	m1A-enriched	Yes
tRNA-Gly-GCC-1-4	3973.553	1.4332608	0.0710983	-0.610142	0.46605	m1A-enriched	1.388291	0.074773	-0.363396	0.688363	m1A-enriched	Yes
tRNA-Gly-GCC-1-5	3973.553	1.4332608	0.0710983	-0.610142	0.46605	m1A-enriched	1.388291	0.074773	-0.363396	0.688363	m1A-enriched	Yes
tRNA-Gly-GCC-2-1	3701.636	1.4947861	0.0106193	-0.587997	0.370419	m1A-enriched	1.102493	0.073308	-0.791478	0.209654	m1A-enriched	Yes
tRNA-Gly-GCC-2-2	3701.636	1.4947861	0.0106193	-0.587997	0.370419	m1A-enriched	1.102493	0.073308	-0.791478	0.209654	m1A-enriched	Yes
tRNA-Gly-GCC-2-3	3701.636	1.4947861	0.0106193	-0.587997	0.370419	m1A-enriched	1.102493	0.073308	-0.791478	0.209654	m1A-enriched	Yes
tRNA-Gly-GCC-2-4	3701.636	1.4947861	0.0106193	-0.587997	0.370419	m1A-enriched	1.102493	0.073308	-0.791478	0.209654	m1A-enriched	Yes
tRNA-Gly-GCC-2-5	3701.636	1.4947861	0.0106193	-0.587997	0.370419	m1A-enriched	1.102493	0.073308	-0.791478	0.209654	m1A-enriched	Yes
tRNA-Gly-GCC-2-6	3701.636	1.4947861	0.0106193	-0.587997	0.370419	m1A-enriched	1.102493	0.073308	-0.791478	0.209654	m1A-enriched	Yes
tRNA-Gly-GCC-5-1	2310.768	1.9618447	0.0108037	-0.40017	0.682581	m1A-enriched	2.021243	0.007356	0.027394	0.995369	m1A-enriched	Yes
tRNA-Gly-TCC-1-1	319.8283	3.4471818	0.0272078	1.082844	0.542875	m1A-enriched	3.346902	0.029789	-0.712934	0.722456	m1A-enriched	Yes
tRNA-Gly-TCC-3-1	348.0732	3.60333	0.000166	1.453493	0.19771	m1A-enriched	3.305384	0.000701	-0.659285	0.63261	m1A-enriched	Yes
tRNA-Leu-AAG-1-1	345.327	4.5799683	5.07E-05	1.948711	0.142603	m1A-enriched	3.172406	0.008815	1.848288	0.165965	m1A-enriched	Yes
tRNA-Leu-AAG-1-2	345.327	4.5799683	5.07E-05	1.948711	0.142603	m1A-enriched	3.172406	0.008815	1.848288	0.165965	m1A-enriched	Yes
tRNA-Leu-AAG-1-3	345.327	4.5799683	5.07E-05	1.948711	0.142603	m1A-enriched	3.172406	0.008815	1.848288	0.165965	m1A-enriched	Yes
tRNA-Pro-AGG-1-1	1242.532	1.9036955	0.0001118	0.055765	0.953771	m1A-enriched	1.329849	0.011716	0.443656	0.479953	m1A-enriched	Yes
tRNA-Pro-AGG-2-1	987.241	1.4983111	0.0231204	0.235928	0.79531	m1A-enriched	1.453053	0.025498	0.560998	0.461727	m1A-enriched	Yes
tRNA-Pro-AGG-2-2	987.241	1.4983111	0.0231204	0.235928	0.79531	m1A-enriched	1.453053	0.025498	0.560998	0.461727	m1A-enriched	Yes
tRNA-Pro-AGG-2-3	987.241	1.4983111	0.0231204	0.235928	0.79531	m1A-enriched	1.453053	0.025498	0.560998	0.461727	m1A-enriched	Yes
tRNA-Pro-AGG-2-4	987.241	1.4983111	0.0231204	0.235928	0.79531	m1A-enriched	1.453053	0.025498	0.560998	0.461727	m1A-enriched	Yes
tRNA-Pro-AGG-2-5	987.241	1.4983111	0.0231204	0.235928	0.79531	m1A-enriched	1.453053	0.025498	0.560998	0.461727	m1A-enriched	Yes
tRNA-Pro-AGG-2-6	987.241	1.4983111	0.0231204	0.235928	0.79531	m1A-enriched	1.453053	0.025498	0.560998	0.461727	m1A-enriched	Yes
tRNA-Pro-AGG-2-7	987.241	1.4983111	0.0231204	0.235928	0.79531	m1A-enriched	1.453053	0.025498	0.560998	0.461727	m1A-enriched	Yes
tRNA-Pro-AGG-2-8	987.241	1.4983111	0.0231204	0.235928	0.79531	m1A-enriched	1.453053	0.025498	0.560998	0.461727	m1A-enriched	Yes
tRNA-Pro-CGG-1-1	1000.402	1.3900573	0.0419736	0.106878	0.917335	m1A-enriched	1.308567	0.052519	0.417402	0.593785	m1A-enriched	Yes
tRNA-Pro-CGG-1-2	1000.402	1.3900573	0.0419736	0.106878	0.917335	m1A-enriched	1.308567	0.052519	0.417402	0.593785	m1A-enriched	Yes
tRNA-Pro-CGG-1-3	1000.402	1.3900573	0.0419736	0.106878	0.917335	m1A-enriched	1.308567	0.052519	0.417402	0.593785	m1A-enriched	Yes
tRNA-Pro-CGG-2-1	1003.04	1.4541074	0.0299894	0.074178	0.948168	m1A-enriched	1.31493	0.050967	0.404495	0.604684	m1A-enriched	Yes
tRNA-Pro-TGG-3-1	1085.685	1.6326703	0.0127562	0.187223	0.843644	m1A-enriched	1.387825	0.037661	0.555305	0.466317	m1A-enriched	Yes
tRNA-Pro-TGG-3-2	1085.685	1.6326703	0.0127562	0.187223	0.843644	m1A-enriched	1.387825	0.037661	0.555305	0.466317	m1A-enriched	Yes
tRNA-Pro-TGG-3-3	1085.685	1.6326703	0.0127562	0.187223	0.843644	m1A-enriched	1.387825	0.037661	0.555305	0.466317	m1A-enriched	Yes
tRNA-Pro-TGG-3-4	1085.685	1.6326703	0.0127562	0.187223	0.843644	m1A-enriched	1.387825	0.037661	0.555305	0.466317	m1A-enriched	Yes
tRNA-Pro-TGG-3-5	1085.685	1.6326703	0.0127562	0.187223	0.843644	m1A-enriched	1.387825	0.037661	0.555305	0.466317	m1A-enriched	Yes
tRNA-Ser-GCT-1-1	279.6254	2.8972288	0.0940157	1.943378	0.260155	m1A-enriched	2.992013	0.071828	2.166738	0.202668	m1A-enriched	Yes
tRNA-Ser-TGA-4-1	7578.204	2.8658449	0.0008	-1.518697	0.113629	m1A-enriched	2.500654	0.004407	-0.833964	0.421723	m1A-enriched	Yes
tRNA-Thr-TGT-3-1	8804.803	5.0837502	2.36E-11	0.517684	0.632341	m1A-enriched	3.607753	4.72E-06	0.157329	0.904537	m1A-enriched	Yes
MT-TK-ENSG00000210156.1	10632.94	6.2715956	0.0138946	0.550044	0.883755	m1A-enriched	8.048778	0.000769	-7.229754	0.018667	m1A-enriched	Yes
MT-TL1-ENSG00000209082.1	949.4302	5.7045682	0.0087375	-1.041987	0.721387	m1A-enriched	5.920784	0.005159	1.224107	0.640286	m1A-enriched	Yes
tRNA-leader_tRNA-Ser-TGA-3-1	96.13587	4.8267552	0.0051689	-3.538459	0.193649	m1A-enriched	5.120698	0.002404	-2.869931	0.296872	m1A-enriched	Yes
tRNA-leader_tRNA-Thr-CGT-4-1	41.54489	3.9978379	0.0103134	-3.74643	0.13979	m1A-enriched	2.813587	0.089057	-3.077902	0.224834	m1A-enriched	Yes
tRF-1_[2]_tRNA-Cys-GCA-2-3	4153.125	3.1563661	5.44E-05	1.33237	0.142603	m1A-enriched	1.71711	0.052318	0.856224	0.370907	m1A-enriched	Yes
tRF-1_tRNA-Lys-TTT-6-1	335.6193	7.3493921	3.63E-05	2.923165	0.165333	m1A-enriched	4.739269	0.014201	-2.867232	0.327447	m1A-enriched	Yes
RNY4_NR_004393.1	5915.555	3.9591609	6.40E-08	0.751376	0.423051	m1A-enriched	2.194851	0.007155	0.605811	0.538008	m1A-enriched	Yes
miR-1234-5p	45.98224	5.852449	0.0026166	2.216718	0.369572	m1A-enriched	4.976536	0.012827	3.221953	0.159125	m1A-enriched	Yes
miR-129-5p	1091.893	5.8098676	0.0162874	-0.551195	0.879586	m1A-enriched	4.714951	0.057	-5.985579	0.047761	m1A-enriched	Yes

miR-1306-3p	433.9509	3.8620754	0.0023435	2.201087	0.116122	m1A-enriched	2.670746	0.050967	2.196359	0.110317	m1A-enriched	Yes
miR-3147-5p	41.4982	4.9758279	0.0002046	-2.129054	0.410993	m1A-enriched	5.353595	4.80E-05	-1.460525	0.58855	m1A-enriched	Yes
miR-3187-3p	23.5469	4.5641802	0.0013195	-2.477752	0.341524	m1A-enriched	2.860146	0.068613	-0.376977	0.913135	m1A-enriched	Yes
miR-33b-5p	865.5187	6.20901	0.0047446	1.340565	0.628937	m1A-enriched	4.331756	0.064067	-0.525141	0.873079	m1A-enriched	Yes
miR-375-3p	12652.78	5.7337635	1.15E-09	0.769069	0.542153	m1A-enriched	4.994432	1.91E-07	-0.975644	0.439458	m1A-enriched	Yes
miR-4301-3p	9.897297	8.2870054	0.0127562	5.696471	0.128959	m1A-enriched	8.219871	0.012122	5.399035	0.173656	m1A-enriched	Yes
miR-4530-3p	20.66096	6.8954243	0.0575863	3.262415	0.42397	m1A-enriched	6.34564	0.07678	1.475317	0.762907	m1A-enriched	Yes
miR-4745-5p	50.32567	4.9957715	2.45E-06	-2.757653	0.239919	m1A-enriched	4.624845	1.50E-05	-2.089125	0.391218	m1A-enriched	Yes
miR-615-3p	2087.89	4.6193419	3.86E-08	-0.405136	0.757723	m1A-enriched	3.542167	5.21E-05	-3.797284	0.000557	m1A-enriched	Yes
miR-6730-5p	21.43066	8.403593	0.0005157	4.278323	0.164686	m1A-enriched	7.871805	0.001368	2.504155	0.487658	m1A-enriched	Yes
miR-6741-5p	16.49842	4.2609213	0.0111467	1.91034	0.417392	m1A-enriched	5.99389	9.53E-05	0.136199	0.982991	m1A-enriched	Yes
miR-6794-5p	10.07838	5.6010563	0.0924172	2.790536	0.453205	m1A-enriched	5.401351	0.096096	1.016333	0.828844	m1A-enriched	Yes
miR-7706-3p	4210.374	2.8194726	3.38E-12	-0.14823	0.825277	m1A-enriched	1.300116	0.004405	-0.629885	0.219119	m1A-enriched	Yes
tRNA-Ala-AGC-2-1	2132.876	1.2235978	0.07085	-0.839808	0.223505	m1A-enriched	0.557285	0.467059	-0.977319	0.14382	not significant	
tRNA-Ala-AGC-2-2	2132.876	1.2235978	0.07085	-0.839808	0.223505	m1A-enriched	0.557285	0.467059	-0.977319	0.14382	not significant	
tRNA-Ala-AGC-3-1	2132.864	1.2238195	0.07085	-0.839666	0.223505	m1A-enriched	0.557614	0.467059	-0.977176	0.14382	not significant	
tRNA-Ala-CGC-3-1	2723.102	4.3174766	5.80E-11	1.322058	0.102252	m1A-enriched	3.521887	1.91E-07	1.886874	0.012792	IgG-enriched	
tRNA-Ala-TGC-4-1	2744.303	4.1437652	6.02E-11	1.234323	0.113863	m1A-enriched	3.335405	2.97E-07	1.706695	0.019903	IgG-enriched	
tRNA-Gly-TCC-2-1	407.4044	3.1949602	4.43E-11	1.107723	0.074854	IgG-enriched	3.132753	1.58E-10	0.964565	0.139537	m1A-enriched	
tRNA-Gly-TCC-2-2	407.4044	3.1949602	4.43E-11	1.107723	0.074854	IgG-enriched	3.132753	1.58E-10	0.964565	0.139537	m1A-enriched	
tRNA-Gly-TCC-2-3	407.4044	3.1949602	4.43E-11	1.107723	0.074854	IgG-enriched	3.132753	1.58E-10	0.964565	0.139537	m1A-enriched	
tRNA-Gly-TCC-2-4	407.4044	3.1949602	4.43E-11	1.107723	0.074854	IgG-enriched	3.132753	1.58E-10	0.964565	0.139537	m1A-enriched	
tRNA-Gly-TCC-2-5	407.4044	3.1949602	4.43E-11	1.107723	0.074854	IgG-enriched	3.132753	1.58E-10	0.964565	0.139537	m1A-enriched	
tRNA-Gly-TCC-2-6	407.4044	3.1949602	4.43E-11	1.107723	0.074854	IgG-enriched	3.132753	1.58E-10	0.964565	0.139537	m1A-enriched	
tRNA-iMet-CAT-1-1	1567.226	1.7204162	0.0090312	-0.177567	0.850425	m1A-enriched	0.468218	0.573854	-0.453721	0.563668	not significant	
tRNA-iMet-CAT-1-2	1567.226	1.7204162	0.0090312	-0.177567	0.850425	m1A-enriched	0.468218	0.573854	-0.453721	0.563668	not significant	
tRNA-iMet-CAT-1-3	1567.226	1.7204162	0.0090312	-0.177567	0.850425	m1A-enriched	0.468218	0.573854	-0.453721	0.563668	not significant	
tRNA-iMet-CAT-1-4	1567.226	1.7204162	0.0090312	-0.177567	0.850425	m1A-enriched	0.468218	0.573854	-0.453721	0.563668	not significant	
tRNA-iMet-CAT-1-5	1567.226	1.7204162	0.0090312	-0.177567	0.850425	m1A-enriched	0.468218	0.573854	-0.453721	0.563668	not significant	
tRNA-iMet-CAT-1-6	1567.226	1.7204162	0.0090312	-0.177567	0.850425	m1A-enriched	0.468218	0.573854	-0.453721	0.563668	not significant	
tRNA-iMet-CAT-1-7	1567.226	1.7204162	0.0090312	-0.177567	0.850425	m1A-enriched	0.468218	0.573854	-0.453721	0.563668	not significant	
tRNA-iMet-CAT-1-8	1567.226	1.7204162	0.0090312	-0.177567	0.850425	m1A-enriched	0.468218	0.573854	-0.453721	0.563668	not significant	
tRNA-Leu-AAG-3-1	289.4558	5.3456468	0.0002432	0.916485	0.6417	m1A-enriched	3.689936	0.018522	3.456657	0.028092	IgG-enriched	
tRNA-Leu-CAG-1-1	1641.731	1.8128311	0.0975431	-0.185186	0.901898	m1A-enriched	0.471658	0.721313	0.224993	0.873079	not significant	
tRNA-Leu-CAG-1-2	1641.731	1.8128311	0.0975431	-0.185186	0.901898	m1A-enriched	0.471658	0.721313	0.224993	0.873079	not significant	
tRNA-Leu-CAG-1-3	1641.731	1.8128311	0.0975431	-0.185186	0.901898	m1A-enriched	0.471658	0.721313	0.224993	0.873079	not significant	
tRNA-Leu-CAG-1-4	1641.731	1.8128311	0.0975431	-0.185186	0.901898	m1A-enriched	0.471658	0.721313	0.224993	0.873079	not significant	
tRNA-Leu-CAG-1-5	1641.731	1.8128311	0.0975431	-0.185186	0.901898	m1A-enriched	0.471658	0.721313	0.224993	0.873079	not significant	
tRNA-Leu-CAG-1-6	1641.731	1.8128311	0.0975431	-0.185186	0.901898	m1A-enriched	0.471658	0.721313	0.224993	0.873079	not significant	
tRNA-Leu-CAG-1-7	1641.731	1.8128311	0.0975431	-0.185186	0.901898	m1A-enriched	0.471658	0.721313	0.224993	0.873079	not significant	
tRNA-Leu-TAG-1-1	344.0319	4.4289815	0.018483	1.794513	0.391136	m1A-enriched	3.02082	0.13219	1.197743	0.586876	not significant	
tRNA-Leu-TAG-2-1	160.7902	3.451919	0.0644782	1.581929	0.425289	m1A-enriched	2.330779	0.235239	3.046866	0.091076	not significant	
tRNA-Ser-AGA-1-1	1448.381	2.7386384	0.0594533	1.649875	0.264067	m1A-enriched	3.739248	0.004588	2.626062	0.056025	IgG-enriched	
tRNA-Ser-AGA-2-1	1442.829	2.747039	0.0575863	1.658451	0.26059	m1A-enriched	3.74888	0.004405	2.634636	0.054685	IgG-enriched	
tRNA-Ser-AGA-2-2	1442.829	2.747039	0.0575863	1.658451	0.26059	m1A-enriched	3.74888	0.004405	2.634636	0.054685	IgG-enriched	
tRNA-Ser-AGA-2-3	1442.829	2.747039	0.0575863	1.658451	0.26059	m1A-enriched	3.74888	0.004405	2.634636	0.054685	IgG-enriched	
tRNA-Ser-AGA-2-4	1442.829	2.747039	0.0575863	1.658451	0.26059	m1A-enriched	3.74888	0.004405	2.634636	0.054685	IgG-enriched	
tRNA-Ser-AGA-2-5	1442.829	2.747039	0.0575863	1.658451	0.26059	m1A-enriched	3.74888	0.004405	2.634636	0.054685	IgG-enriched	
tRNA-Ser-AGA-2-6	1442.829	2.747039	0.0575863	1.658451	0.26059	m1A-enriched	3.74888	0.004405	2.634636	0.054685	IgG-enriched	
tRNA-Ser-AGA-3-1	1448.244	2.7413163	0.0592609	1.652728	0.263708	m1A-enriched	3.741852	0.004571	2.628916	0.055875	IgG-enriched	
tRNA-Ser-AGA-4-1	1543.717	2.8209979	0.0420921	1.645301	0.254683	m1A-enriched	3.903827	0.002104	2.5833	0.054247	IgG-enriched	
tRNA-Ser-GCT-3-1	485.0518	2.8883843	0.0559224	1.745785	0.259874	m1A-enriched	2.854547	0.052103	2.560011	0.077185	IgG-enriched	
tRNA-Ser-TGA-2-1	1448.687	2.7411592	0.0591438	1.650532	0.264007	m1A-enriched	3.739906	0.004571	2.626719	0.055875	IgG-enriched	
tRNA-Ser-TGA-3-1	1443.209	2.746698	0.0575863	1.656247	0.26085	m1A-enriched	3.746676	0.004405	2.632431	0.0547	IgG-enriched	
tRNA-Thr-TGT-2-1	302.1537	2.2044073	0.0088851	-1.085281	0.26085	m1A-enriched	1.344751	0.14438	-0.019515	1	not significant	
tRNA-Thr-TGT-4-1	2027.005	2.5359503	0.0004878	0.678752	0.436445	m1A-enriched	1.356004	0.100481	0.167466	0.879904	not significant	
tRNA-Thr-TGT-5-1	2023.077	2.5314296	0.0005857	0.676708	0.44465	m1A-enriched	1.344628	0.10831	0.165436	0.881553	not significant	
tRNA-Thr-TGT-6-1	2003.002	2.5367732	0.0006589	0.673132	0.452617	m1A-enriched	1.359219	0.108045	0.114828	0.9219	not significant	
tRNA-Trp-CCA-2-1	1086.047	2.7449149	0.0023195	0.183535	0.897362	m1A-enriched	1.86237	0.05499	1.909811	0.044517	IgG-enriched	
tRNA-Trp-CCA-4-1	930.7893	2.3160627	9.33E-05	0.775794	0.270652	m1A-enriched	1.894432	0.002119	1.114162	0.098203	IgG-enriched	
tRNA-leader_[4]_tRNA-Pro-AGG-2-1	325.3275	4.7803456	0.0098088	2.886108	0.143575	m1A-enriched	2.824597	0.165573	1.031594	0.6463	not significant	
tRF-1_MT-TV	77.85751	2.8486826	0.2798286	5.625942	0.008754	not significant	3.951321	0.087721	-2.278304	0.481092	m1A-enriched	
tRF-1_tRNA-Leu-AAG-1-2	190.983	2.5308402	0.0244522	0.352869	0.824794	m1A-enriched	0.070504	0.995432	-6.400896	0.001311	not significant	
MT-TH-ENSG00000210176.1	986.602	2.419442	0.1555326	2.562782	0.102529	not significant	2.891989	0.063505	-0.046957	0.998583	m1A-enriched	
MT-TP-ENSG00000210196.2	2952.67	2.3470076	0.07085	-0.866455	0.536704	m1A-enriched	1.62506	0.23123	0.328177	0.841132	not significant	
SNORD124-ENSG00000238793.1	38.29965	20.596742	0.0005157	22.77692	8.26E-05	IgG-enriched	17.16943	0.00528	0	1	m1A-enriched	
SNORD56-ENSG00000229686.1	50.31993	21.191025	0.0004012	22.73579	0.000105	IgG-enriched	20.00442	0.000965	0	1	m1A-enriched	

SNORD86-ENSG00000212498.1	24.82421	3.7675581	0.0401365	-3.191034	0.243355	m1A-enriched	2.691497	0.162436	-2.522506	0.369345	not significant	
miR-10400-5p	766.7396	1.8223302	0.3792921	4.21536	0.010171	not significant	2.933557	0.09747	2.76931	0.114984	m1A-enriched	
miR-10401-5p	35.71212	5.7987504	0.0103647	3.187408	0.228824	m1A-enriched	5.30415	0.019271	6.975253	0.001167	IgG-enriched	
miR-12136-3p	216.4383	8.4441144	0.0402777	0.549017	0.933086	m1A-enriched	6.719595	0.110256	-14.30248	0.000389	not significant	
miR-1249-5p	13.24463	0.4213902	0.9327821	-1.411403	0.729422	not significant	5.153603	0.050967	-0.742876	0.864659	m1A-enriched	
miR-1260a-5p	995.1995	1.4143663	0.0195074	0.810752	0.210721	m1A-enriched	0.03501	0.997856	0.184938	0.815321	not significant	
miR-1260b-5p	134.0936	4.3067842	0.0874801	1.63442	0.547416	m1A-enriched	2.544767	0.348239	0.740191	0.810193	not significant	
miR-137-3p	51.62083	6.2151735	0.0248106	-0.626051	0.897485	m1A-enriched	3.233787	0.299734	-1.396676	0.732774	not significant	
miR-1827-3p	24.53394	-1.639089	0.2729554	-0.2524	0.909107	not significant	3.137101	0.006947	-0.935397	0.654593	m1A-enriched	
miR-194-2-3p	29.25007	4.3475939	0.0525858	-2.971666	0.333514	m1A-enriched	2.813256	0.237416	-0.875864	0.803524	not significant	
miR-194-3p	138.9393	5.5195194	0.0317808	-3.985415	0.232978	m1A-enriched	3.669417	0.181475	3.071961	0.250968	not significant	
miR-211-3p	13.50558	0.5154902	0.9386355	7.760211	0.014461	not significant	7.229153	0.023997	2.511666	0.56108	m1A-enriched	
miR-2110	113.8562	5.6109098	0.063748	2.725573	0.3941	m1A-enriched	4.248253	0.17289	4.07255	0.176219	not significant	
miR-2110-5p	504.6184	5.9385551	2.75E-05	3.48069	0.02648	IgG-enriched	4.785764	0.001179	1.487046	0.414284	m1A-enriched	
miR-2861-3p	40.03348	-2.678965	0.3069245	4.71318	0.020005	not significant	3.598005	0.094315	0.964016	0.736884	m1A-enriched	
miR-296-3p	1593.231	3.9441441	0.0005531	0.126424	0.9537	m1A-enriched	2.096426	0.10656	1.525698	0.241454	not significant	
miR-3136-5p	10.21962	4.1652471	0.0067545	2.341564	0.203802	m1A-enriched	-1.485937	0.487667	-1.017591	0.734576	not significant	
miR-3141	24.10487	8.5542941	0.0004069	7.025893	0.007941	IgG-enriched	10.31406	8.79E-06	0	1	m1A-enriched	
miR-320a-3p	2745.2	2.211816	0.0174299	1.773792	0.061644	IgG-enriched	1.920566	0.041837	1.487273	0.119765	m1A-enriched	
miR-320b	10.06539	2.0713627	0.4932181	3.827819	0.15241	not significant	6.83875	0.001693	1.479324	0.674054	m1A-enriched	
miR-320b-1-3p	2412.429	2.6297967	0.0016367	1.382708	0.139009	m1A-enriched	2.728619	0.000987	1.723833	0.051758	IgG-enriched	
miR-3612-5p	69.04321	5.821828	0.0184617	-2.240046	0.522478	m1A-enriched	4.984474	0.048024	4.591039	0.064779	IgG-enriched	
miR-3911-5p	41.62467	3.8608257	0.0491915	-2.540588	0.379198	m1A-enriched	2.214756	0.30139	-3.312235	0.227967	not significant	
miR-4286-5p	41.82176	1.5794775	0.4966191	-0.839167	0.784093	not significant	4.738628	0.006779	-0.162338	0.971711	m1A-enriched	
miR-4433b-3p	137.7286	5.3036901	0.0188637	-0.467212	0.917335	m1A-enriched	5.49261	0.012653	7.233625	0.000519	IgG-enriched	
miR-4447-3p	16.24951	0.7544465	0.7571521	1.539523	0.429991	not significant	3.402065	0.029673	2.429136	0.180146	m1A-enriched	
miR-4472-2-5p	38.90799	14.142524	0.0015686	0	1	m1A-enriched	15.57363	0.000306	22.50454	2.57E-08	IgG-enriched	
miR-4485-3p	36.24862	6.0866227	0.07085	1.328178	0.776828	m1A-enriched	5.100189	0.132713	-0.433289	0.94048	not significant	
miR-4505-3p	20.65892	5.1938658	0.0948854	2.247675	0.569078	m1A-enriched	-1.12908	0.81713	7.875637	0.004165	not significant	
miR-4633-5p	4545.345	1.7291417	0.0404589	-1.12048	0.197468	m1A-enriched	0.238037	0.854058	-1.303764	0.12191	not significant	
miR-4638-5p	49.28906	4.6295364	0.0342746	-3.196488	0.289846	m1A-enriched	3.303856	0.151492	2.58125	0.269418	not significant	
miR-4651-5p	50.46644	4.1945898	0.0710983	1.590211	0.574204	m1A-enriched	4.09053	0.073308	6.674724	0.001133	IgG-enriched	
miR-4710-5p	24.97871	7.4326275	0.0380652	10.41068	0.001314	IgG-enriched	8.291875	0.015078	0	1	m1A-enriched	
miR-4741-5p	26.3831	3.7999624	0.0102561	-1.349887	0.63585	m1A-enriched	-0.483947	0.850189	-0.681359	0.824598	not significant	
miR-4750-5p	13.76888	6.2661895	0.077148	5.684497	0.126472	m1A-enriched	9.505884	0.00256	6.927801	0.046002	IgG-enriched	
miR-4752-5p	10.12533	1.0621733	0.8861795	1.726244	0.786507	not significant	8.003489	0.068858	2.394769	0.663044	m1A-enriched	
miR-4754-5p	17.62871	2.0525035	0.5981295	6.246463	0.030267	not significant	5.003234	0.098331	0.411238	0.938565	m1A-enriched	
miR-4784-5p	169.0075	3.3358953	0.0177445	4.9595	0.000101	IgG-enriched	3.549281	0.009354	2.22605	0.139537	m1A-enriched	
miR-4787-5p	77.21674	2.6785277	0.0991745	1.3958	0.43261	m1A-enriched	2.864027	0.064067	4.848966	0.000522	not significant	
miR-497-3p	14.17019	1.2964277	0.7010297	5.724173	0.009768	not significant	4.774339	0.035723	2.919062	0.296872	m1A-enriched	
miR-505-5p	141.4236	4.0265643	0.1322163	6.037071	0.009768	not significant	4.906147	0.045513	-1.103709	0.767965	m1A-enriched	
miR-5191-3p	22.90866	-3.607056	0.1778636	4.22146	0.036735	not significant	3.625467	0.082698	-0.308839	0.942901	m1A-enriched	
miR-5193-5p	45.83589	4.9856969	0.036018	3.681606	0.141051	m1A-enriched	4.917538	0.035029	4.251806	0.075891	IgG-enriched	
miR-572-5p	40.02592	3.1788836	0.2852768	0.112263	0.991196	not significant	4.804693	0.060653	3.345262	0.222219	m1A-enriched	
miR-6074-3p	218.8469	4.5747522	2.99E-17	0.461194	0.613834	m1A-enriched	3.113803	2.68E-08	2.957554	7.80E-07	IgG-enriched	
miR-6076-5p	33.2383	6.4683248	0.0098088	3.169927	0.30369	m1A-enriched	2.366137	0.440788	8.298346	0.00041	not significant	
miR-6084-5p	127.0902	4.6134573	1.08E-08	-1.097615	0.408398	m1A-enriched	1.188571	0.261188	-4.454247	0.027483	not significant	
miR-627-5p	33.56549	2.1062781	0.5403599	6.366683	0.012161	not significant	5.179755	0.050967	-0.39116	0.937641	m1A-enriched	
miR-632-5p	51.17592	2.5640323	0.2493296	6.485741	0.000139	not significant	4.483243	0.014462	2.988285	0.159125	m1A-enriched	
miR-658-3p	88.34684	1.9047688	0.5759727	3.980418	0.13979	not significant	5.423055	0.035315	3.299126	0.224834	m1A-enriched	
miR-6717-5p	179.0983	3.0921978	0.0271556	-0.155997	0.948226	m1A-enriched	1.234453	0.455396	1.771063	0.226893	not significant	
miR-6745-3p	33.45394	2.3996095	0.3927793	5.387556	0.016355	not significant	4.741376	0.039993	2.033638	0.472218	m1A-enriched	
miR-6747-5p	15.57647	1.6214373	0.2883231	-2.079023	0.39917	not significant	2.518054	0.054479	-1.41764	0.577306	m1A-enriched	
miR-6752-5p	15.70285	5.0818858	0.1179617	3.726042	0.264997	not significant	6.962125	0.016411	3.81496	0.265121	m1A-enriched	
miR-6756-5p	62.51858	5.0253918	0.0060254	1.027313	0.696673	m1A-enriched	4.06845	0.030977	5.088301	0.00489	IgG-enriched	
miR-6787-5p	65.56582	4.5053909	0.1185438	4.609938	0.090162	not significant	4.550164	0.098716	-0.246577	0.970182	m1A-enriched	
miR-6802-5p	105.4938	3.994518	0.0609549	2.978447	0.19408	m1A-enriched	5.927107	0.001882	7.705678	2.09E-05	IgG-enriched	
miR-6808-5p	18.29334	2.1777834	0.1495739	-1.452568	0.583488	not significant	3.511446	0.006779	0.247108	0.928957	m1A-enriched	
miR-6819-5p	24.92309	6.8474409	0.0554958	8.519936	0.01043	IgG-enriched	7.916131	0.01807	5.345376	0.184599	m1A-enriched	
miR-6870-5p	41.2995	4.2212813	0.2871194	6.149278	0.074362	not significant	7.833872	0.016681	3.376426	0.397269	m1A-enriched	
miR-7706	224.7456	3.608769	0.0760046	0.815573	0.750258	m1A-enriched	1.433286	0.54533	-4.490117	0.091076	not significant	
miR-7977-5p	209.9544	4.34303	0.0489875	-4.281928	0.142918	m1A-enriched	1.894157	0.453442	2.192818	0.346715	not significant	
miR-920-3p	51.51468	4.2326527	0.3290277	7.162093	0.049425	not significant	6.460367	0.086618	2.247299	0.63261	m1A-enriched	
miR-92b-3p	19207.76	3.1070137	0.0559199	2.123608	0.196791	m1A-enriched	2.636649	0.105018	1.664293	0.321735	not significant	
miR-9901-5p	14.98482	6.0099417	0.0757235	4.59316	0.18513	m1A-enriched	5.375182	0.110332	2.440761	0.563668	not significant	

Supplementary Table 3. Mismatch analysis for tRF-3b in Input and Ago2-bound fractions by TGIRT-seq

m1A4 mismatch% (Mean of 3 reps)	Input_siCtrl	Input_siTRMT6/61A	FHAgo2_siCtrl	FHAgo2_siTRMT6/61A
tRF-3b: Ala	52.64%	32.98%	54.73%	37.70%
tRF-3b: Arg	91.76%	44.13%	76.97%	32.43%
tRF-3b: Gly	83.99%	52.04%	78.44%	59.49%
tRF-3b: Gln	59.31%	53.49%	60.85%	57.94%
tRF-3b: His	81.24%	42.76%	70.90%	37.00%
tRF-3b: Leu	90.15%	53.28%	77.99%	50.72%
tRF-3b: Pro	85.26%	55.44%	82.38%	77.15%
tRF-3b: Phe	91.00%	84.04%	78.56%	67.87%
tRF-3b: Ser	93.54%	79.18%	90.23%	75.18%
tRF-3b: Thr	83.18%	44.83%	75.57%	54.29%
tRF-3b: Trp	86.24%	68.21%	89.68%	64.25%
tRF-3b: Tyr	81.37%	57.63%	75.19%	56.97%
tRF-3b: Val	67.45%	43.16%	65.46%	48.68%
tRF-3b: iMet	76.90%	73.75%	67.50%	70.72%

Supplementary Table 4. Seed clusters of Ago2-bound tRF-3bs and their mismatch analysis upon siTRMT6/61A - related to Fig. 5

Seed#	seed sequence (1-8nt)	tRF-3b (parental tRNAs)	m1A4 mismatch% by TGIRT-seq		
			siCtrl_Ago2 (mean of 3 reps)	si661A_Ago2 (mean of 3 reps)	Log2FoldChange (si661AvsCtrl)
Seed5	TCGAAACC	iMet, Val	68.26%	71.09%	0.06
Seed1	TCGAATCC	Leu, Ser, Tyr, Arg, His, Thr	87.27%	67.34%	-0.37
Seed13	TCGAATCA	Trp	90.99%	61.16%	-0.57
Seed2	TCAAATCT	Gln, Thr	66.15%	58.24%	-0.18
Seed7	TCAAATCC	Pro, Cys, Leu	82.10%	73.95%	-0.15
Seed3	TCGATTCC	Tyr, Gly	72.44%	47.92%	-0.60

Supplementary Table 5. List of tRF-3b targets down-regulated by siTRMT61A - related to Fig. 6

tRF-3b predicted targets (8/7mer)			RNA-seq HEK293T siTRMT61A/siCtrl (DESeq2)			
**Gene**	**Seed_family**	**Site_type**	**baseMean**	**log2FoldChange**	**pvalue**	**padj**
TIMP3	seed2, seed2	7mer-m8, 7mer-m8	853.29	-1.25	2.57E-38	3.34E-34
CREB3L2	seed2, seed2, seed2, seed7, seed7, seed7	7mer-1a, 8mer-1a, 7mer-m8, 8mer-1a, 7mer-m8, 7mer-1a	1763.63	-0.73	4.86E-21	1.58E-17
MBTPS1	seed2, seed7	8mer-1a, 7mer-1a	2315.71	-0.73	3.57E-19	9.25E-16
CDK19	seed2	7mer-m8	232.14	-1.36	1.66E-17	3.07E-14
HERPUD1	seed2, seed7	7mer-1a, 7mer-1a	1213.88	-0.63	2.15E-12	2.32E-09
ELMOD2	seed2, seed7	7mer-1a, 8mer-1a	869.50	-1.01	5.63E-12	5.62E-09
RMND5A	seed2, seed2	7mer-m8, 7mer-m8	2004.49	-0.63	1.87E-10	1.73E-07
HIPK2	seed2, seed2, seed7, seed7	7mer-1a, 8mer-1a, 7mer-1a, 7mer-1a,	1282.75	-0.59	1.15E-08	6.47E-06
HLTF	seed2, seed7	7mer-1a, 7mer-1a	3622.88	-0.68	2.25E-08	1.04E-05
FKTN	seed2, seed7	7mer-m8, 7mer-m8	944.52	-0.72	1.34E-08	6.66E-06
NRAS	seed7	7mer-m8	2057.65	-0.65	1.29E-08	6.66E-06
SUZ12	seed2, seed7	7mer-1a, 7mer-1a	1622.96	-0.56	1.27E-08	6.66E-06
DUSP12	seed2	7mer-m8	529.57	-0.68	4.32E-08	1.78E-05
CTDSPL2	seed2, seed7, seed7	7mer-1a, 7mer-m8, 7mer-1a	1089.17	-0.67	1.45E-07	4.71E-05
SERTAD2	seed2, seed7, seed7	8mer-1a, 7mer-m8, 7mer-1a	463.26	-0.63	1.43E-07	4.71E-05
RHOU	seed2, seed7	7mer-1a, 7mer-1a	402.46	-0.70	1.65E-07	4.97E-05
SP3	seed2, seed2, seed2, seed3, seed7	7mer-1a, 7mer-m8, 7mer-m8, 7mer-1a, 8mer-1a	1415.53	-0.56	1.65E-07	4.97E-05
EHD3	seed7	7mer-m8	231.78	-0.88	2.10E-07	6.20E-05
TMED7	seed7	7mer-m8	1328.00	-0.58	3.99E-07	0.000112
RBMS1	seed2, seed2	7mer-m8, 7mer-m8	695.42	-0.63	2.24E-06	0.00052
FOXP2	seed2	7mer-m8	491.68	-0.58	5.18E-06	0.001012
TMEM87B	seed2, seed2, seed2, seed7, seed7, seed7, seed7	7mer-1a, 7mer-1a, 7mer-1a, 7mer-1a, 7mer-1a, 7mer-m8, 7mer-1a	284.52	-0.65	8.23E-06	0.001442
ROCK2	seed2, seed7	7mer-1a, 7mer-1a	1176.53	-0.56	9.35E-06	0.001574
NFIB	seed7	7mer-m8	261.72	-0.62	1.83E-05	0.002695
VASH2	seed2, seed7	7mer-1a, 7mer-1a	328.88	-0.62	2.59E-05	0.00361
NAPG	seed2	7mer-m8	352.55	-0.57	4.28E-05	0.005424
TSKU	seed2, seed7, seed7, seed7	7mer-1a, 7mer-1a, 7mer-m8, 7mer-m8	199.51	-0.75	4.70E-05	0.005799
PPM1H	seed7	7mer-m8	429.65	-0.54	8.25E-05	0.00891
IGFBP5	seed2, seed2, seed7, seed7	7mer-1a, 7mer-1a, 8mer-1a, 8mer-1a	306.25	-0.54	0.000106	0.010615
IFT80	seed7	7mer-m8	637.89	-0.52	0.000424	0.030673
RGS17	seed1, seed1, seed13, seed2, seed7	7mer-m8, 7mer-1a, 7mer-1a, 8mer-1a, 7mer-1a	209.28	-0.54	0.000556	0.035678

Supplementary Table 6. BLCA patients in this study - related to Fig. 7

Patient#	Gender	Age	Primary or Recidive
#1	Female	50	Primary
#2	Male	78	Primary
#3	Male	79	Primary
#4	Male	69	Primary
#5	Male	75	Primary

Supplementary Table 7. List of RNA/DNA oligonucleotides in this study

Synthetic RNA oligos	
Control1	/5Phos/-rCrGrUrArCrGrCrGrGrArArUrArCrUrUrCrGrArUrU-/3OH/
Control1-m1A	/5Phos/-rCrGrU/i1Me-rA/rCrGrCrGrGrArArUrArCrUrUrCrGrArUrU-/3OH/
Control2	/5Phos/-rArCrUrcrUrArUrCrCrCrGrArCrCrCrCrGrArCrGrCrU-/3OH/
Control2-m1A	/5Phos/-rArCrUrcrU/i1Me-rA/rUrCrCrCrGrArCrCrCrCrGrArCrGrCrU-/3OH/
NT1 (non-targeting)	/5Phos/-rUrArCrGrGrArCrUrUrArArGrCrGrGrCrUrArCrArUrA-/3OH/
NT2 (non-targeting)	/5Phos/-rCrGrUrArCrGrCrGrGrArArUrArCrUrUrCrGrArUrU-/3OH/
tRF-3009b	/5Phos/-rUrCrGrArArCrCrCrCrArCrUrCrCrUrGrGrUrArCrCrA-/3OH/
tRF-3009b-m1A	/5Phos/-rUrCrG/i1Me-rA/rArCrCrCrCrArCrUrCrCrUrGrGrUrArCrCrA-/3OH/
tRF-3021b	/5Phos/-rUrCrGrArUrCrCrCrCrGrGrCrArUrCrUrCrCrArCrCrA-/3OH/
tRF-3021b-m1A	/5Phos/-rUrCrG/i1Me-rA/rUrCrCrCrCrGrGrCrArUrCrUrCrCrArCrCrA-/3OH/
tRF-3030b	/5Phos/-rUrCrGrArUrUrCrCrGrGrCrUrCrGrArArGrGrArCrCrA-/3OH/
tRF-3030b-m1A	/5Phos/-rUrCrG/i1Me-rA/rUrUrCrCrGrGrCrUrCrGrArArGrGrArCrCrA-/3OH/
tRF-3004b	/5Phos/-rUrCrArArArUrCrUrCrGrGrUrGrGrArArCrCrUrCrCrA-/3OH/
tRF-3004b-m1A	/5Phos/-rUrCrA/i1Me-rA/rArUrCrUrCrGrGrUrGrGrArArCrCrUrCrCrA-/3OH/
DNA oligos for RT-qPCR (cell line)	
ACTB (forward)	5'-GCACTCTTCCAGCCTTCCTT-3'
ACTB (reverse)	5'-GACAGCACTGTGTTGGCGTA-3'
TRMT6 (forward)	5'-GGTGCTGAAACGTGAAGATGT-3'
TRMT6 (reverse)	5'-CTTGGGCTGTAGACTTCCTCC-3'
TRMT61A (forward)	5'-AGCTTCGTGGCATACGAGG-3'
TRMT61A (reverse)	5'-CCGATAAGGTCAACTGAGTGC-3'
TIMP3 (forward)	5'-CATGTGCAGTACATCCATACGG-3'
TIMP3 (reverse)	5'-CATCATAGACGCGACCTGTCA-3'
CREB3L2 (forward)	5'-ACCACACGCACTTCTCAGAAC-3'
CREB3L2 (reverse)	5'-GAGGAAAGGATCATTCAGGAGC-3'
MBTPS1 (forward)	5'-ACCTCGAAACAATCCATCCAGT-3'
MBTPS1 (reverse)	5'-ACTTGAGGGAACGAAAGACTTTT-3'
CDK19 (forward)	5'-GGATTTGTTTGAGTACGAAGGGT-3'
CDK19 (reverse)	5'-CTACAAGCCGACATGGATATTCC-3'
HIPK2 (forward)	5'-CCCGTGTACGAAGGTATGGC-3'
HIPK2 (reverse)	5'-AGTTGGAACTCGGCTCTATTTTC-3'
HERPUD1 (forward)	5'-TGCTGGTTCTAATCGGGGACA-3'
HERPUD1 (reverse)	5'-CCAGGGGAAGAAAGGTTCCG-3'
RMND5A (forward)	5'-TGATGGTGGAGCACTTCTTTC-3'
RMND5A (reverse)	5'-CCAGAGCAGGTCTCAGAACTC-3'
ELMOD2 (forward)	5'-GATGACTGGGAAGTGTGAATTGC-3'
ELMOD2 (reverse)	5'-TGGCATCCTTCTCAGGGTTAATA-3'
HLTF (forward)	5'-TTTTCCACGCCTCTCATATCCA-3'
HLTF (reverse)	5'-AGCGTAGTCCAACCACATGAC-3'
DNA oligos for RT-qPCR (patient samples)	
ACTB (forward)	5'-ACAGAGCCTCGCCTT-3'
ACTB (reverse)	5'-CGCGGCGATATCATC-3'
TRMT6 (forward)	5'-AAGGGGCCTAAAGAGAGAGGA-3'
TRMT6 (reverse)	5'-TAGCTACAATTAAACCATCTGCGT-3'
TRMT61A (forward)	5'-ACAGACATCGCCCTCATCAC-3'
TRMT61A (reverse)	5'-GATGATGGCGTGGGACACAG-3'
DNA oligos for Northern blot	
U6	5'-BIO-TGCGTGTCATCCTTGCGCAG-3'
tRNA/tRF-3021(Ala)	5'-BIO-TGGTGGAGATGCCGGGGATC-3'
tRNA/tRF-3030(Tyr)	5'-BIO-TGGTCCTTCGAGCCGGAATC-3'
tRNA/tRF-3004(Gln)	5'-BIO-TGGAGGTTCCACCGAGAT-3'
LNA/DNA oligos for tRF knock-down (+ indicates LNA bases)	
Ctrl LNA	+C+G+T+ACGCGGAATAC+T+T+C+G
anti-tRF3009	TGGTACCA+GG+AG+TGG+GG+T
anti-tRF3021	+G+G+T+GGAGATGCCG+G+G+G+A
anti-tRF3030	+G+G+T+CCTTCGAGCC+G+G+A+A
anti-tRF3004	+G+G+A+GGTTCCACCG+A+G+A+T
