# Supplementary material for: TRMT6/61A-dependent base methylation of tRNA-derived fragments regulates gene-silencing activity and the unfolded protein response in bladder cancer
Source: Nat Commun. 2022 Apr 20;13:2165. doi: 10.1038/s41467-022-29790-8 (PMC9021294; doi:10.1038/s41467-022-29790-8)
Supplement: Supplementary file 2 — Reporting Summary [file 41467_2022_29790_MOESM2_ESM.pdf]

Corresponding author(s): Anindya Dutta; Rune Ougland

Last updated by author(s): Feb 12, 2022

Reporting Summary

Nature Research wishes to improve the reproducibility of the work that we publish. This form provides structure for consistency and transparency in reporting. For further information on Nature Research policies, see our Editorial Policies and the Editorial Policy Checklist.

Statistics

For all statistical analyses, confirm that the following items are present in the figure legend, table legend, main text, or Methods section.

n/a	Confirmed	
☐	☒	The exact sample size (*n*) for each experimental group/condition, given as a discrete number and unit of measurement
☐	☒	A statement on whether measurements were taken from distinct samples or whether the same sample was measured repeatedly
☐	☒	The statistical test(s) used AND whether they are one- or two-sided<br>*Only common tests should be described solely by name; describe more complex techniques in the Methods section.*
☒	☐	A description of all covariates tested
☐	☒	A description of any assumptions or corrections, such as tests of normality and adjustment for multiple comparisons
☐	☒	A full description of the statistical parameters including central tendency (e.g. means) or other basic estimates (e.g. regression coefficient) AND variation (e.g. standard deviation) or associated estimates of uncertainty (e.g. confidence intervals)
☐	☒	For null hypothesis testing, the test statistic (e.g. *F*, *t*, *r*) with confidence intervals, effect sizes, degrees of freedom and *P* value noted<br>*Give P values as exact values whenever suitable.*
☒	☐	For Bayesian analysis, information on the choice of priors and Markov chain Monte Carlo settings
☒	☐	For hierarchical and complex designs, identification of the appropriate level for tests and full reporting of outcomes
☐	☒	Estimates of effect sizes (e.g. Cohen's *d*, Pearson's *r*), indicating how they were calculated

Our web collection on statistics for biologists contains articles on many of the points above.

Software and code

Policy information about availability of computer code

Data collection	TCGA data from GEPIA2, TCGAbiolinks v2.20.1
Data analysis	cutadapt v1.15, unitas v1.7.3, kallisto v0.46.1, COOT v0.8.9.1, MacPyMOL v1.7.0.3, GraphPad Prism v9.1.0, ImageLab v5.2.1

For manuscripts utilizing custom algorithms or software that are central to the research but not yet described in published literature, software must be made available to editors and reviewers. We strongly encourage code deposition in a community repository (e.g. GitHub). See the Nature Research guidelines for submitting code & software for further information.

Data

Policy information about availability of data

All manuscripts must include a data availability statement. This statement should provide the following information, where applicable:

- Accession codes, unique identifiers, or web links for publicly available datasets
- A list of figures that have associated raw data
- A description of any restrictions on data availability

The small RNA-seq and RNA-seq data generated in this study has been deposited in the Gene Expression Omnibus (GEO) database under accession code GSE171040 [https://www.ncbi.nlm.nih.gov/geo/query/acc.cgi?acc=GSE171040]. The raw sequencing data for small RNA-seq and RNA-seq in BLCA patients are protected and are not available due to data privacy laws. The processed data (mapped gene counts) may be available upon reasonable request and pending project-specific ethics approval and after ratification of required legal agreements. Requests should be directed to Rune Ougland (runoug@vestreviken.no). The co-crystal structure of human Ago2 and single-stranded guide RNA used in this study is available in the PDB database under accession code 5js1 [https://10.2210/pdb5JS1/pdb]. TCGA BLCA analysis was based on GEPIA2 Expression Analysis (https://gepia2.cancer-pku.cn/). Source data are provided with this paper.

Field-specific reporting

Please select the one below that is the best fit for your research. If you are not sure, read the appropriate sections before making your selection.

☒ Life sciences ☐ Behavioural & social sciences ☐ Ecological, evolutionary & environmental sciences

For a reference copy of the document with all sections, see nature.com/documents/nr-reporting-summary-flat.pdf

Life sciences study design

All studies must disclose on these points even when the disclosure is negative.

Sample size	No statistical method was performed to pre-determine the sample size. Sample size of five were used for bladder patient sequencing analysis, due to the nature of paired samples.
Data exclusions	No data exclusions
Replication	Multiple measures were used: (1) two different m1A antibodies were used to replicate the m1A RIP, each antibody was used in duplicate experiments with consistent results; (2) TRMT6/61A knock-down experimentswere repeated more than three times independently with consistent results; (3) qPCR and luciferase assays were repeated more than three times independently with consistent results; (4) small RNA-seq or RNA-seq were done with at least duplicates per condition with consistent results.
Randomization	Bladder tissue specimens were obtained from patients undergoing transurethral resection of bladder tumors (TURBT) at Vestre Viken Hospital Trust following written informed consent. We compared tumor tissues with normal tissue from the same patient. We did not compare any medication or treatment, thus no randomization was required.
Blinding	Investigators were blinded during universal data analysis (mapping, quantification). Physicians were blinded to patient information during assessment.

Reporting for specific materials, systems and methods

We require information from authors about some types of materials, experimental systems and methods used in many studies. Here, indicate whether each material, system or method listed is relevant to your study. If you are not sure if a list item applies to your research, read the appropriate section before selecting a response.

Materials & experimental systems

n/a	Involved in the study
☐	☒ Antibodies
☐	☒ Eukaryotic cell lines
☒	☐ Palaeontology and archaeology
☒	☐ Animals and other organisms
☐	☒ Human research participants
☒	☐ Clinical data
☒	☐ Dual use research of concern

Methods

n/a	Involved in the study
☒	☐ ChIP-seq
☒	☐ Flow cytometry
☒	☐ MRI-based neuroimaging

Antibodies

Antibodies used	Cell line work: m1A antibody (MBL #D3453, or Abcam #ab208196). β-actin (SCBT #sc-47778), TRMT6 (SCBT #sc-271752). M2 FLAG affinity gel (Sigma #A2220), MYC mouse antibody 9E10 (homemade). Secondary antibody: anti-mouse HRP-linked secondary antibody (Cell Signaling #7076).<br>Patient work: : β-actin (Abcam #ab8224, mouse monoclonal), TRMT6 (Abcam #ab235321, rabbit polyclonal), and TRMT61A (Biorbyt #orb411814, rabbit polyclonal). Secondary antibody: Anti-mouse (BioNordica #PI-2000) and anti-rabbit (GE Healthcare Life Sciences #NA934-100UL) HRP-linked secondary antibodies.
Validation	1. m1A antibody (MBL #D3453): this antibody was validated for ELISA, ICC, IHC, IP by manufacturer (https://www.mblintl.com/products/d345-3/).<br>2. m1A antibody (Abcam #ab208196): this recombinant antibody was validated for IP, Dot blot, ELISA by manufacturer (https://www.abcam.com/1-methyladenosine-m1a-antibody-epr-19836-208-ab208196.html). Manufacturer tested dot blot with competition nucleosides, and tested IP with LC-MS/MS.<br>In addition, m1A antibodies (MBL #D3453, abcam #ab208196) were previously validated by competition assay (PMID: 31719534).<br>3. TRMT6 antibody (SCBT #sc-271752) was validated for WB, IP, IF, IHC and ELISA by manufacturer (https://www.scbt.com/p/trmt6-antibody-f-3). In addition, it is validated by siTRMT6 knockdown in this paper (Fig. 3).<br>4. β-actin (SCBT #sc-47778) was validated for WB, IP, IF, IHC and ELISA by manufacturer (https://www.scbt.com/p/beta-actin-antibody-c4).<br>5. β-actin (Abcam #ab8224) was validated for WB, IHC and flow cytometry by manufacturer (https://www.abcam.com/beta-actin-

antibody-mabcam-8224-loading-control-ab8224.html).
6. TRMT6 (Abcam #ab235321) was validated for WB and IHC by manufacturer (https://www.abcam.com/trm6-antibody-ab235321.html).
7. TRMT61A (Biorbyt #orb411814) was validated for WB by manufacturer (https://www.biorbyt.com/trmt61a-antibody-orb411814.html).

Eukaryotic cell lines

Policy information about cell lines

Cell line source(s)	HEK293T was obtained from ATCC (ATCC #CRL-3216) and similarly for HeLa (ATCC #CCL-2). U251 was a kind gift from Roger Abounader. T24 bladder cancer cell line was obtained from ATCC (ATCC #HTB-4).
Authentication	Purchased from source without authentication.
Mycoplasma contamination	Mycoplasma contamination was routinely checked by PCR kit (SouthernBiotech #13100-01) and all cell lines were tested negative.
Commonly misidentified lines (See ICLAC register)	None of the cell lines are in the "Misidentified Cell Line" list by ICLAC v11.

Human research participants

Policy information about studies involving human research participants

Population characteristics	Patient information is included in Supplementary Table 6, including gender (four male and one female), age (50-79) and tumor type (all primary tumor).
Recruitment	Bladder tissue specimens were obtained from patients undergoing transurethral resection of bladder tumors (TURBT) at Vestre Viken Hospital Trust following written informed consent without participant compensation. Only patients with non-muscle invasive papillary urothelial carcinomas were included. We are not aware of any self-selection bias or other biases that may be present.
Ethics oversight	All procedures and analyses were done in accordance with the study protocol approved by Vestre Viken Hospital Trust and The Regional Ethics Committee South-Eastern Norway Regional Health Authority (reference #2017/2170).

Note that full information on the approval of the study protocol must also be provided in the manuscript.
